# Efficient mechanochemical synthesis of regioselective persubstituted cyclodextrins

**DOI:** 10.3762/bjoc.12.230

**Published:** 2016-11-10

**Authors:** Laszlo Jicsinszky, Marina Caporaso, Katia Martina, Emanuela Calcio Gaudino, Giancarlo Cravotto

**Affiliations:** 1 Department of Drug Science and Technology and NIS - Centre for Nanostructured Interfaces and Surfaces, University of Turin, Via P. Giuria 9, 10125 Turin (Italy)

**Keywords:** green chemistry, nucleophilic substitution, planetary ball mill, siRNA delivery intermediate, sugammadex

## Abstract

A number of per-6-substituted cyclodextrin derivative syntheses have been effectively carried out in a planetary ball mill under solvent-free conditions. The preparation of Bridion^®^ and important per-6-amino/thiocyclodextrin intermediates without polar aprotic solvents, a source of byproducts and persistent impurities, could be performed. Isolation and purification processes could also be simplified. Considerably lower alkylthiol/halide ratio were necessary to reach the complete reaction in comparison with thiourea or azide reactions. While the presented mechanochemical syntheses were carried out on the millimolar scale, they are easily scalable.

## Introduction

Cyclodextrins (CDs) are cyclic α(1→4)glucopyranosides and have been fully described in a number of publications [1–3]. They are most noted for their ability to form non-covalent associations called “inclusion complexes”. Natural CDs exhibit many favourable properties, which advance their use in a wide range of applications. However, syntheses for many special applications, such as DNA sequencing [4–5], gene delivery [6], and drug targeting hosts [7–8], can be problematic as they require sophisticated, efficient and yet simple methods which lead to acceptable purity and impurity profiles. Furthermore, the special structural properties of selectively substituted CDs mean that their syntheses are not always environmentally friendly procedures. Ball mill assisted syntheses are good alternatives to overcome solubility difficulties in syntheses or isolation of natural compounds from vegetables using CDs [9–10]. Environmentally benign synthetic methods of CD derivatives have been recently reviewed [11].

The key intermediate in the bulk preparation of selectively per-6 substituted CDs is the per-6-deoxy-6-halide derivative, which covers per-6-bromo [12] and per-6-iodo [13] compounds. Although per-6-chloro-CD derivatives can also be easily prepared [14] but the lower reactivity of chloro compounds restricts the use of per-6-chloro-CDs [15] in solution. The solubility of per-6-halogeno-CDs is very limited in water and the majority of organic solvents, meaning that their preparation and purification is far from being environmentally friendly.

Per-6-*S*-(3-mercapto)propionyl-γ-CD (Sugammadex, Bridion^®^) is not only the biggest success that CD derivatization has had, but its use in the removal of muscle relaxants may revolutionize surgery. It has very high affinity with curare analogues, especially rucoronium (*K*_11_ ≈ 1.8 × 10^7^ M^−1^) [7], which are widely used in surgery [6]. Its everyday use has led to increasing demand and ever higher amounts being employed, meaning it will likely soon become a generic molecule in most hospitals. The solventless preparation technique can provide clear advantages over classic methods since its use in humans requires very high purity. The standard preparation of Sugammadex uses a harsh base, such as sodium hydride, to activate the thiol group of 3-mercaptopropionic acid (MPA) and *N,N*-dimethylformamide (DMF) [16]. In the reaction solution and particularly on larger scales (from 10 g to kilo lab scale), impurities that arise from incomplete conversions, product and byproduct decomposition as well as solvent impurities increase synthesis and purification costs and time.

Selectively per-6-thiolated CDs are also used in gold nanoparticle chemistry, particularly in electrochemical sensors [17]. Thiourea (TU) is one of the best precursors of those CDs because as the halogen is exchanged to the thiouronium salt of CDs thiols can readily be obtained under aqueous basic conditions. Thioureido-CDs are crystalline compounds that are readily soluble in water, easy to purify and convert to thiols [8]. These intermediates can be efficiently prepared via the reaction of a TU excess (2–3 mol TU/halogen) and per-6-halogenated CDs. The preparation is also carried out in a polar aprotic solvent, as in case of the azide exchange, while byproducts may also be similar despite the higher nucleophilicity of sulfur. The reaction under classic conditions usually requires large TU excess.

Various per-6-alkylthio-β-CD derivatives are used in siRNA delivery and gene therapy [6]. The alkyl chain, usually C_10_–C_16_, makes the product very amphiphilic, which means that it is difficult to purify not only from possible byproducts but also from the reagents. Additionally, sulfur-containing organic compounds can form very strong complexes with not only the native but with the substituted CDs, too. Although a solvent-free synthetic method does not solve the problem of complexation but the reduced amount of reagents can simplify the purification.

Heptakis(6-azido-6-deoxy)-β-CD is the precursor to per-6-amino-β-CD, which is an important component of DNA sequencing equipment [4]. *p*-Toluenesulfonyl (Ts) esters are still an irreplaceable leaving group for carbohydrates as well. The Ts → azide exchange in Ts-β-CD is effective in both solution and under high-energy ball milling (HEBM) conditions, whereas it is difficult in the per-halide analogues, because of sodium azide’s poor solubility in DMF, *N,N*-dimethylacetamide, *N*-methylpyrrolidone and dimethyl sulfoxide. A solution to this problem might be found in the successive addition of sodium azide, considerably longer reaction times and/or higher temperatures (≈100 °C) for the complete reaction. Per-6-bromo- and per-6-iodo-CDs are inevitably able to react with dimethylamine, a common decomposition product of DMF. While the formation of dimethylamine-moiety-containing CDs is virtually negligible – thankfully, as it can cause serious problems in pharmaceutical or biological preparations – its physicochemical properties are very similar to those of the perazido derivatives, making separation impossible. The similarity is even higher after the azide’s conversion to an amine. Solvent removal in the synthesis of per-6-azido-CDs is generally also challenging because of the high boiling polar aprotic solvents used; their complete removal is a difficult task under laboratory conditions, even at high vacuum.

Ball milling is an effective method for the preparation of inclusion complexes which has recently begun to be appreciated by organic chemists for its simplicity and flexibility [18–21]. While the mechanochemical manipulation of covalent bonds is hardly a brand new concept, its diffusion into carbohydrate chemistry, and particularly into CD derivatization, has been rather slow [22–23]. The ability of HEBM to favour the nucleophilic substitution reaction of 6^I^-monotosyl-β-CD has been demonstrated in a previous article [22]. Most interestingly, the side reactions that appear to be unavoidable in the classical solution syntheses can be eliminated in the solvent-free method described for the preparation of various CD derivatives. HEBM was found to be particularly efficient when good nucleophiles, such as sulfur-containing reactants or inorganic azides, were used.

**Scheme 1:**
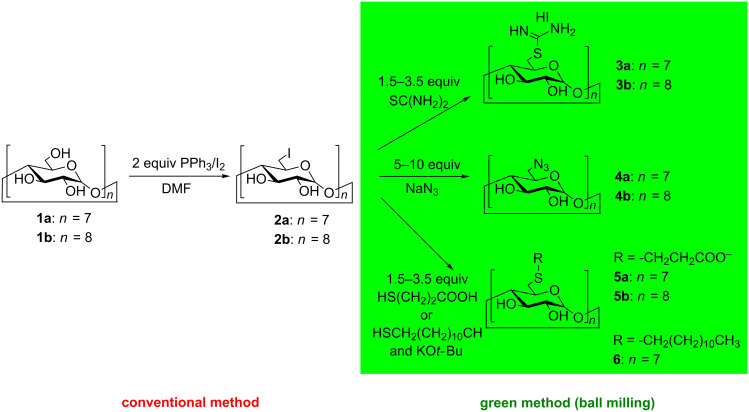
Synthesis of *per*-6-derivatized CDs. Ball milling conditions: 1500 steel balls of 1 mm diameter and 50 steel balls of 5 mm diameter, sun wheel speed 650 min^−1^, 2 h grinding.

The aim of our study is to highlight the use of HEBM in the preparation of a number of practically important CD derivatives, as seen in Scheme 1. Although in our work neither the reaction conditions nor the purifications were optimized in any terms the presented results can serve as starting point to develop more environmentally benign synthetic methods of important CD derivatives. The well-established engineering of HEBM reactions makes them easy to scale-up, while simplified work-up procedures can further reduce the presence of unwanted byproducts which explains our interest in the preparation of compounds such as **3a**, **5b**, and **6** (Scheme 1).

## Results and Discussion

Per-6-iodinated and, in some cases, per-6-brominated CD derivatives are the most common activated per-6-CDs. Their synthesis can be performed on large scales under safe conditions. However, there can be some difficulties (compounds **3** and **4**, entries 2–4, 7–11 in Table 1) in reaction and during purification, meaning that we therefore also decided to test per-6-chloro-β-CD from which the more ionic TU salt can be formed and the chloride has less affinity to the macrocycle. It was not possible to reproduce the literature method [14] for per-6-chloro-β-CD synthesis, but a protocol using *p*-toluenesulfonyl chloride under reaction conditions that were analogous to the iodination/bromination reaction resulted in the targeted heptakis(6-chloro-6-deoxy)-β-CD being produced in good yields [20]. Not unexpectedly, per-6-chlorinated-β-CD showed very poor reactivity, not only under classic solvent reaction conditions (entry 14 in Table 1), but also under ball milling (entries 5 and 15 in Table 1).

**Table 1:** Comparison of classic and green methods for the preparation of CD derivatives.

Entry	Compound	Reagent/solvent	Reagent/ halogen molar ratio	Method	Batch size [mmol]	Yield^a^ [%]	Final temp. [°C]	React. time [h]

1	**3a**	thiourea/DMF	2	soln.	7.5	75	80	3 [27]
2		thiourea	1.5	BM	0.1	12	89	2
3		thiourea	3.5	BM	0.1	25	89	2
4		thiourea	3.5	BM	1	61	88	2
5		thiourea^b^	3.5	BM	0.1	traces^c^	92	2
6	**3b**	thiourea/DMF	2	soln.	5	90	80	16
7		thiourea	1.5	BM	0.1	14^c,d^	85	2
8		thiourea^e^	1.5	BM	0.1	9^c,d^	82	2
9		thiourea	3.5	BM	0.05	33^d^	85	2
10		thiourea^e^	3.5	BM	0.05	39^d^	82	2
11		thiourea^e^	3.5	BM	0.5	57^d^	86	2
12	**4a**	NaN_3_/DMF	1.25	soln.	5	90	100–105	~4.5
13		NaN_3_/DMF	1.25	soln.	0.5	76	100–105	5
14		NaN_3_/DMF^b^	1.25	soln.	0.5	traces^c^	100–105	5 [24]
15		NaN_3_^b^	5	BM	0.1	traces^c^	86	2
16		NaN_3_	5	BM	0.1	69	88	2
17		NaN_3_	5	BM	0.5	72	90	2
18	**4b**	NaN_3_/DMF	1.25	soln.	5	84	100–105	≈4.5
19		NaN_3_	10	BM	0.05	67	82	2
20		NaN_3_	10	BM	0.5	71	84	2
21	**5a**	MPA/Cs_2_CO_3_/DMF	1.5	soln.	5	60	50	25 [28]
22		MPA/KO*t*-Bu^f^	1.43	BM	0.1	86	72	2
23		MPA/KO*t*-Bu	1.5	BM	1	71	75	2
24	**5b**	MPA/NaH/DMF	1.25	soln.	1.4	60	70	12 [16]
25		MPA/TEA/DMF^f^	3	soln.	2.5	60^c^	60	24
26		MPA/KO*t*-Bu	1.25	BM	0.05	81	73	2
27		MPA/KO*t*-Bu^e^	1.5	BM	0.05	86	71	2
28		MPA/KO*t*-Bu^e^	1.5	BM	0.5	72^d^	76	2
29	**6**	DDS/K *t*-Bu/DMF	no data	soln.	1	>90	80	96 [29]
30		DDS/KO*t*-Bu/DMF^g^	3	soln.	0.5	83	80	120
31		DDS/KO*t*-Bu^g^	1.6	BM	0.1	95	62	2

^a^Isolated yields but due to small batch-sizes and not optimized purifications the yields of BM reactions are informative only. ^b^From per-6-chloro-β-CD. ^c^Contains also incompletely substituted structures, further isolation/purification were not performed. ^d^The mother liquor contained considerable amounts of product by TLC. ^e^From per-6-bromo-γ-CD. ^f^MPA: 3-mercaptopropionic acid; TEA: *N*,*N*,*N*-triethylamine; KO*t*-Bu: potassium *tert*-butoxide; KO*t*-Bu/MPA molar ratio ≈2.1:1.;^g^DDS: 1-dodecanethiol; DDS/KO*t*-Bu molar ratio 1:1.

Mechanochemical syntheses from 6^I^-O-monotosyl-β-CD usually require moderate reactant excesses and similar molar ratios to the solution method [24]. Despite the expectations, based on the monosubstituted case [24], the persubstitutions usually needed higher reagent/CD molar ratio. However, except the azide cases (**4a** and **4b**), the reagent/halogen molar ratios did not change such extent (Table 1). This can be explained satisfactorily by the complexation of the leaving group which might have high affinity to the CD cavity [3,25–26] preventing its departure from the reaction centres resulting in steric blocking. While the halogen → azide exchange required considerably larger halogen/reagent ratios than the solution reactions, sulfur nucleophiles showed a more favourable tendency and the reagent/halogen ratio was either roughly equal or slightly lower than for the monosubstitution. In the solution synthesis [6] of β-CD-per-6-dodecane thioether (**6**) the residual DMF increases the product solubility in methylene chloride and precipitation with MeOH removes the unreacted 1-dodecanethiol (DDS), whereas the absence of DMF, together with the strong complex formed between DDS and the product, meant that the product isolation was more difficult. The resulting crude mixture was only partially soluble in methylene chloride and MeOH precipitation gave a difficult-to-filter product, which still contained at least one mole of complexed DDS. This complexation resulted in not only technical difficulties, but also confounded the removal of impurities. The strong complex between the reagent and product also resulted in an impure product of the β-CD version of Sugammadex (**5b**). The reagent/halogen ratio was practically identical to the one used in the solution method of the originator [16] when mercaptopropionic acid reacted with the halogenated CD (entries 22, 23, and 26–28 in Table 1) despite the use of the considerably milder and safer base, potassium *tert*-butoxide (KO*t*-Bu).

Our first target was to investigate the reaction between a good nucleophile (sulfur) and per-halogenated CDs. The TU method is widely used in the synthesis of various thiols and thioester compounds [8,30] and the CD thiouronium salt intermediates are not isolated [8] despite their good crystallization properties, while the excess/residual TU and used solvents are removed upon the conversion to thiols only. However, the easy crystallization of these salts is a nice feature of these compounds as it can help to remove incompletely substituted compounds (defected structures) and unused reagent as the thiolate is less stable after their basic decomposition; thiols are easy to oxidize to disulfide under basic conditions. The TU reactions (**3a** and **3b**) in the ball mill gave yields that were usually lower that those given by the monosubstitution reactions [22]. The very low aqueous solubility of the CD halides led to the intact starting materials being completely lost during the work-up. The low TU/halogen ratio (entries 2, 7 and 8 in Table 1) gave incomplete reactions. Incompletely substituted derivatives were in the majority in the product, as can be seen in the multiple anomeric proton peaks in the ^1^H NMR spectra and the CH_2_-halogen signals in the 2D NMR spectra. The larger excess of TU gave the complete substitution of the halogen (entries 3, 4, 9–11 in Table 1), but the TU removal also caused difficulties on the hundred-milligram scale despite the crude products only contained the targeted derivative. The higher yields of the 10-times larger scale (entries 4 and 11in Table 1) clearly demonstrate the technical difficulty of purification and the effect of batch sizes on yields. It was found that a considerable portion of TU-CD remained in the mother liquor because of the relatively high amounts of used solvents and the solubility of thiouronium-CDs in EtOH. Solubility difficulties led to chromatographic purification attempts failing even in RP-18 silicagel columns, too. The yield increased considerably when bromine derivatives (entries 10 and 11 in Table 1) were used instead of their iodine analogues (entries 7 and 9 in Table 1). An identical TU/Br to TU/I ratio was used in case of **3b** and further reduced the yield (entry 8 in Table 1), demonstrating that the similar halogen/tosyl ratio used in the monotosylated case is not sufficient to lead to complete substitution. Owing to the lower reactivity of bromo-CD a higher TU/per-6-bromo-γ-CD ratio had to be used. The more ionic character of formed thiouronium bromide was seen not only in the higher yields, but also in the lower product content of the ethanolic mother liquor. Chloride salts are even more ionic compounds whose character can reduce the organic solvent solubility of thiouronium chlorides. It, however, seemed reasonable to test a per-6-chlorinated CD. Unfortunately, practically no reaction was found to occur and the reaction mixture contained only traces of incompletely substituted TUβ-CD (entry 5 in Table 1). A further increase in the TU/chlorine ratio seemed to be unreasonable, as removing the higher amount of TU brings back the purification problems. The higher TU ratio somehow also increased the yield in the β-CD version. Ten-fold scaling up of the experiments showed increasing yields (entries 4 and 11 in Table 1) but the small scale still prevented to reach the solution reaction outcome but proved our concept. Gram-scale preparations easily overcome the technical difficulties of TU removal found in the small scale, as it becomes clear in the scale-up experiments (entries 4 and 11 in Table 1).

Although the preparation of 6^I^-monoazido-6^I^-monodeoxy-β-CD [22] was very effective, the analogue reactions with per-6-substituted CDs (**4a** and **4b**) were less efficient. Either the reaction did not proceed at all or only partial substitution was achieved at low NaN_3_/halogen ratios, while only an increased NaN_3_ ratio afforded the complete substitution of the CH_2_–I groups, possibly because of the steric hindrance of the bulky sodium iodide. Iodine and metal iodides are preferred salts in various CD complexes [26,31]. Lack of solvents the diffusion/decomplexation of NaI from the cavity is slow. The higher amounts of sodium azide can exclude the formed NaI from the CD cavity in solid state. However, on a larger multigram scale the higher amount of used NaN_3_ can be easily regenerated because the products are very poorly soluble in water and NaI and NaN_3_ can be readily separated. In order to accelerate the decomposition of the assumed NaI/CD complex first water then 50% aq EtOH was used as wetting substance. Usually 50% EtOH is able to decompose most of the CD complexes and owing to the hydrogen bonding destruction increases the solubility of poorly soluble CD derivatives. Wetting the solid dispersion with water and 50% EtOH resulted in not only lower temperature but practically no reaction was experienced as confirmed by the lack of azide in the IR spectrum of the isolated solid. As it is previously found [22] applying a wetting substance the grinding temperature is always lower than the dry milling. It was assumed that the very low solubility of both per-halogeno and per-azido-CDs in water or 50% EtOH and complete dissolution of the sodium azide the milling energy was not enough to warm the reaction mixture to an appropriate value. The very low solubility of the CD azides created an additional disadvantage and so we discarded the use of these wetting solvents. Alternatively, a wetting substance, which is an equally poor solvent for the CD derivatives and NaI, 1-pentanol [22] was also tested. In this case, the final grinding temperature was also found lower, approximately 20 °C lower, than the dry milling and no azido-CD was found by IR spectroscopy in the isolated solid.

In the TU reactions, we found that the isolated yields depend on batch-size and we studied the effect of downsizing a solution reaction for the preparation of compound **4a** (entry 13 in Table 1). In the downsized reaction due to the low solubility of the NaN_3_ in DMF, an unreasonably high solvent/reagent ratio was necessary in the solution reaction and the yield was decreased due to the technical difficulties in the purification, e.g., relatively larger loss upon mass transfers or filtrations. No essential differences in reaction time were found despite all the necessary NaN_3_ being dissolved at the beginning of the reaction and only a slightly longer reaction time was required to the complete reaction despite the higher dilution. Although in this case the crude contained relatively less residual DMF but its complete removal was still impossible. The scaled up ball mill azidations (entries 17 and 20 in Table 1) resulted in little higher yields but the relatively small amounts still caused technical difficulties which is shown in the yield-increasing ratios of the β- and γ-CDs.

Finally, the smaller solution reaction scale resulted in considerably reduced yields, which were however close to those of the scaled-up mechanochemical method. The mechanochemical syntheses of **4a** and **4b** were successfully scaled up 10-fold (entries 17 and 20 in Table 1) with acceptable yields which provide the proof of concept for the ball-mill-assisted synthesis of per-6-azido-CDs.

Chloride salts have considerably less affinity to the β-CD cavity (≈1/6 of iodide [26]). Per-6-chlorinated CD were also tested in this reaction despite the fact that only traces of incompletely substituted azido-CDs and decomposition products were found in solution and that the reaction mixture composition was similar to the solution composition (entries 14 and 15 in Table 1).

In conclusion, it appears that the advantage of mechanochemistry is restricted to the elimination of high boiling solvents in the azide cases (entries 16, 17, 19, and 20 in Table 1) which provides significant, if not dramatic, improvements in the synthesis. However, it is also true that the price of sodium azide is considerable lower than the costs of the utilization and regeneration of polar aprotic organic solvents, including environmental impact.

The TU reactions (compounds **3a** and **3b**) demonstrated that a good nucleophile, such as sulfur, could be effectively used in the preparation of useful per-6-thio-CD derivatives. While simple CD-6-thiols are still at the scientific stage, the sodium salt of octakis(6-deoxy-6-*S*-(3-mercapto)propionyl)-γ-CD has been slowly becoming an important surgical aid. The solution phase reaction is a dangerous process due to the use of sodium hydride. The relatively large number of possible side-reactions in solution brings further challenges to production. The efficient and green synthesis of Sugammadex is an exciting task. The comparable masses of reaction components meant that reaction assembly had less influence on the yields in the preparation of 3-mercaptopropionyl derivatives (**5a** and **5b**). While an inert wetting component (1-pentanol) was needed in the monosubstituted analogue [22], no such component was necessary in the per-6-substitution and the order in which the reagents were added had no effect on the yield. Purification and conversion to the pharmaceutically active form became very simple, as the protonated form is poorly soluble in water: the precipitate formed upon acidification by HCl, was filtered and the solid was re-dissolved in the equivalent amount of base. But, it is also true, that complex formation between the MPA and the product, particularly in case of the β-CD version, pointed out that pharmaceutical grade preparations need more fine tuning not only in the reaction but in the purification method, as well. Although, Bridion^®^ is a sodium salt of the MPAγ-CD but in order to avoid the overdosing of base on the small preparation scale in our experiments aqueous ammonia was chosen as base and acetone was needed to get less deficit in the precipitates. Considerable losses during filtrations were found in the small-scale cases, which significantly reduced the yields because of the inevitably used diluted solutions.

The ten-fold scale up (entry 28 in Table 1) further simplified the product isolation because no acetone was necessary to precipitate the product from the acidic solution, which could be isolated by filtration, and then it was immediately converted to the ammonium salt. Although, some cases the further simplified work-up resulted in somehow lower yields because of the redissolution of the protonated product during the filtration, the complete elimination of an organic solvent could be achieved. Yields can be improved further at even larger scales.

The encouraging results with MPA led us to an attempt to simplify the synthesis of an intermediate of a promising candidate for siRNA delivery. The solution phase synthesis of heptakis(6-deoxy-6-*S*-alkyl)-β-CDs is typical for the syntheses of intermediates of such compounds, however, their high lipophilicity means that isolation is not a technically trivial task.

Scale up of the 1-dodecanethiol mechanochemical reaction (synthesis of compound **6**, entry 31 in Table 1) was not performed because the batch size was in the range of our solution reaction. Mechanochemical synthesis was much more efficient than conventional solution methods. The final temperature was considerably lower (entry 31 vs 30 in Table 1) and the reaction time was dramatically shorter. The lack of residual DMF somehow changed the solubility behaviour of the product and affected the purification to some extent. This green approach resulted in better yields and purity despite the lower molar ratio of the reagent DDS.

The persubstituted species showed less variability in their susceptibility to the heat effect of the ball milling than their monosubstituted analogues. The temperatures inside the jar were considerably lower when one of the reactants was liquid or became liquid during the reaction (entries 22, 23, 26–28 and 31 in Table 1). In all cases, the temperature-time curves showed a saturation-like trend (see Supporting Information File 1 for details) and processes were generally in the 70–90 °C range at the end of the reaction, which is not essentially different from the monosubstituted case [18].

Although limits caused by the intrinsic complexation properties of CDs sometimes affected the reaction rate, a solvent-free synthetic method may simplify the purification of compounds **5** and **6**.

## Conclusion

Syntheses of per-6-substituted CD derivatives can be effectively carried out in a ball mill under solvent-free conditions. In many cases, ball mill preparations display a positive balance in cost-benefit analyses. Wet grinding for the preparation of per-6-azido-CDs, using solubilizing and non-solubilizing solvents, showed practically no reaction in the planetary ball mill. Important intermediates and final products of per-6-amino- and per-6-thio-CDs can be prepared without polar aprotic solvents, by which the byproduct formation and difficult-to-remove impurities can be eliminated. The lack of solvents in the examples described herein simplified the isolation and purification processes. Our basic aim was to proof the concept and although the purifications were not optimized the prepared compounds were enough pure to record correct NMR spectra to identify the substitution location and completeness.

Although in the monosubstituted case usually less reagent/leaving group molar ratios were found [22], in the majority of per-substitutions higher reagent/CD molar ratio was needed but the reagent/halogen ratio not always changed dramatically. As may be expected, the sulfur nucleophiles resulted in considerably better or almost equal yields as compared to the conventional solution methods. A potential drawback of the method lies in the fact that the lack of highly solubilizing organic solvents can cause difficulties in the primary stage of purification.

## Experimental

Full synthetic details and spectroscopic data are reported in Supporting Information File 1.

The syntheses of per-6-iodo-β- and -γ-CD, I_7_β-CD (**2a**) and I_8_γ-CD (**2b**), were performed using a small modification to the known method [13], from freshly dried CDs on a 0.01 mol scale with triphenylphosphine and iodine in DMF. Per-6-bromo-γ-CD (**2b’**) was prepared in *N*-methylpyrrolidone by the same method using bromine. Per-6-chloro-β-CD (**2a'**) was synthesized in a similar manner to per-6-iodo-CDs using *p*-toluenesulfonyl chloride.

### General conditions for the solution reactions

Syntheses of compounds **3a**, **3b**, **4a**, **4b**, **5b** and **6** were carried out in DMF at 60–100 °C. For **5b**, triethylamine was used as base, while KO*t*-Bu was used for **6**.

### General procedures for the high-energy ball milling reactions

Syntheses of compounds **3a**, **3b**, **4a**, **4b**, **5b** and **6** were carried out in a Retsch PM100 High Speed Planetary Ball Mill. 1500 steel balls of 1 mm diameter (44.94 g) and 50 steel balls of 5 mm diameter (25.54 g, total weight of balls = 70.5 g, V = 15 mL), were placed in a stainless steel jar of 50 mL, with a sun wheel speed of 650 min^–1^ for 120 min, weight = 780 g (jar, cap, and balls). Temperatures were measured using a Lafayette TRI-88 no-contact thermometer, built-in laser pointer, with ±2 °C reading accuracy, distance to spot size = 8:1, measuring distance 18–23 cm. The measurement matrix formed "a five on a die", two measurements were made at each point and the values were averaged.

## Supporting Information

File 1 Details of synthetic procedures and characterization of prepared compounds.
