# Supplementary material for: Efficient mechanochemical synthesis of regioselective persubstituted cyclodextrins
Source: Beilstein J Org Chem. 2016 Nov 10;12:2364–71. doi: 10.3762/bjoc.12.230 (PMC5238617; doi:10.3762/bjoc.12.230)
Supplement: File 1 — Details of synthetic procedures and characterization of prepared compounds. [file Beilstein_J_Org_Chem-12-2364-s001.pdf]

Supporting Information
for

Efficient mechanochemical synthesis of regioselective persubstituted cyclodextrins

Laszlo Jicsinszky*, Marina Caporaso, Katia Martina, Emanuela Calcio Gaudino and Giancarlo Cravotto*

Department of Drug Science and Technology and NIS - Centre for Nanostructured Interfaces and Surfaces, University of Turin, Via P. Giuria 9, 10125 Turin (Italy);

Email: Laszlo Jicsinszky - ljicsinszky@gmail.com; Giancarlo Cravotto - giancarlo.cravotto@unito.it

*Corresponding author

Experimental details and characterization of the prepared compounds

This *Supporting Information* file contains the experimental details of the syntheses and IR/NMR and MS characterization (**3a**, **3b**, **5a**, **5b** only) of the prepared compounds.

Table of Contents

Reaction Scheme S3
Experimental Details S3
Instrumentation and reagents S3
Syntheses in solutions S4
High Energy Ball Milling reactions S7
IR spectra
Table S4 Characteristic IR absorption bands (cm^{-1}) of the prepared compounds S14

Fig. S5 IR spectrum of heptakis(6-deoxy-6-iodo)-βCD **2a** S15
Fig. S6 IR spectrum of heptakis(6-deoxy-6-chloro)-βCD **2a'** S15
Fig. S7 IR spectrum of octakis(6-deoxy-6-iodo)-γCD **2b** S16
Fig. S8 IR spectrum of octakis(6-bromo-6-deoxy)-γCD **2b'** S16
Fig. S9 IR spectrum of heptakis(6-deoxy-6-S-thiuronium)-βCD iodide **3a** S17
Fig. S10 IR spectrum of octakis(6-deoxy-6-S-thiuronium)-γCD iodide **3b** S17
Fig. S11 IR spectrum of octakis(6-deoxy-6-S-thiuronium)-γCD bromide **3b'** S18
Fig. S12 IR spectrum of heptakis(6-azido-6-deoxy)-βCD **4a** S18
Fig. S13 IR spectrum of octakis(6-azido-6-deoxy)-γCD **4b** S19
Fig. S14 IR spectrum of heptakis(6-deoxy-6-S-(3-mercapto)propionyl))-βCD NH_4^+ **5a** S19
Fig. S15 IR spectrum of octakis(6-deoxy-6-S-(3-mercapto)propionyl))-γCD NH_4^+ **5b** S20
Fig. S16 IR spectrum of heptakis(6-deoxy-6-S-(1-dodecylthio))-βCD **6** S20

MS spectra
Fig. S17 ESI-MS spectrum of heptakis(6-deoxy-6-S-thioureido)-βCD **3a** S21
Fig. S18 ESI-MS spectrum of octakis(6-deoxy-6-S-thioureido)-γCD **3b** S21
Fig. S19 ESI-MS spectrum of heptakis(6-deoxy-6-S-(3-mercapto)propionyl)-βCD **5a** S21
Fig. S20 ESI-MS spectrum of octakis(6-deoxy-6-S-(3-mercapto)propionyl)-γCD **5b** S21

NMR spectra
Table S5 Proton NMR assignment (ppm) of compounds (from 1H and HSQC spectra) S22
Table S6 Carbon NMR assignment (ppm) of compounds (from HSQC spectra) S23

Fig. S21 1H-NMR spectrum of heptakis(6-deoxy-6-iodo)-βCD **2a** S24
Fig. S22 HSQC-NMR spectrum of heptakis(6-deoxy-6-iodo)-βCD **2a** S24
Fig. S24 1H-NMR spectrum of heptakis(6-deoxy-6-chloro)-βCD **2a'** S25
Fig. S25 HSQC-NMR spectrum of heptakis(6-deoxy-6-chloro)-βCD **2a'** S25
Fig. S25 1H-NMR spectrum of octakis(6-deoxy-6-iodo)-γCD **2b** S26
Fig. S26 HSQC-NMR spectrum of octakis(6-deoxy-6-iodo)-γCD **2b** S26
Fig. S27 1H-NMR spectrum of octakis(6-bromo-6-deoxy)-γCD **2b**' S26
Fig. S28 HSQC-NMR spectrum of octakis(6-bromo-6-deoxy)-γCD **2b**' S27
Fig. S29 1H-NMR spectrum of heptakis(6-deoxy-6-thioureido)-βCD iodide **3a**, thiourea/I ratio=3.5 S28
Fig. S30 HSQC-NMR spectrum of heptakis(6-deoxy-6-thioureido)-βCD iodide **3a**, thiourea/I ratio=3.5 S28
Fig. S31 1H-NMR spectrum of octakis(6-deoxy-6-thioureido)-γCD iodide **3b**, thiourea/I ratio=1.5 S29
Fig. S32 HSQC-NMR spectrum of octakis(6-deoxy-6-thioureido)-γCD **3b**, thiourea/I ratio=1.5 S29
Fig. S33 1H-NMR spectrum of octakis(6-deoxy-6-thioureido)-γCD iodide **3b**, thiourea/I ratio=3.5 S30
Fig. S34 HSQC-NMR spectrum of octakis(6-deoxy-6-thioureido)-γCD iodide **3b**, thiourea/I ratio=3.5 S30
Fig. S35 1H-NMR spectrum of octakis(6-deoxy-6-thioureido)-γCD bromide **3b'**, thiourea/I ratio=3.5 S31
Fig. S36 HSQC-NMR spectrum of octakis(6-deoxy-6-thioureido)-γCD bromide **3b'**, thiourea/I ratio=3.5 S31
Fig. S37 1H-NMR spectrum of heptakis(6-azido-6-deoxy)-βCD **4a** S32
Fig. S38 HSQC-NMR spectrum of heptakis(6-azido-6-deoxy)-βCD **4a** S32
Fig. S39 1H-NMR spectrum of octakis(6-azido-6-deoxy)-γCD **4b** S33
Fig. S40 HSQC-NMR spectrum of octakis(6-azido-6-deoxy)-γCD **4b** S33
Fig. S41 1H-NMR spectrum of heptakis(6-deoxy-6-S-(3-mercapto)propionyl)-βCD $NH4^+$ **5a** S34
Fig. S42 HSQC-NMR spectrum of heptakis(6-deoxy-6-S-(3-mercapto)propionyl)-βCD $NH4^+$ **5a** S34
Fig. S43 1H-NMR spectrum of octakis(6-deoxy-6-S-(3-mercapto)propionyl)-γCD $NH4^+$ **5b** S35
Fig. S44 HSQC-NMR spectrum of octakis(6-deoxy-6-S-(3-mercapto)propionyl)-γCD $NH4^+$ **5b** S35
Fig. S45 1H-NMR spectrum of heptakis(6-deoxy-6-S-(1-dodecylthio)-βCD **6** S36
Fig. S46 HSQC-NMR spectrum of heptakis(6-deoxy-6-S-(1-dodecylthio)-βCD **6** S36

References S37

Reaction Scheme

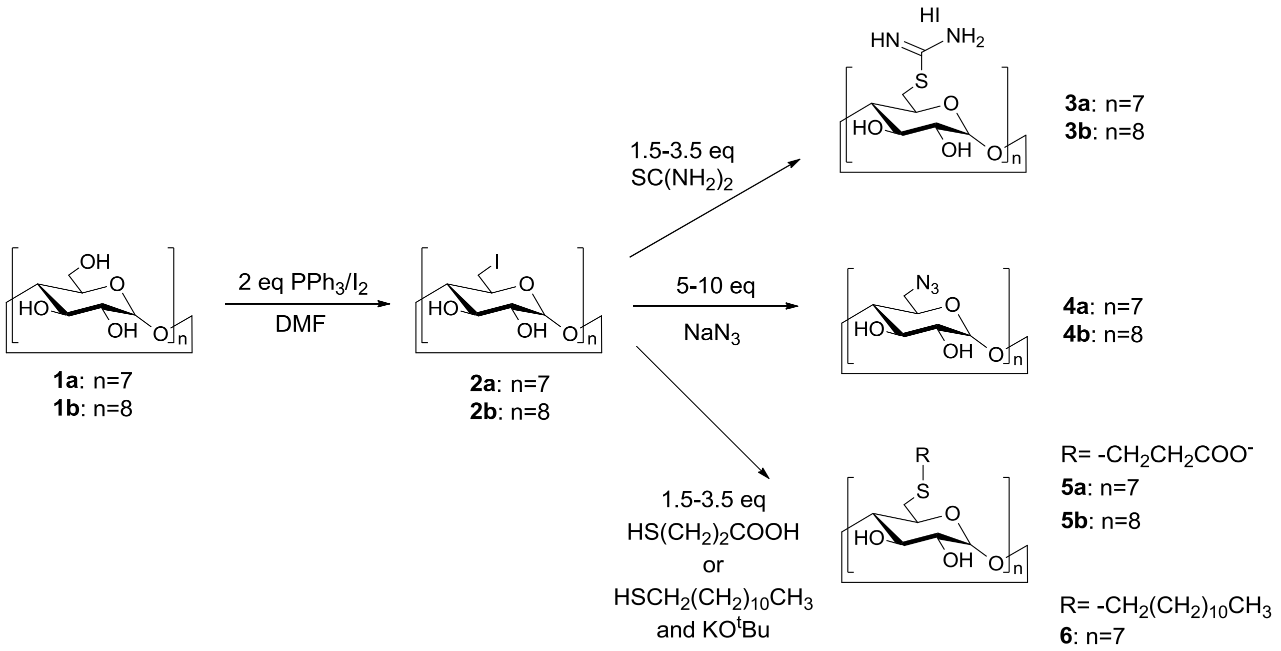

For **2a’** TsCl, for **2b’** Br2 and *N*-methylpirrolidone was used instead of I2 and DMF

Experimental Details

Instrumentation and reagents

Ball mill: Retsch PM100 High Speed Planetary Ball Mill, 1500 steel balls of 1 mm diameter (44.94 g) and 50 steel balls of 5 mm diameter (25.54 g, total weight of balls=70.5 g, V=15 mL), in a jar of 50 ml, sun wheel speed 650 min-1 for 120 min, weight = 780 g (jar, cap, and balls). Reagents other than cyclodextrins and solvents were purchased from AlfaAesar and Sigma-Aldrich. Cyclodextrins were the generous gift of Roquette, France

Thermometer: Lafayette TRI-88 no-contact thermometer, built-in laser pointer, with ± 2 °C reading accuracy, distance to spot size = 8:1, measuring distance 18-23 cm. The measurement matrix formed "a five on a die", two measurements were made at each point and the values were averaged.

NMR. 1H and HSQC-DEPT spectra were recorded at 20 °C with a Bruker Avance 300 MH at 300.13 MHz and 75.47 MHz, respectively. The 1H NMR spectra were obtained by using standard pulse programs from Bruker library using 16 K data points of 64 transients in a 4496.40 Hz spectral width.

The HSQC-DEPT experiments were acquired by pulse sequence *hsqcedtpg* from Bruker library using 128x64 data points of 32 transients. **2a'** NMR analysis were performed on a Varian System 600 MHz spectrometer. Solvent signals used for referencing. NMR data were processed with ACD/NMR Processor Academic Edition Release 12.00 product version 12.01 (Build 39104), Advanced Chemistry Development Inc. (www.acdlabs.com).

IR spectra were recorded on a PerkinElmer 1005 reflection IR spectrometer in KBr matrix, except **2a'** which was recorded on a Nicolet IR spectrometer.

Melting points were measured with Büchi 545 and values are uncorrected.

TLC: TLC experiments used Merck 5554 Silicagel 60, saturated chamber, regular running distance was 7 cm. Samples were dissolved in DMF or water at 10% concentration.

Syntheses in solutions

Synthesis of per-6-halogenated β- and γCD derivatives 2a, 2a', 2b, 2b':

The syntheses of per-6-iodo-β- and -γ-CD, I_7βCD, (**2a**) and I_8γCD (**2b**), were performed using a small modification to the known method [1], from freshly dried βCD (**1a**) and γCD (**1b**) on a 0.01 mol scale with triphenylphosphine (TPP, 0.15 and 0.17 mol, respectively) and iodine (0.15 and 0.17 mol, respectively) in DMF (115-130 mL). Yields: **2a** 16.4 g, 86% and **2b** 17.1 g, 79%. Per-6-bromo-γCD (**2b'**) was prepared in *N*-methylpyrrolidone by the same method using bromine. The prepared compounds contained < 1 % TPP and approx. 1 mol of DMF by IR/NMR.

Per-6-chloro-βCD (**2a'**) was synthesized as per-6-iodo-CDs using p-toluenesulfonyl chloride [2]. The prepared compounds contained < 0.3 % TPP, < 5 % p-toluenesulfonic acid related compounds, and approx. 1 mol of DMF.

2a R_f=0.61-0.64 (max. 25 µg; 1,4-dioxane/cc. NH_3-H_2O=10:7); m.p. 205-208 °C [dec]; ^{1}H NMR (300 MHz, DMSO-d_6, 20°C): δ= 5.03 (d, $^2J_{1,2}$(H,H)=2.4 Hz, 7H; H-1), 3.33-3.50 (m, 7H; H-2), 3.56-3.73 (m, 7H; H-3), 3.25-3.41 (m, 7H; H-4), 3.65-3.76 (m, 7H; H-5), 3.77-3.92, 3.40-3.55 ppm (m, 14H; H-6a,b); ^{13}C NMR (from HSQC, 75 MHz, DMSO-d_6, 25°C): δ=103.23 (C-1), 73.14 (C-2), 72.53 (C-3), 87.11 (C-4), 73.75 (C-5), 9.68, 9.91 ppm (C-6), IR (KBr), cm^{-1}: 2912-3032 (C-H), 1658 (O-C-O).

2a' R_f=0.73-0.77 (max. 25 µg; 1,4-dioxane/cc. NH_3-H_2O=10:7); m.p. 207-210 °C [dec]; ^{1}H NMR (600 MHz, DMSO-d_6, 25°C): δ= 4.96 (d, $^2J_{1,2}$(H,H)=3.4 Hz, 7H; H-1), 3.32-3.46 (m, 7H; H-2), 3.55-3.69 (m, 7H; H-3), 3.29-3.46 (m, 7H; H-4), 3.76-3.91 (m, 7H; H-5), 4.02-4.13, 3.73-3.85 ppm (m, 14H; H-6a,b); ^{13}C NMR (from HSQC, 100 MHz, DMSO-d_6, 25°C): δ=101.94 (C-1), 71.85 (C-2), 72.37 (C-3), 83.47 (C-4), 71.03 (C-5), 44.73, 44.78 ppm (C-6), IR (KBr), cm^{-1}: 2884-2925 (C-H), 1675 (O-C-O).

2b R_f=0.69-0.73 (max. 25 µg; 1,4-dioxane/cc. NH_3-H_2O=10:7); m.p. 215-216 °C [dec]; ^{1}H NMR (300 MHz, DMSO-d_6, 20°C): δ= 5.07 (d, $^2J_{1,2}$(H,H)=3.3 Hz, 8H; H-1), 3.35-3.48 (m, 8H; H-2), 3.57-3.72 (m, 8H; H-3), 3.25-3.38 (m, 8H; H-4), 3.57-3.72 (m, 8H; H-5), 3.86,3.45 ppm (m, 16H; H-6a,b); ^{13}C NMR (from HSQC, 75 MHz, DMSO-d_6, 25°C): δ=102.22 (C-1), 72.8 (C-2), 71.99 (C-3), 85.73 (C-4), 71.99 (C-5), 9.75, 9.87 ppm (C-6), IR (KBr), cm^{-1}: 2905-3032 (C-H), 1658 (O-C-O).

2b' R_f=0.72-0.75 (max. 50 µg; 1,4-dioxane/cc. NH_3-H_2O=10:7); m.p. 235-237 °C [dec]; ^{1}H NMR (300 MHz, DMSO-d_6, 20°C): δ= 5.06 (d, $^2J_{1,2}$(H,H)=3.3 Hz, 8H; H-1), 3.26-3.53 (m, 8H; H-2), 3.57-3.75 (m, 8H; H-3), 3.22-3.58 (m, 8H; H-4), 3.72-3.97 (m, 8H; H-5), 3.85-4.17, 3.52-3.85 ppm (m, 16H; H-6a,b); ^{13}C NMR (from HSQC, 75 MHz, DMSO-d_6, 25°C): δ=102.11 (C-1), 72.77 (C-2), 72.47 (C-3), 84.47 (C-4), 71.82 (C-5), 35.22 ppm (C-6), IR (KBr), cm^{-1}: 2832-3038 (C-H), 1654 (O-C-O).

Preparation of 3a/b-6 using the classic method:

3a and **3b**. Per-6-S-thiouronium-CDs were prepared according to the known method [3].

4a and **4b**. The known method [4] for the preparation of per-6-azido-CDs (β: 9.5 g, γ: 10.9 g, 0.0075 mol) was modified considerably: the required amount of sodium azide (β: 2.8 g, 0.044 mol; γ: 3.3 g, 0.050 mol; 1.25 eq. to CH_2-I) was divided into 5 equal parts, the first portion was dissolved in DMF (150 mL) together with freshly dried per-6-halogeno-CDs and the solution was heated to 100 °C in 30-45 min. The subsequent NaN_3 portions were added at 50 °C, 75 °C, 85 °C and 95 °C at a rate that would keep the reaction mixture homogenous. The reaction mixture was stirred for a further 4 h at 100-105 °C once the additions were completed. After this time, the reaction mixture was cooled to around 40-45 °C and the majority of the DMF was removed by evaporation under reduced pressure

at 45-47 °C. (The obtained liquid turned solid below ~ 40 °C.) The warm residue was poured onto r.t. MeOH (150 mL) under ultrasonication and allowed to crystallize overnight. The solid was filtered and washed with MeOH (3x10 mL) and dried at 75-80 °C. Yields, **4a**: 5.9 g, 90.1%; **4b**: 6.3 g, 84.2%. Preparation of **4a** was repeated in 0.5 mmol scale (0.95 g, 0.0005 mol) using identical molar ratio with dissolved NaN_3 (0.29 g, 0.0044 mol) in DMF solution (50 ml), stirring for 5 h at 100-105 °C. Workup as before, yield: 0.5 g, 76.3%.

5a and **5b**. Literature data [5,6] were used and **5b** was prepared *via* the modification of the known method when trimethylamine was used as base instead of NaH.

6. Synthesis of heptakis(6-deoxy-6-S-(1-dodecylthio))-βCD, due to the lacking detailed literature batch data [7,8], was carried out analogously of the known method for heptakis(6-deoxy-6-S-(1-hexylthio))-βCD [9] on 0.5 mmol scale The work-up needed some modifications due to technical difficulties in filtration. Yield: 1.0 g, 82.5%.

High Energy Ball Milling reactions

Thioureido (TU) CDs 3a and **3b**.

Air-dry per-6-halogeno CD were mixed in the jar with a spatula and then ball milled for 120 min. The temperature was checked after 30, 60, 90 and 120 min using the infra thermometer.

The ground material was dissolved in water (15 mL) and the equipment washed with water (3x5 mL) and freeze-dried. The obtained solid was suspended in abs EtOH (10 ml), filtered and washed in abs EtOH (4x3 mL) and then with acetone (5x3 mL). The obtained solid was dissolved in water, filtered through charcoal and the filtrate was freeze-dried. TLC showed 30-40% products in the mother liquor after the organic solvents removal.

Scaled-up preparation of 3a and 3b' Ten-fold scale-up of ball milling reactions were performed using identical conditions with the original scale. The work-up used identical amounts of solvents as described in the previous paragraph. TLC showed 15-30% products in the mother liquor after the organic solvents removal.

3a, *entry 4** R_f=0.43-0.47 (max. 50 µg; 1,4-dioxane/cc. NH_3-H_2O=10:7); ESI-MS (neutralized with NaOH, m/z): 1542.26 $[M+H]^+$, 371.34 $[M-3(CN_2H_3)+HCOONa+4H]^{4+}$, 236.32 $[M+Na_2CO_3+7H]^{7+}$.

3b *entry 9* R_f=0.39-0.44 and **3b'** *entry 11* R_f=0.38-0.42 (max. 50 µg; 1,4-dioxane/cc. NH_3-H_2O=10:7); ESI-MS (after neutralization, m/z): 595.78 $[M+2H+Na]^{3+}$, 441.33 $[M+4H]^{4+}$, 353.44 $[M+5H]^{5+}$. IR and NMR assignments are in Table S 4, Table S 5, and Table S 6

* Entry numbers are corresponding to the entry numbers in **Table 1** of the main text.

Table S 1 ***Amounts of reagents and yields (scaled up experiments are in italics):***

	CH_2-**X**	Compnd.	Entries in Table 1	CD [g (mol)]	TU/CH_2–X molar ratio	Balls/ substance mass ratio	TU [g, (mol)]	Yield [g (%)]
βCD	**I**	**3a**	2	0.190 (0.00010)	1.5	230	0.116 (0.00105)	0.03 (12.3)
	**I**	**3a**	3	0.190 (0.00010)	3.5	153	0.270 (0.00245)	0.06 (24.6)
	I	***3a***	*4*	*1.904 (0.00100)*	*3.5*	*15*	*2.700 (0.02450)*	*1.48 (61)*
	**Cl**	**3a'**	5	0.126 (0.00010)	3.5	178	0.270 (0.00245)	traces
γCD	**I**	**3b**	7	0.218 g, (0.00010)	1.5	248	0.066 (0.00120)	0.04 (14.4)
	**Br**	**3b'**	8	0.090 g, (0.00005)	1.5	451	0.066 (0.00060)	0.022 (9.1)
	**I**	**3b**	9	0.109 g, (0.00005)	3.5	268	0.154 (0.00140)	0.046 (33.0)
	**Br**	**3b'**	10	0.090 g, (0.00005)	3.5	288	0.154 (0.00140)	0.047 (39.0)
	Br	***3b'***	*11*	*0.900 g, (0.00050)*	*3.5*	*29*	*1.543 (0.01400)*	*0.69 (57.3)*

βCD

derivatives

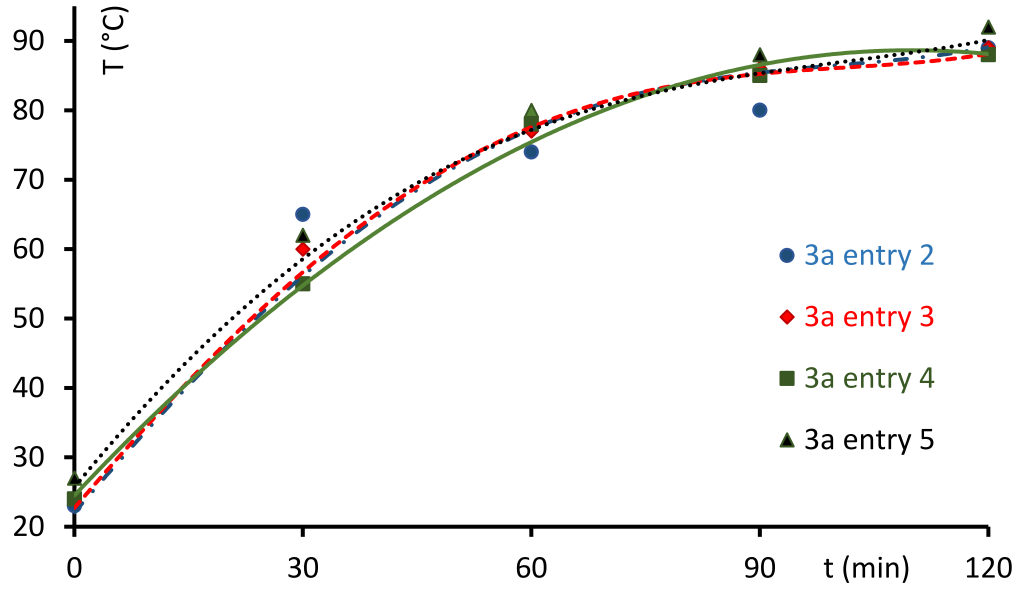

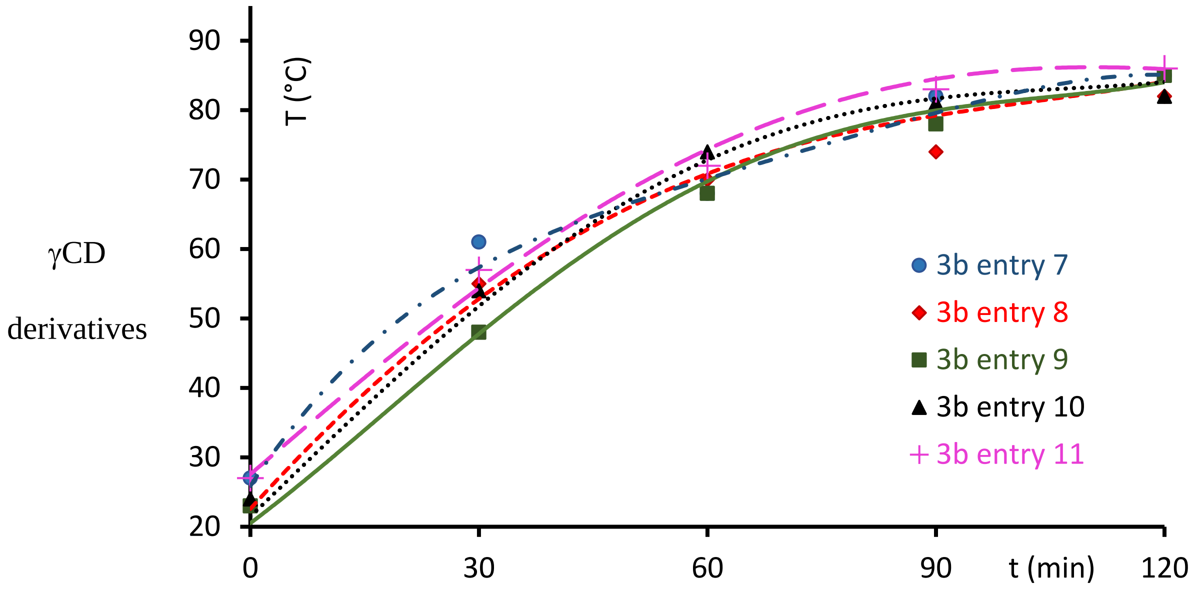

Fig. S 1 Temperature-ball milling time curves of thiourea/per-6-halogenated CDs

Per-6-azido CDs 4a and 4b. The air-dry per-6-iodo-CD were mixed in the jar with a spatula and then ball milled for 120 min. The temperature was checked after 30, 60, 90 and 120 min using the infra thermometer. The ground material was suspended in water (30 mL) and the equipment was washed with water (3x10 mL). The filtered material was dissolved in DMF (0.5 ml) and precipitated with methanol (6 ml) and the solid was dried at around 70 °C, under reduced pressure, in the presence of P_2O_5 and KOH.

Scaled-up preparation of 4a and 4b (entries 17 and 20, respectively): The air-dry per-6-iodo-CD were mixed in the jar with a spatula and then ball milled for 120 min. The work-up used double amounts of organic solvents than as it is described in the previous paragraph.

4a *entry 17* R_f=0.59-0.62 (max. 75 µg; presumably as I^- complex, 1,4-dioxane/cc. NH_3-H_2O=10:7).

4b *entry 20* R_f=0.68-0.72 (max. 75 µg; presumably as I^- complex, 1,4-dioxane/cc. NH_3-H_2O=10:7).

IR and NMR assignments are in Table S 4, Table S 5, and Table S 6

Table S 2 ***Amounts of reagents and yields*** **(scaled up experiments are italics)*:***

	CH_2-**X**	Compnd.	Entries in Table 1	CD [g (mol)]	NaN_3/CH_2–X molar ratio	Balls/ substance mass ratio	NaN_3 [g, (mol)]	Yield [g (%)]
βCD	**Cl**	**4a'**	15	0.126 (0.00010)	5	199	0.228 (0.0035)	traces
	**I**	**4a**	16	0.190 (0.00010)	5	169	0.228 (0.0035)	0.06 (68.7)
	I	***4a***	*17*	*0.952 (0.00050)*	*5*	*34*	*1.138 (0.0175)*	*1.48 (71.8)*
	**I**[1]	**4a**	-	0.190 (0.00010)	5	169	0.228 (0.0035)	traces
	**I**[2]	**4a**	-	0.190 (0.00010)	5	169	0.228 (0.0035)	traces
	**I**[3]	**4a**	-	0.190 (0.00010)	5	169	0.228 (0.0035)	traces
γCD	**I**	**4b**	19	0.109 g, (0.00005)	10	191	0.260 (0.0040)	0.05 (66.8)
	I	***4b***	*20*	*1.088 g, (0.00050)*	*10*	*19*	*2.600 (0.0400)*	*0.532 (71.1)*

[1] added 100 µl 1:1 (v/v) EtOH water before milling
[2] added 50 µl water before milling
[3] added 50 µl 1-pentanol before milling

βCD

derivatives

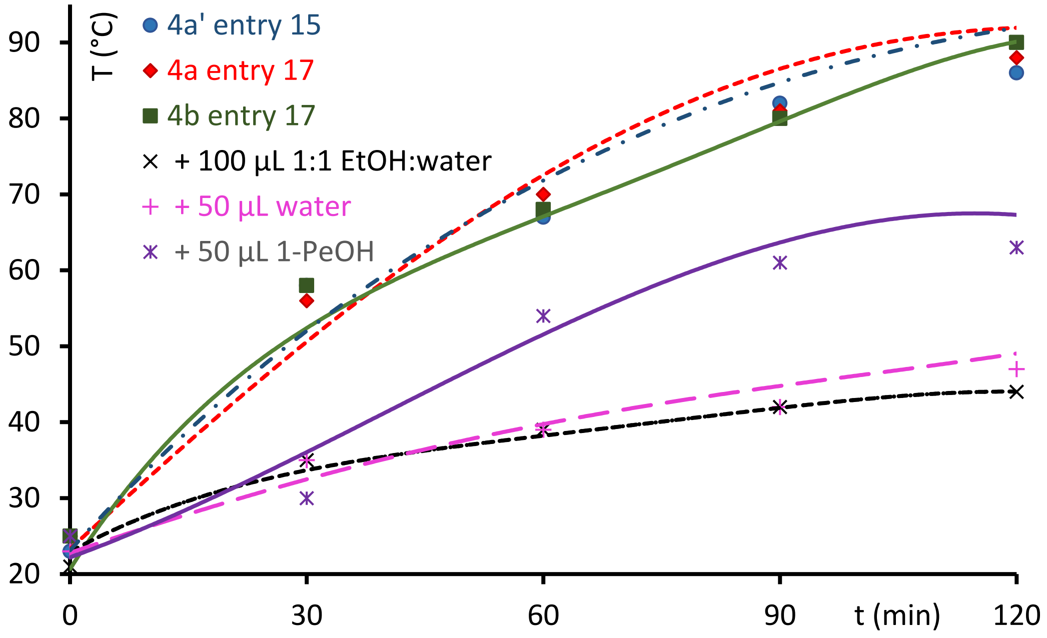

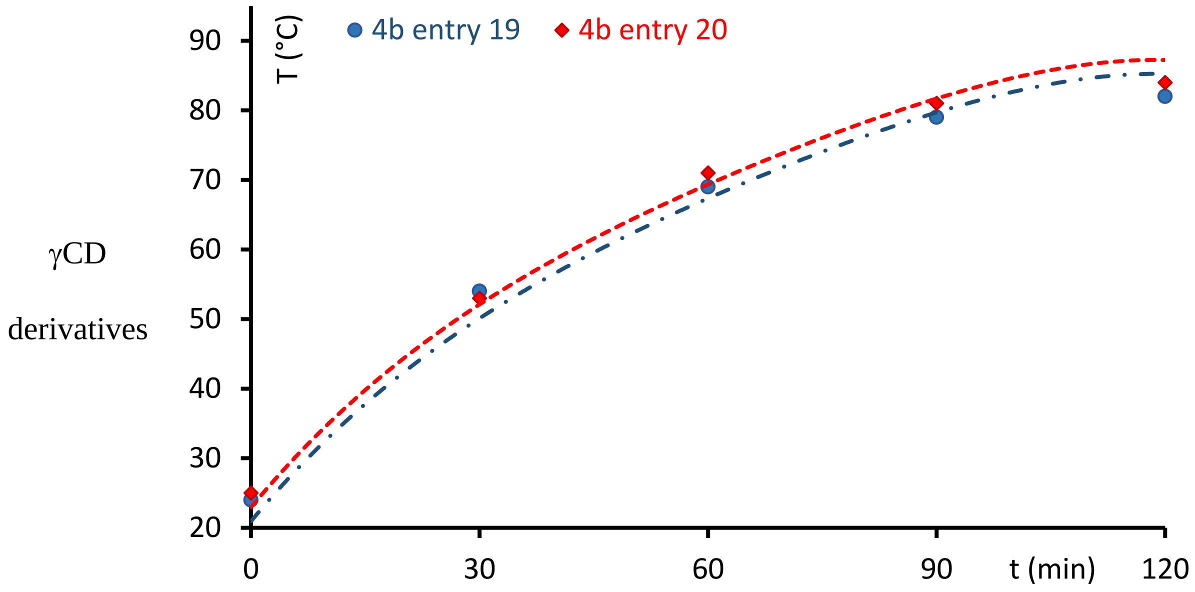

Fig. S 2 Temperature-ball milling time curves of NaN_3/per-6-halogenated CDs

Per-6-S-carboxyethyl CDs 5a and 5b. MPA and potassium t-butoxide (double molar amount of MPA) were added to the jar, the formed solid was cracked using a spatula, and air-dry per-6-iodo-CD was added, then mixed with a spatula in the jar, balls were added and ball milling was carried out for 120 min. The temperature was checked after 30, 60, 90 and 120 min using the infra thermometer. The ground material was dissolved in water (15 mL) and the equipment washed with water (3x5 mL) filtered and then freeze-dried. The solid was dissolved in water (1 mL), 1 M HCl was added (2 mL) and the product was precipitated in acetone (30 mL). The solid was filtered, washed with acetone and then dissolved in ammonia containing water (5 mL), filtered through charcoal and then the filtrate was freeze-dried. No CH_2-I signal was found in the HSQC spectrum and products contained some of complexed MPA.

The γCD reaction was repeated, with altered reagent addition sequence and practically identical yields were found, data are not inserted to the tables.

Scaled-up preparation of 5a and 5b (entries 23 and 28, respectively). Ten-fold scale-up of ball milling reactions carried out as described in the previous paragraph but the isolation method was a little different: the larger amount allowed the filtration of the protonated products without addition of acetone. The freeze-dried crude was dissolved in 5 mL water (pH ~7.5-8.0) and then the pH was adjusted <2 with 2 M HCl when the product precipitated. The solid was filtered, washed 3x2 mL

water. The solid was dissolved aqueous ammonia solution, filtered and washed 3*1 mL water then freeze dried.

5a *entry 23* R_f=0.02-0.05 (max. 25 µg; 1,4-dioxane/cc. NH_3-H_2O=10:7); ESI-MS (m/z): 362.85 $[M+HCOONa-5H]^{5-}$, 360.83 $[M+NaCl-5H]^{5-}$, 323.23 $[M+HCOONa+MPANa-5H]^{5-}$.

5b *entry 28* R_f=0.02-0.05 (max. 25 µg; 1,4-dioxane/cc. NH_3-H_2O=10:7); ESI-MS (m/z): 444.85 $[M+5HCOOH-5H]^{5-}$, 420.95 $[M+MPA-5H]^{5-}$, 363.22 $[M+4HCOOH-6H]^{6-}$, 317.07 $[M+5HCOOH-7H]^{7-}$, 277.09 $[M+2(HCOOH+HCOONa)-8H]^{8-}$.

IR and NMR assignments are in Table S 4, Table S 5, and Table S 6

Table S 3 ***Amounts of reagents and yields*** **(scaled up experiments are italics)*:***

	CH_2-**X**	Compnd.	Entries in Table 1	CD [g (mol)]	MPA/CH_2–X molar ratio	Balls/ substance mass ratio	MPA [g, (mol)]	Yield [g (%)]
βCD	**I**	**5a**	22	0.190 (0.00010)	1.43	124	0.122 (0.00115)[1]	0.16 (85.5)
	I	***5a***	*23*	*1.900 (0.00100)*	*1.5*	*12*	*1.274 (0.01200)*[2]	*1.33 (71.1)*
γCD	**I**	**5b**	26	0.218 g, (0.00010)	1.25	129	0.106 (0.0010)[3]	0.087 (81.4)
	**Br**	**5b'**	27	0.090 g, (0.00005)	1.5	243	0.064 (0.0006)[4]	0.092 (86.0)
	Br	***5b'***	*28*	*0.900 g, (0.00050)*	*1.5*	*24.4*	*0.637 (0.0060)*[5]	*0.774 (72.4)*

[1] KO^{t}Bu: 0.258 g, 0.0023 mol; [2] KO^{t}Bu: 2.693 g, 0.0240 mol; [3] KO^{t}Bu: 0.224 g, 0.0020 mol;
[4] KO^{t}Bu: 0.135 g, 0.0012 mol; [5] KO^{t}Bu: 1.347 g, 0.0120 mol

βCD

derivatives

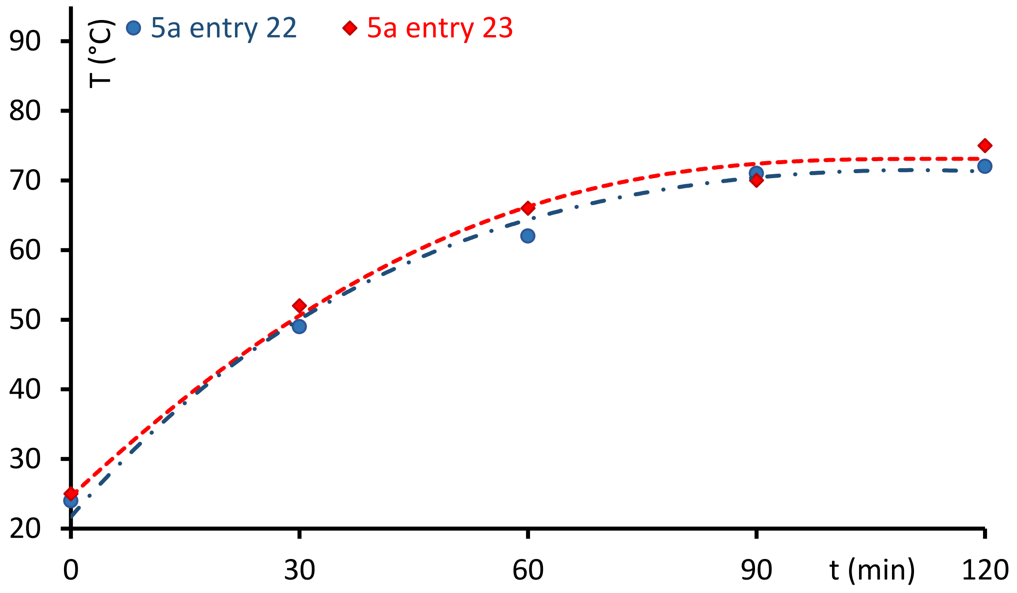

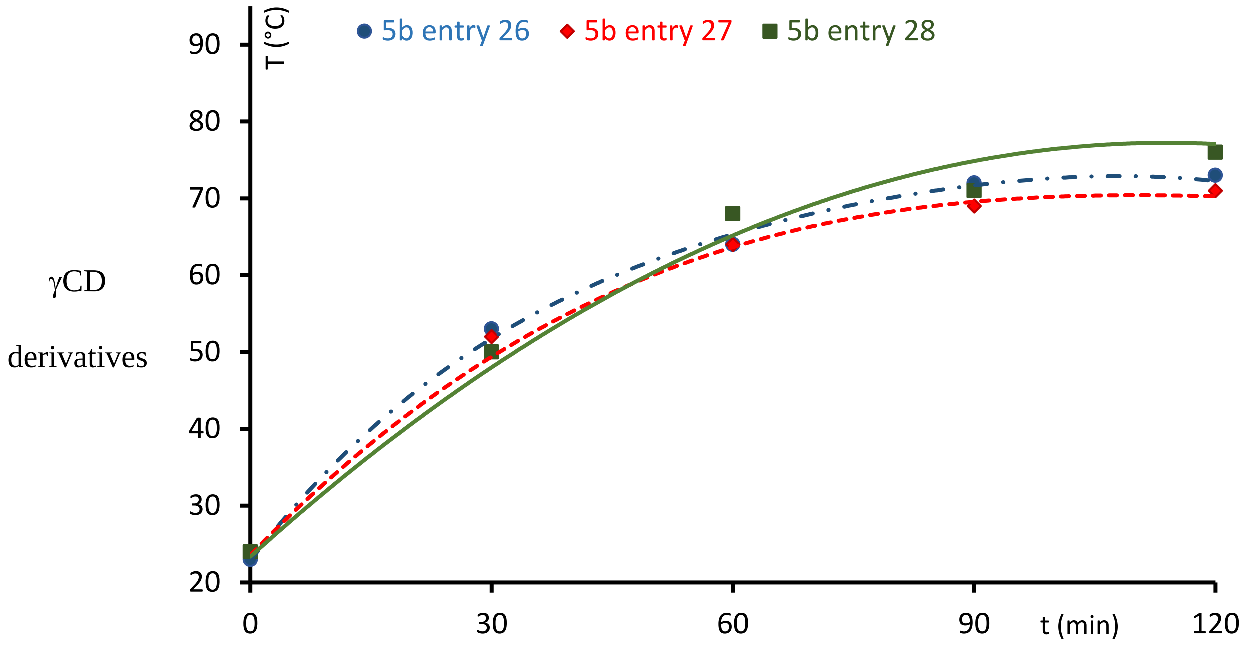

Fig. S 3 Temperature-ball milling time curves of 3-mercaptopropionic acid/KO^{t}Bu/per-6-halogenated CDs

Reaction of per-6-iodo-βCD and 1-dodecanthiol (DDS). DDS (0.22 g, 0.0011 mol) and potassium t-butoxide (0.144 g, 0.0012 mol) were added to the jar, the formed solid was cracked using a spatula and air-dry per-6-iodo-βCD (0.19 g, 0.0001 mol) was added and then mixed with a spatula in the jar. Balls/substance mass ratio was ~127. The balls were added and ball milling was carried out for 120 min. The temperature was checked after 30, 60, 90 and 120 min using the infra thermometer. The jar content was washed MeOH, then acetone, and finally dissolved in methylene chloride and MeOH addition resulted in an impossible-to-filter milk-like solution-suspension. After centrifuging the supernatant was removed and treated with chloroform. The product was only partially soluble in ethanol-free chloroform and DMSO. Yield: 0.23 g, 94.8%.

6a *entry 31* R_f=0.00-0.03 (max. 25 μg; 1,4-dioxane/cc. NH_3-H_2O=10:7).

IR and NMR assignments are in Table S 4, Table S 5, and Table S 6

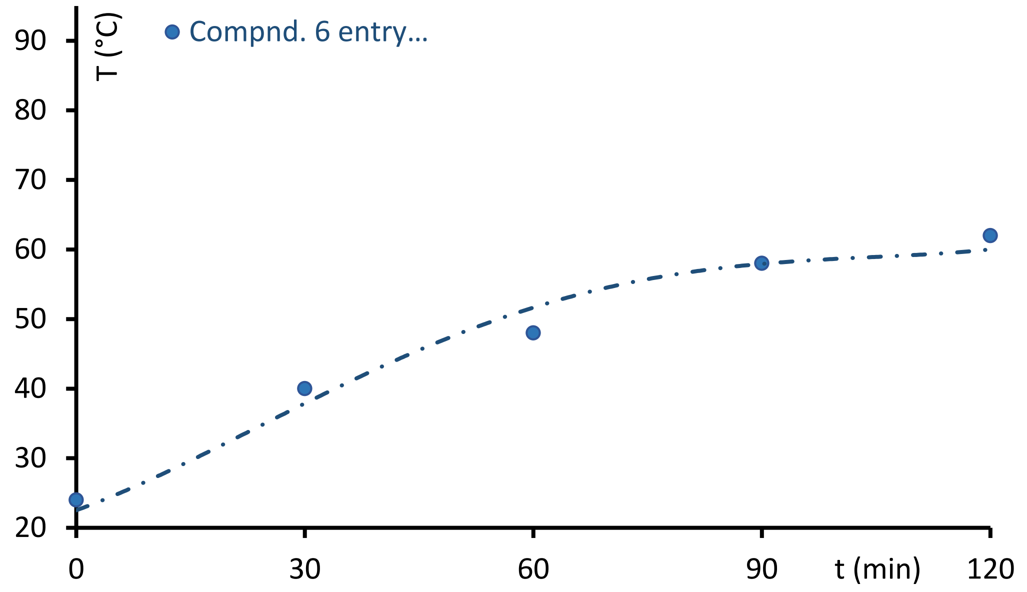

Fig. S 4 Temperature-ball milling time curve of 1-dodecanethiol/KO^{t}Bu/per-6-iodo-βCDs

Table S 4 Characteristic IR absorption bands (cm^{-1}) of the prepared compounds

	N-H	C-H	NH_3^+	N_3	O-C-O*	N=C-NH$_2$	O=C-O$^-$	C-S
				cm^{-1}				
**2a** (I)		2912-3032			1658			
**2a'** (Cl)		2884-2925						
**2b** (I)		2906-3032			1658			
**2b'** (Br)		2832-3038			1654			
**3a** (TU*HI) *entry 4*	3123	2829-2901	2755		1651	1545		636
**3b** (TU*HI) *entry 9*	3120	2934	2747		1651	1556		647
**3b'** (TU*HBr) *entry 11*	3101	2930-2902	2757		1647	1547		655
**4a** (N3) entry 17		2914		2104	1633			
**4b** (N3) entry 20		2927		2107	1658			
**5a** (MPA) entry 23	3054	2819-2901	~2690-2700		1703			668
**5b** (MPA) *entry 28*	3176	2904	2738		1647		1610	643
**6** (DDS) *entry 31*		2848-2950			1662			719

* Overlapped with DMF signals (compounds **2** and **4**) and overlapped with carboxylic signals (compounds **5a** and **5b**).

Comment	I7bCD 5.4 mg sample in KBr 94 mg			***File Name***	D:\DOCS\!NOTEBOOKS\BM_REACTIONS\IR\I7BCD\I7BCD.SPC		
Date Stamp	20/00/1375 06:49:00	***Date***	09 Sep 2015 14:16:34	***Technique***	Infrared	***Instrument***	1005
Spectral Region	NIR-IR	***X Axis***	Wavenumber (cm-1)	***Y Axis***	%Transmittance	***Spectrum Range***	450.0000 - 4400.0000
Points Count	3951	***Data Spacing***	1.0000				

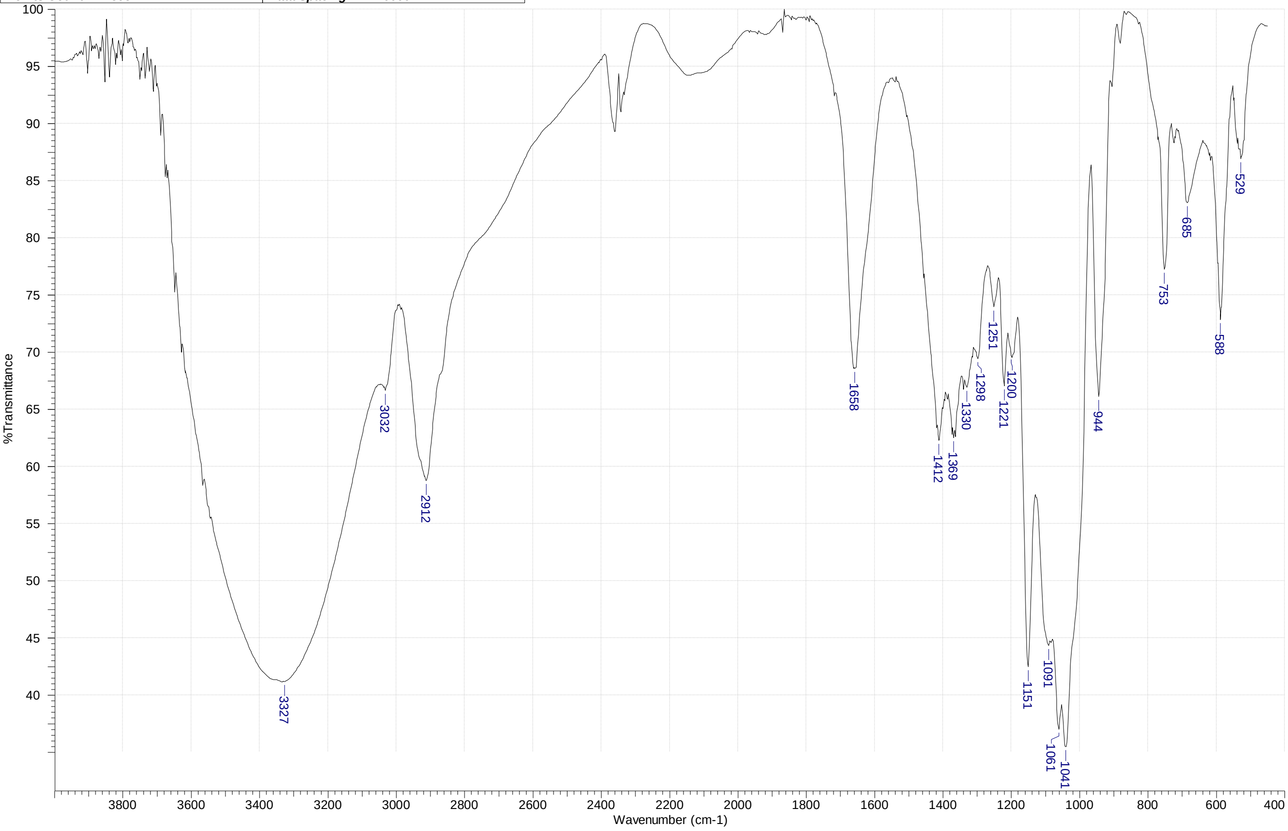

Fig. S 5. IR spectrum of heptakis(6-deoxy-6-iodo)-βCD ***2a***

Title	TK_per6klorBCD	***Origin***	MTA KKKI	***Owner***	Nicolet		
File Name	E:\DOC\!!MANU2_PER\BJOC\REV\CL7BCD\TK_PER6KLORBCD.JDX			***Date Stamp***	11:36:55	***Date***	28 May 2009 11:51:36
Technique	Infrared	***Spectral Region***	IR	***X Axis***	Wavenumber (cm-1)	***Y Axis***	Transmittance
Spectrum Range	400.2256 - 3999.3618	***Points Count***	3733	***Data Spacing***	0.9644		

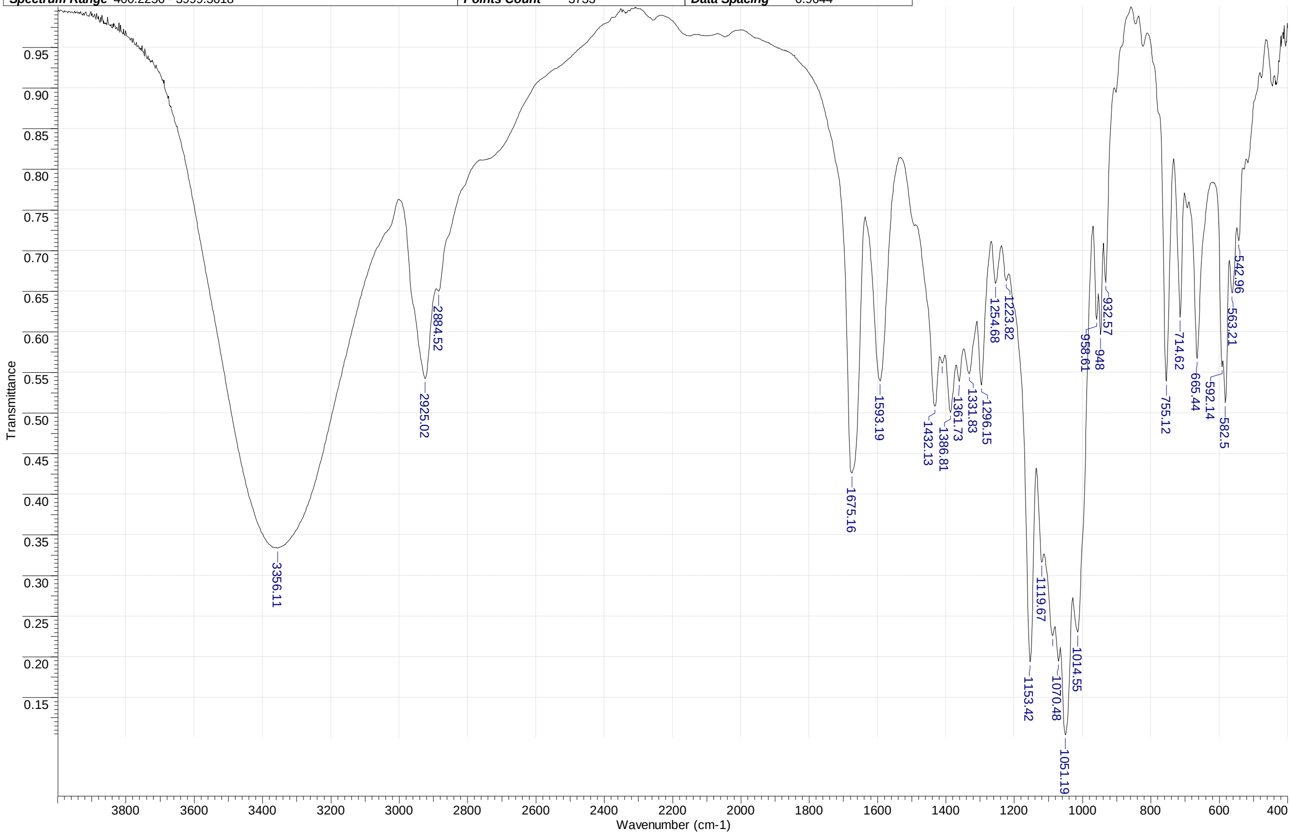

Fig. S 6. IR spectrum of heptakis(6-deoxy-6-chloro)-βCD ***2a'***

Comment	4.3 mg I8gCD + 96 mg KBr (CYL-3615) 012			***File Name***	D:\DOCS\!NOTEBOOKS\BM_REACTIONS\IR\!NEW_IR\!!MANU2\I8GCD_0012.SPC		
Date Stamp	24/07/1393 12:63:00	***Date***	20 Apr 2016 10:58:24	***Technique***	Infrared	***Instrument***	1005
Spectral Region	NIR-IR	***X Axis***	Wavenumber (cm-1)	***Y Axis***	%Transmittance	***Spectrum Range***	400.0000 - 4400.0000
Points Count	4001	***Data Spacing***	1.0000				

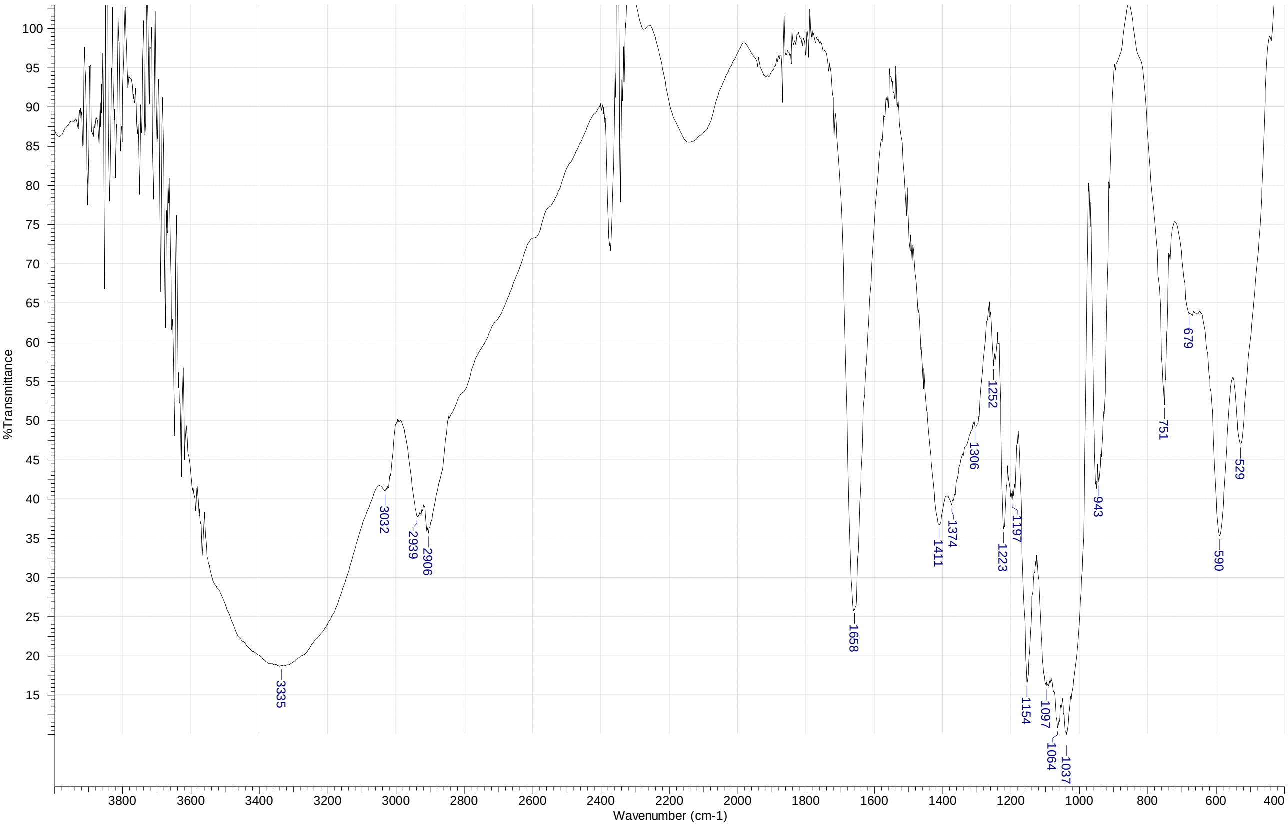

Fig. S 7. IR spectrum of octakis(6-deoxy-6-iodo)-γCD ***2b***

Comment	4.0 mg Br8gCD + 94 mg KBr			***File Name***	D:\DOCS\!NOTEBOOKS\BM_REACTIONS\IR\!NEW_IR\!!MANU2\BR8GCD_0013.SPC		
Date Stamp	05/09/1393 23:57:00	***Date***	21 Apr 2016 12:46:02	***Technique***	Infrared	***Instrument***	1005
Spectral Region	NIR-IR	***X Axis***	Wavenumber (cm-1)	***Y Axis***	%Transmittance	***Spectrum Range***	400.0000 - 4400.0000
Points Count	4001	***Data Spacing***	1.0000				

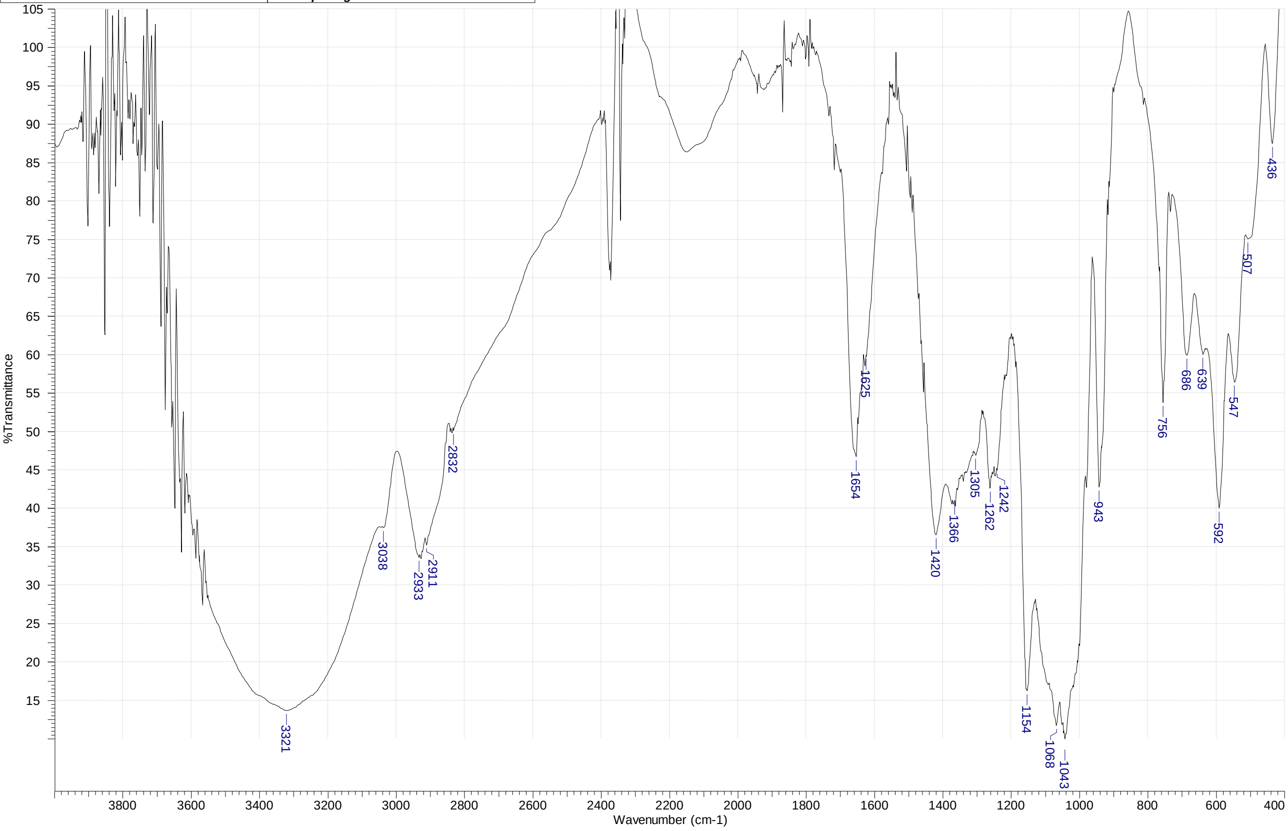

Fig. S 8. IR spectrum of octakis(6-bromo-6-deoxy)-γCD ***2b'***

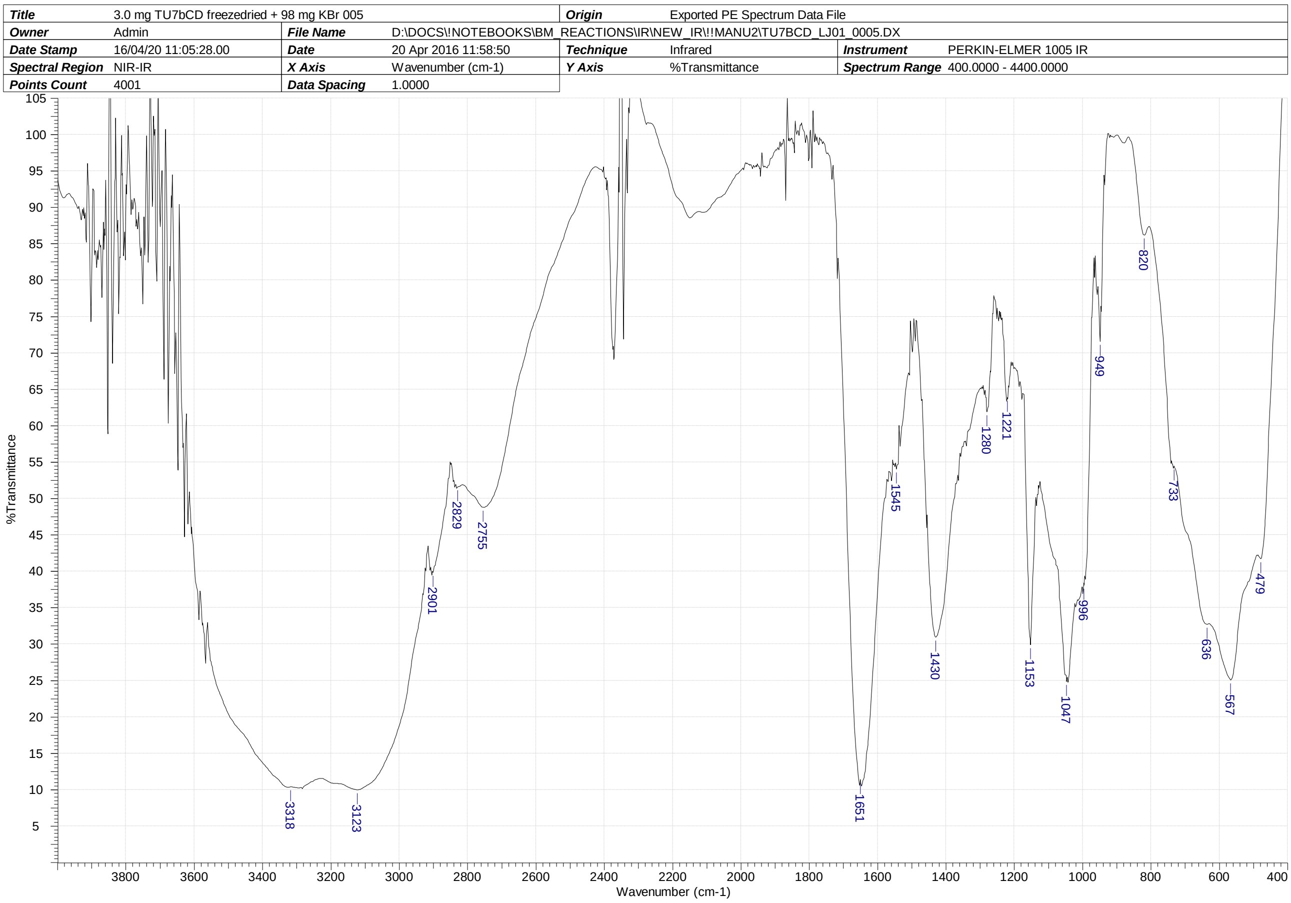

*Fig. S 9. IR spectrum of heptakis(6-deoxy-6-S-thiuronium)-βCD iodide **3a** entry 4*

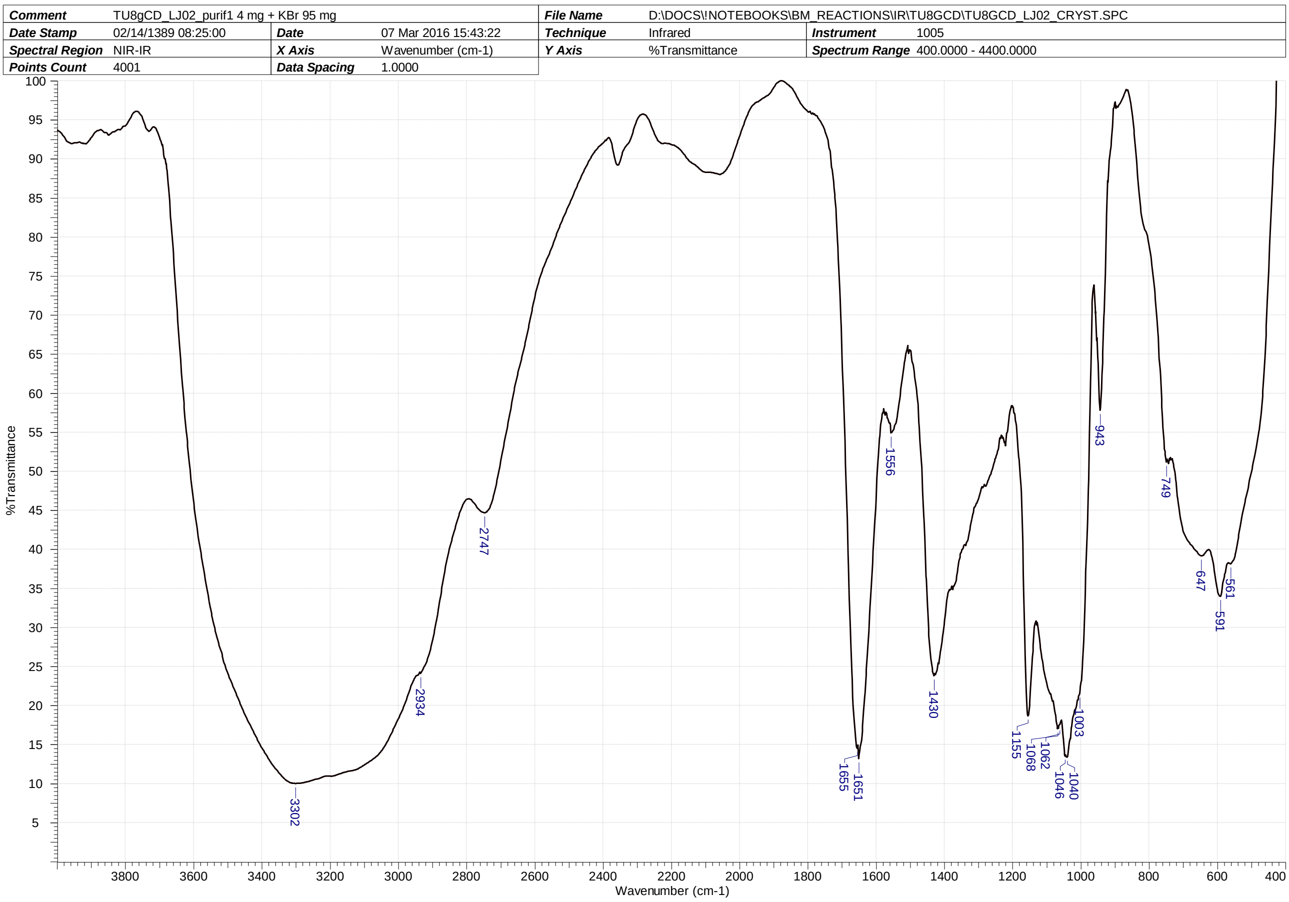

*Fig. S 10. IR spectrum of octakis(6-deoxy-6-S-thiuronium)-γCD iodide **3b** entry 9*

Comment	4.0 mg TU8gCD LJ03 freezdried + 98 mg KBr 006b repeated						
File Name	D:\DOCS\!NOTEBOOKS\BM_REACTIONS\IR\!NEW_IR\!!MANU2\TU8GCD_LJ03_0006B_MANUAL.SPC					***Date Stamp***	26/07/1393 06:61:00
Date	20 Apr 2016 12:00:14	***Technique***	Infrared	***Instrument***	1005	***Spectral Region***	NIR-IR
X Axis	Wavenumber (cm-1)	***Y Axis***	%Transmittance	***Spectrum Range***	400.0000 - 4400.0000	***Points Count***	4001
Data Spacing	1.0000						

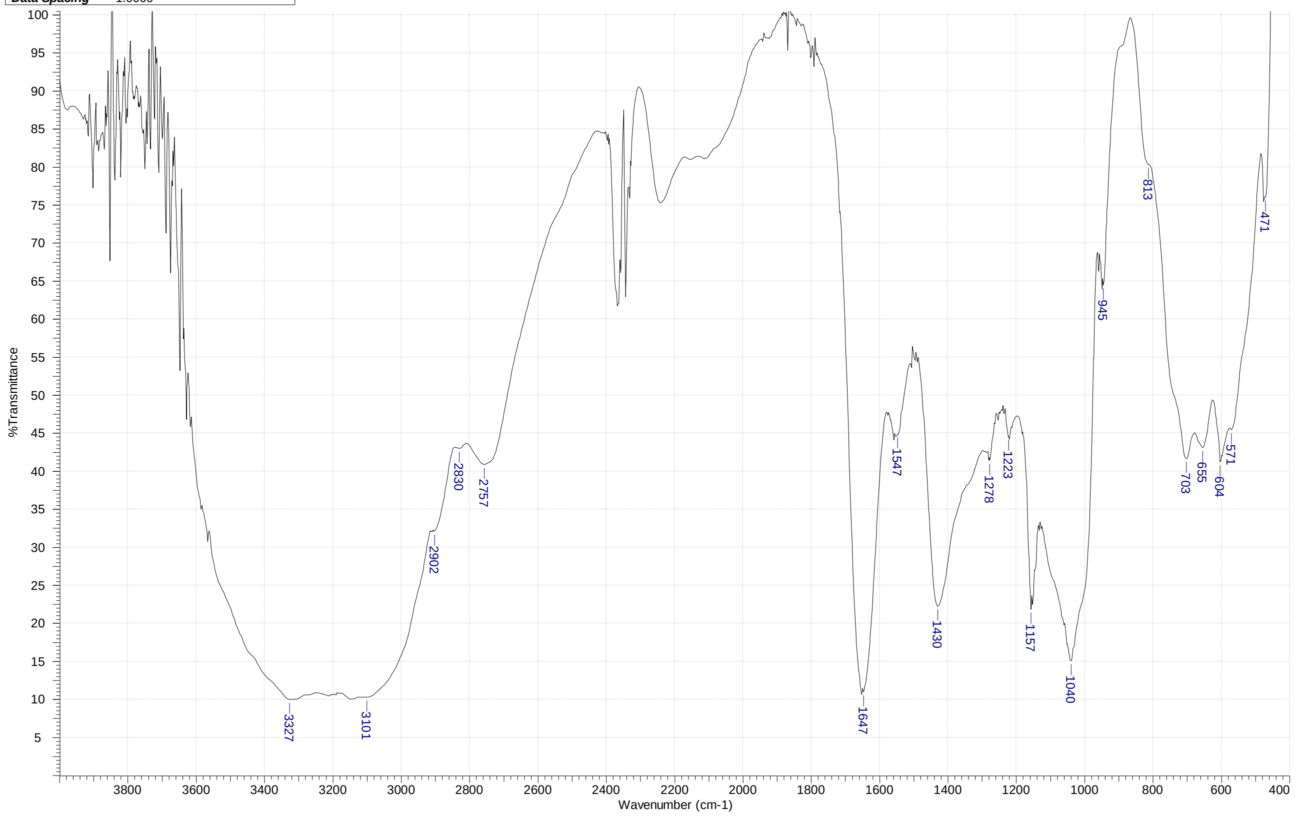

*Fig. S 11. IR spectrum of octakis(6-deoxy-6-S-thiuronium)-γCD bromide **3b'** entry 11*

Comment	Az7bCD 5 mg + KBr 95 mg			***File Name***	D:\DOCS\!NOTEBOOKS\BM_REACTIONS\AZ7BCD\Az7BCD_LJ03_crude.SPC		
Date Stamp	27/00/1389 15:53:00	***Date***	26 Feb 2016 15:12:54	***Technique***	Infrared	***Instrument***	1005
						Spectral Region	NIR-IR
X Axis	Wavenumber (cm-1)	***Y Axis***	%Transmittance	***Spectrum Range***	400.0000 - 4400.0000	***Points Count***	4001
						Data Spacing	1.0000

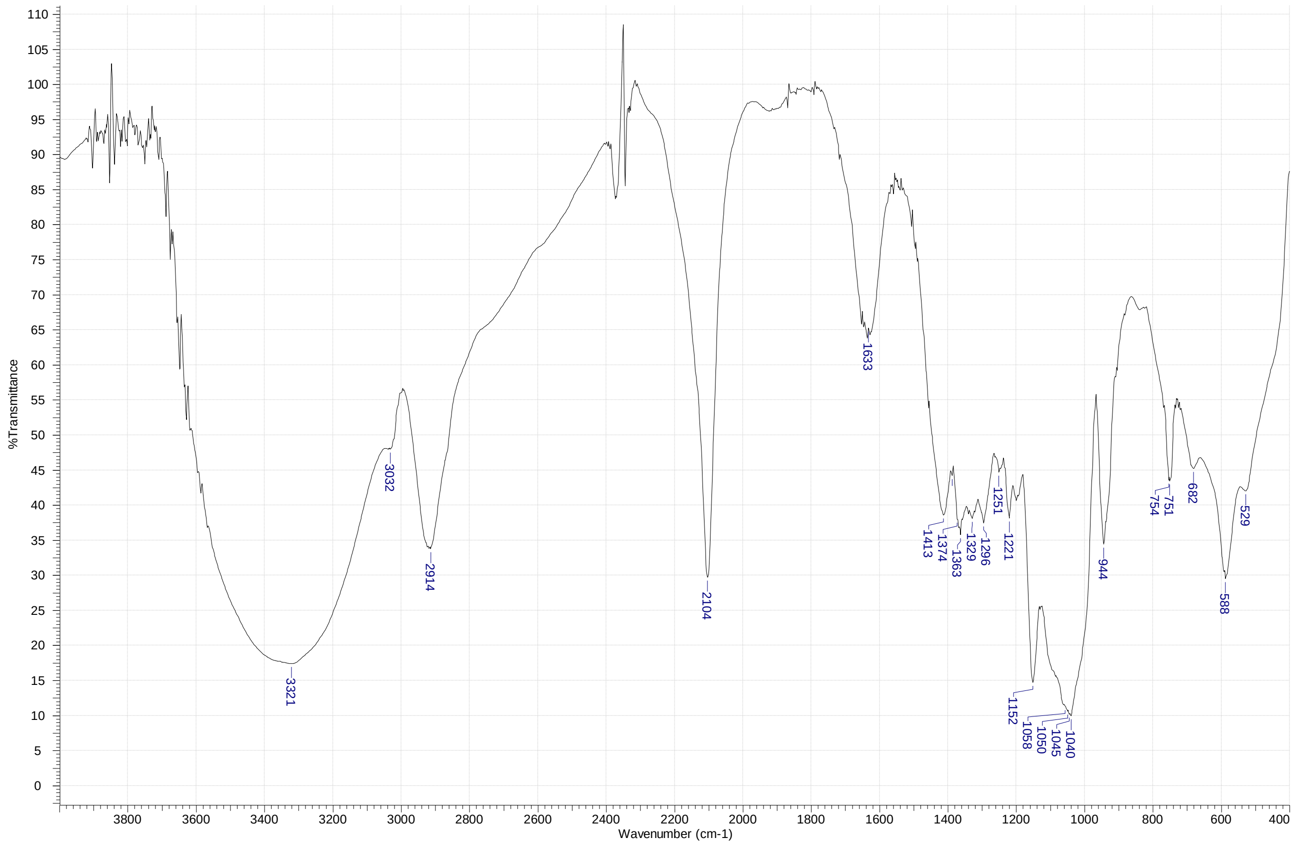

*Fig. S 12. IR spectrum of heptakis(6-azido-6-deoxy)-βCD **4a** entry 17*

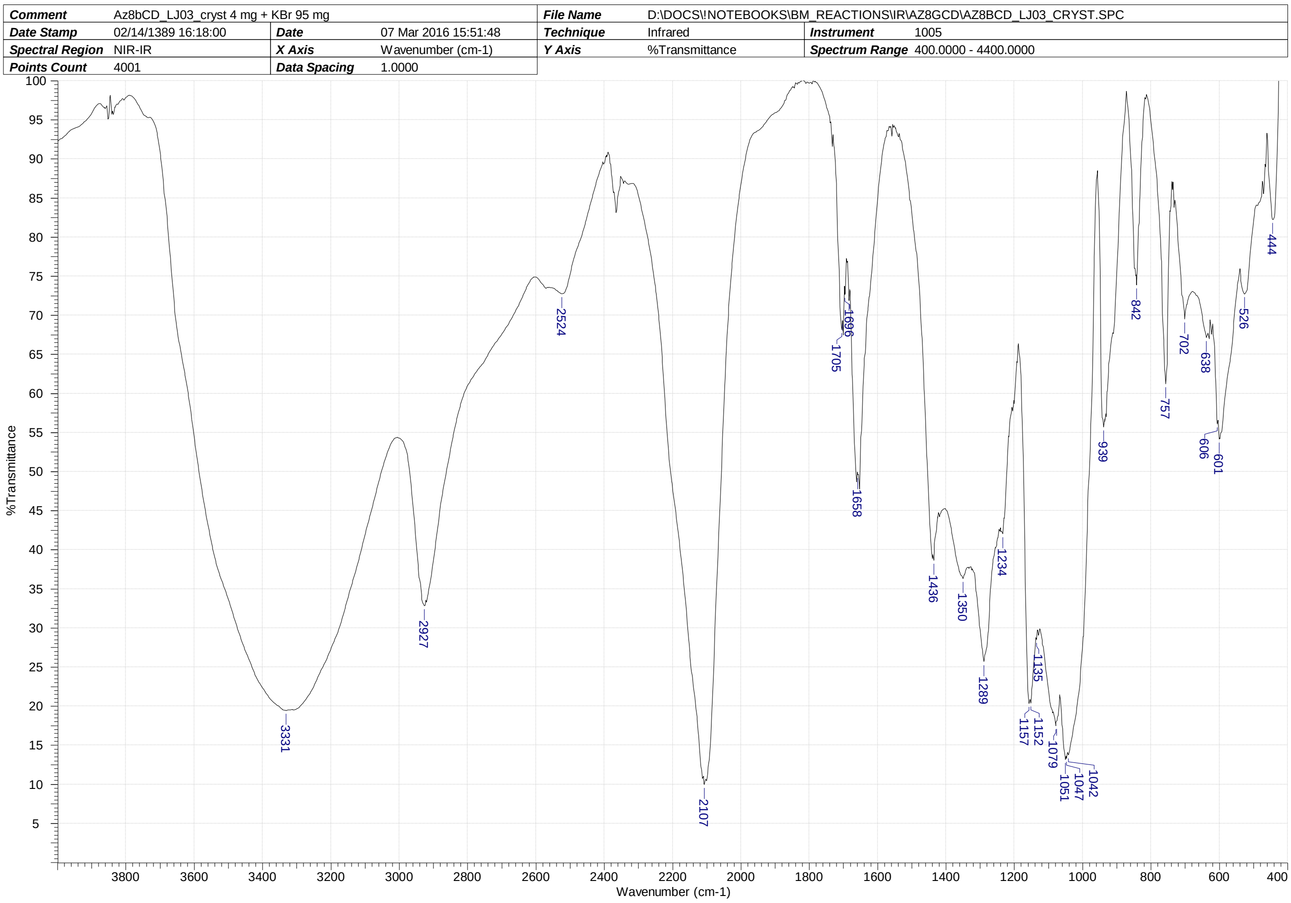

*Fig. S 13. IR spectrum of octakis(6-azido-6-deoxy)-γCD **4b** entry 20*

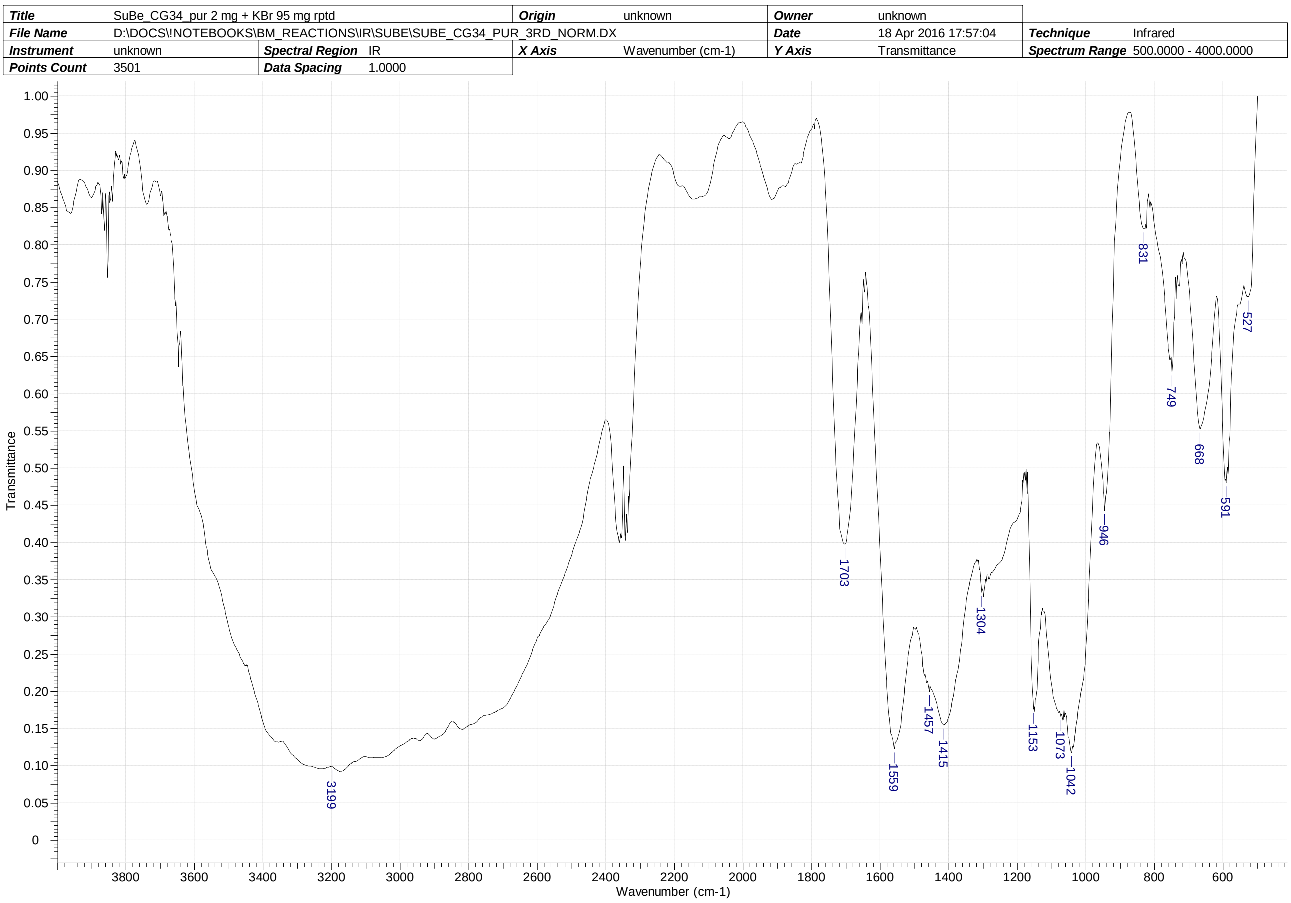

*Fig. S 14. IR spectrum of heptakis(6-deoxy-6-S-(3-mercapto)propionyl))-βCD NH_4^+ **5a** entry 23*

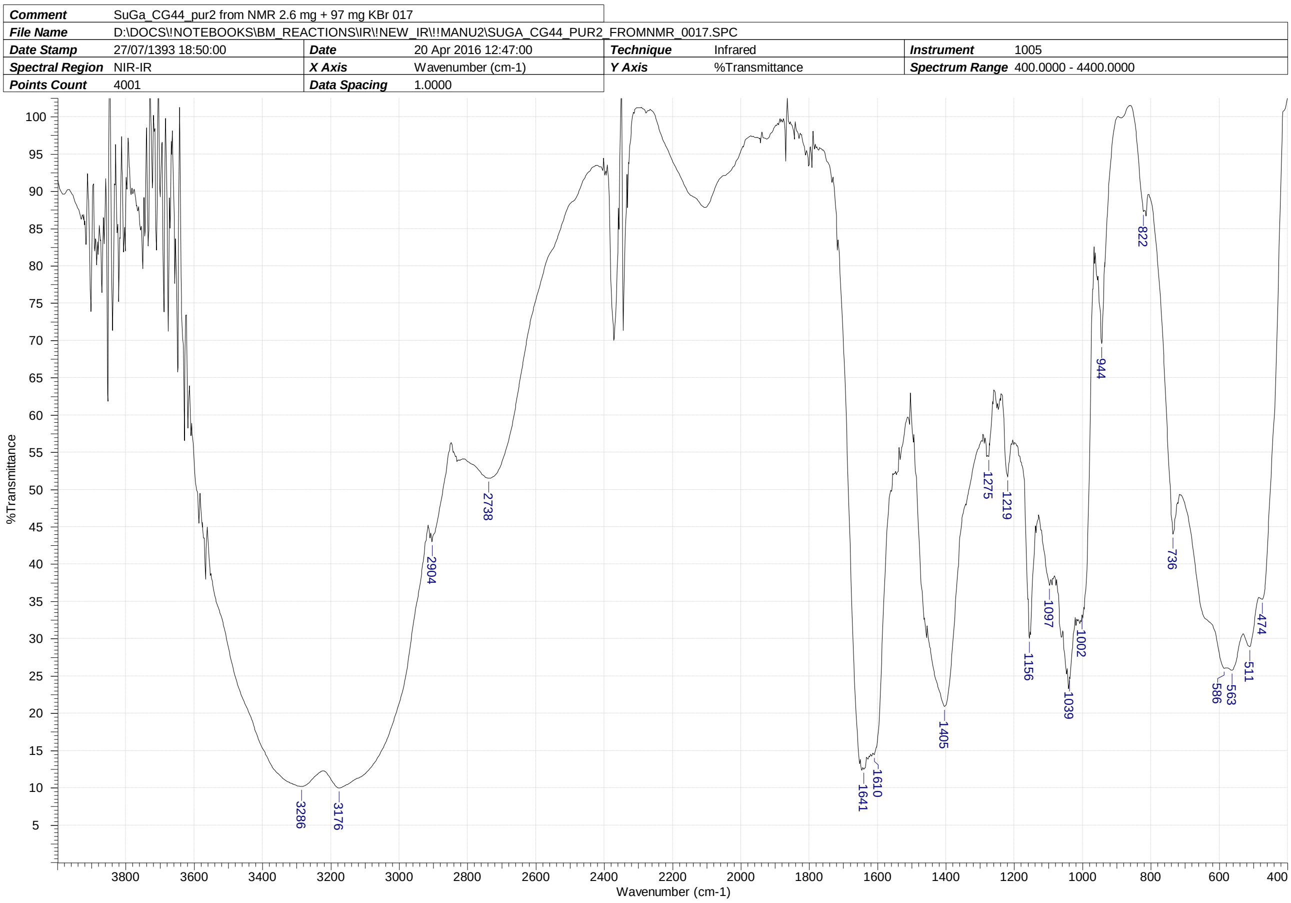

Fig. S 15. IR spectrum of octakis(6-deoxy-6-S-(3-mercapto)propionyl))-γCD NH_4^+ ***5b*** *entry 28*

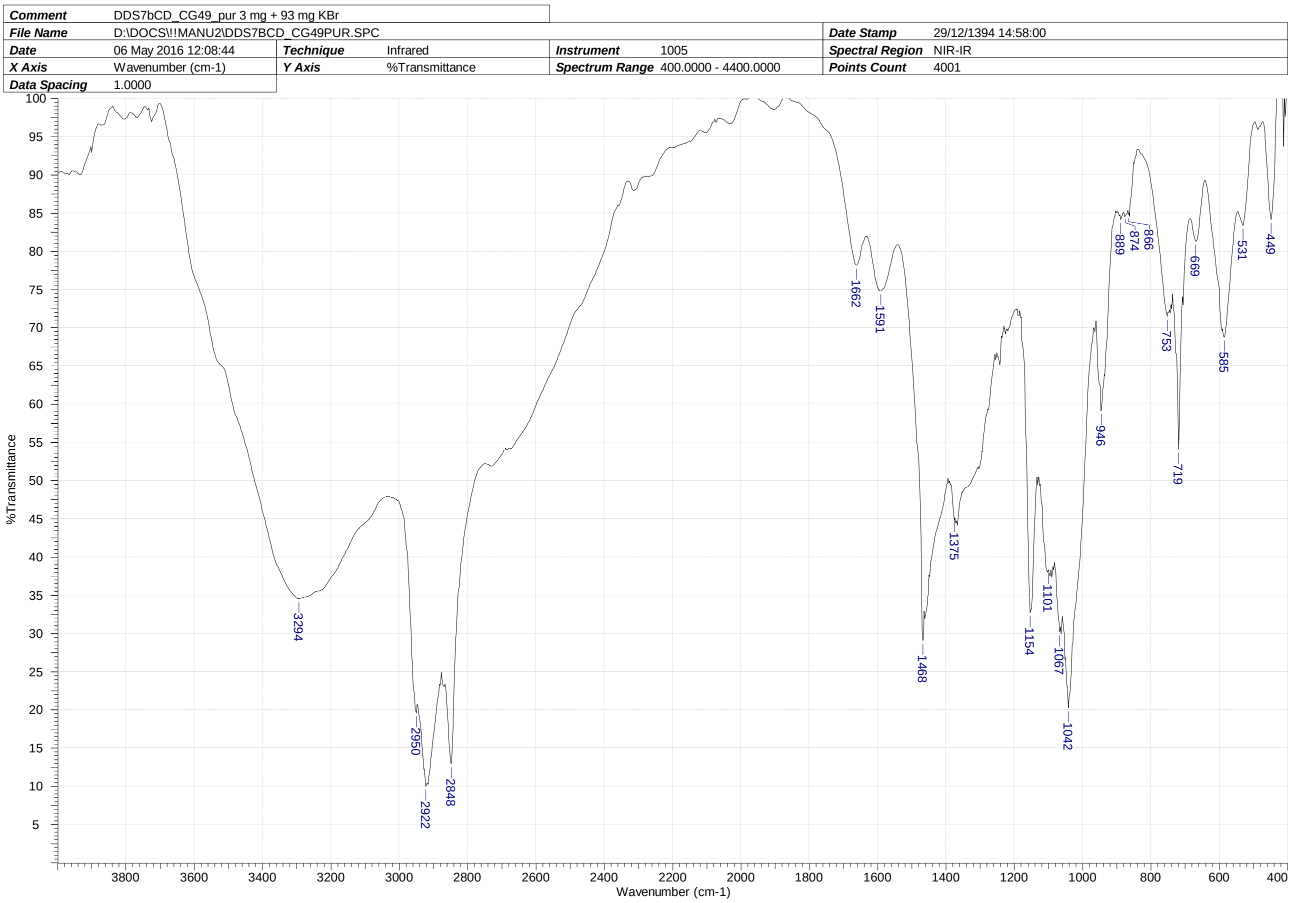

Fig. S 16. IR spectrum of heptakis(6-deoxy-6-S-(1-dodeclythio))-βCD ***6*** *entry 31*

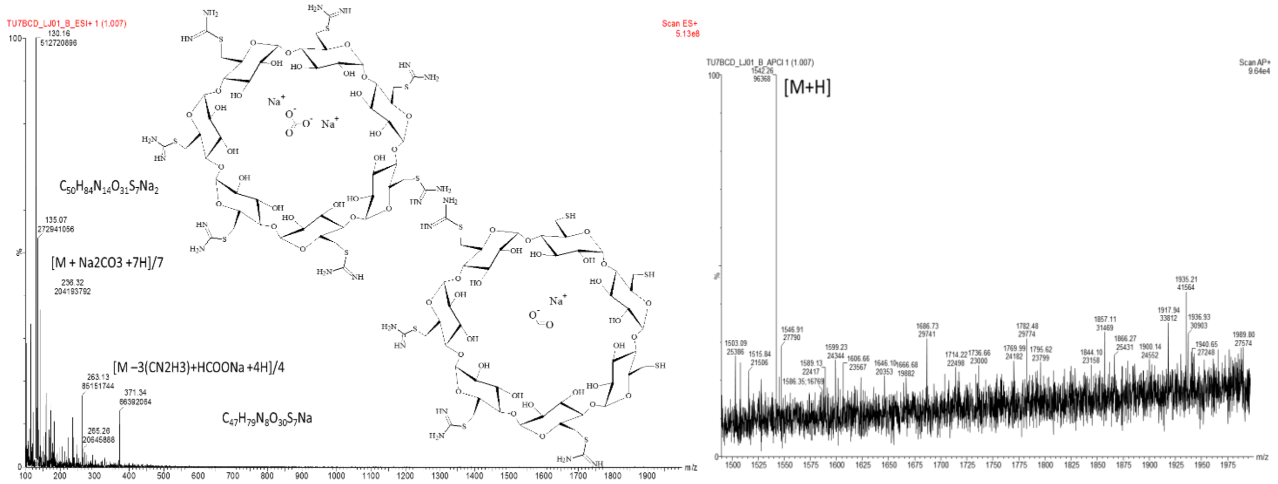

Fig. S 17. ESI-MS spectrum of heptakis(6-deoxy-6- S-thioureido)-βCD **3a** entry 4

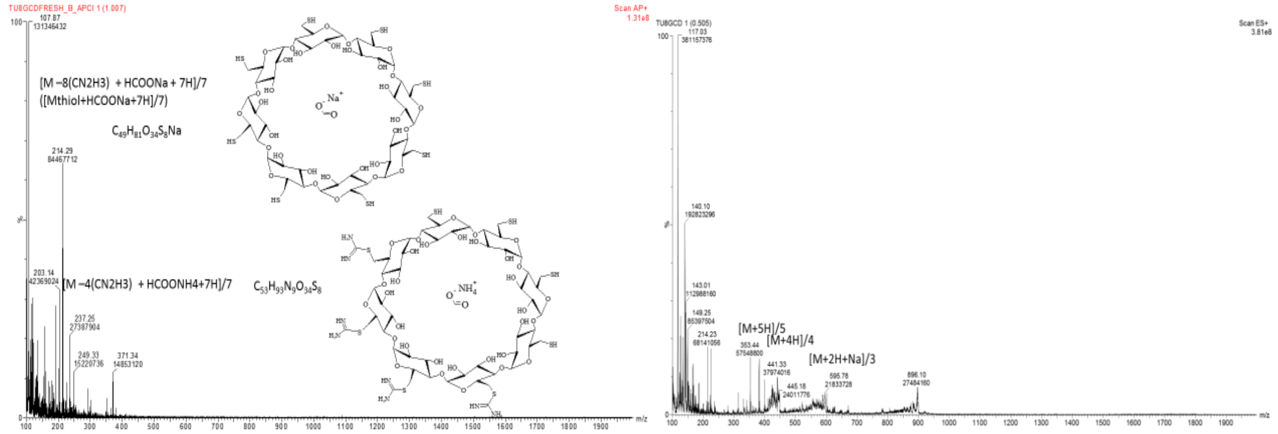

Fig. S 18. ESI-MS spectrum of octakis(6-deoxy-6- S-thioureido)-γCD **3b** entry 11

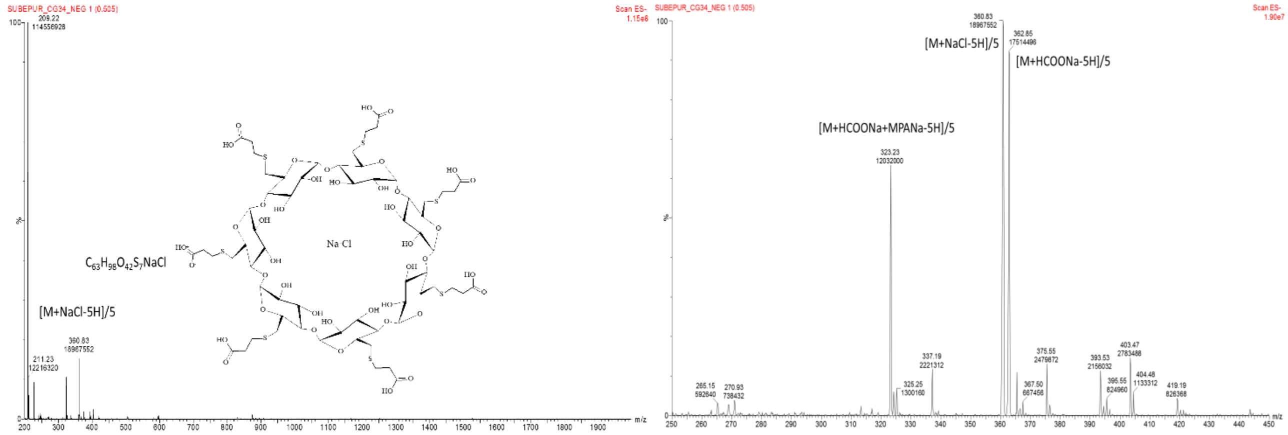

Fig. S 19. ESI-MS spectrum of heptakis(6-deoxy-6-S-(3-mercapto)propionyl)-βCD **5a** entry 23

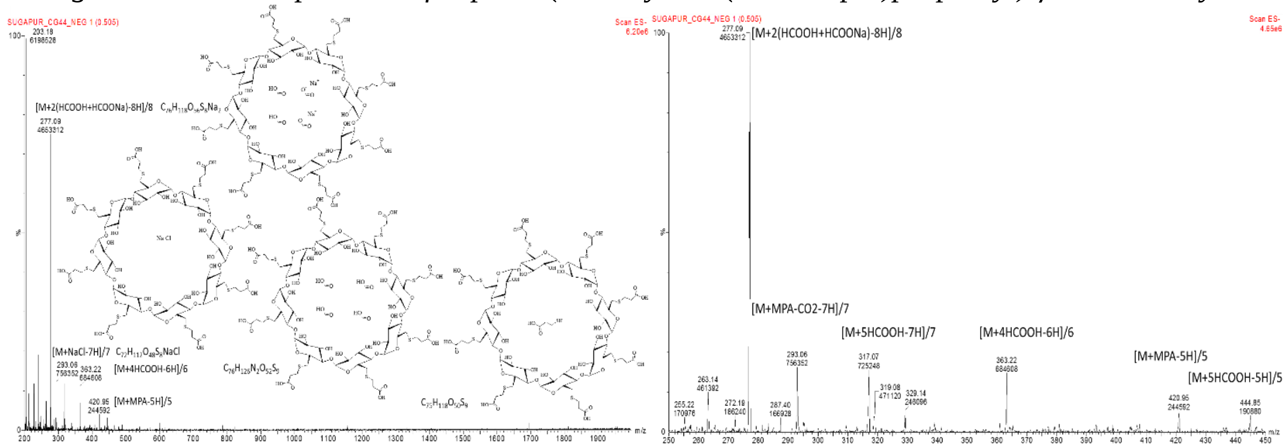

Fig. S 20. ESI-MS spectrum of octakis(6-deoxy-6-S-(3-mercapto)propionyl)-γCD **5b** entry 28

Table S 5 Proton NMR assignment (ppm) of compounds (from ^{1}H and HSQC spectra)

	H1	H2	H3	H4	H5	H6	α	β		ω
**2a** (I)	5.03	3.33-3.50	3.56-3.73	3.25-3.41	3.65-3.76	3.77-3.92,<br>3.40-3.55				
**2a'** (Cl)	4.95	3.38	3.63	3.37	3.83	3.79,4.07				
**2b** (I)	5.07	3.35-3.48	3.57-3.72	3.25-3.38	3.57-3.72	3.86,3.45				
**2b’** (Br)	5.06	3.26-3.53	3.57-3.75	3.22-3.58	3.72-3.97	3.85-4.17,<br>3.52-3.85				
**3a** (TU*HI)<br>*entry 4*	5.17	3.61-3.76	3.87-4.03	3.52-3.68	4.31-4.45	3.65-3.80,<br>3.39-3.54				
**3b** (TU*HI)<br>*entry 9*	5.20	3.60-3.76	3.84-3.99	3.48-3.68	4.25-4.40	3.66-3.82,<br>3.38-3.53				
**3b’**<br>(TU*HBr)<br>*entry 11*	5.16	3.58-3.72	3.81-3.94	3.50-3.63	4.16-4.28	3.62-3.72,<br>3.37-3.49				
**4a** (N_3)<br>entry 17	4.94	3.29-3.52	3.54-3.76	3.28-3.48	3.71-3.90	3.52-3.92				
**4b** (N_3)<br>entry 20	4.97	3.36-3.50	3.54-3.67	3.32-3.46	3..71-3.84	3.69-3.83,<br>3.54-3.68				
**5a** (MPA)<br>entry 23	5.04	3.47-3.57	3.78-3.92	3.46-3.62	3.88-3.98	2.98-3.16,<br>2.84-2.98	2.72-<br>2.86	2.34-<br>2.50		
**5b** (MPA)<br>*entry 28*	5.13	3.50-3.65	3.88	3.40-3.64	3.98	3.05-3.20,<br>3.86-2.98	2.75-<br>2.88	2.40-<br>2.58		
**6** (DDS)<br>*entry 31*	4.86-5.03	3.32-3.46	3.58-3.76	3.26-3.43	3.74-3.90	3.00-3.15,<br>2.80-2.88	2.65-<br>2.74	1.57-<br>1.72	1.18-<br>1.58	0.83-<br>0.96

Table S 6 Carbon NMR assignment (ppm) of compounds (from HSQC spectra)

	C1	C2	C3	C4	C5	C6	α	β		ω
**2a** (I)	103.23	73.14	72.53	87.11	73.75	9.68,9.91				
**2a'** (Cl)	101.94	71.85	72.37	83.47	71.03	44.73,44.78				
**2b** (I)	102.22	72.8	71.99	85.73	71.99	9.75,9.87				
**2b’** (Br)	102.11	72.77	72.47	84.47	71.82	35.22				
**3a** (TU*HI) *entry 4*	102.09	72.26	72.85	85.25	71.65	33.53,33.57				
**3b** (TU*HI) *entry 9*	102.70	73.03	72.96	85.10	72.31	34.14,34.23				
**3b’** (TU*HBr) *entry 11*	101.94	71.96	72.12	84.18	71.18	33.12,33.15				
**4a** (N_3) entry 17	103.24	73.05	72.79	84.37	71.41	52.07				
**4b** (N_3) entry 20	102.41	72.74	73.04	83.42	70.99	51.70,51.88				
**5a** (MPA) entry 23	100.67	71.92	71.31	82.90	72.18	33.83,32.56	34.7	37.57		
**5b** (MPA) *entry 28*	101.5	72.67	72.69	82.97	72.59	34.38,33.90	30.22	37.76		
**6** (DDS) *entry 31*	102.48	72.58	72.84	85.74	71.53	33.68,33.92	32.81	29.95	29.99	14.91

Acquisition Time (sec)	3.6438	***Comment***	I7BCD_1H_DMSO-d6_110316_1747_RG=90			***Date***	10 Mar 2016 16:00:16		
Date Stamp	10 Mar 2016 16:00:16			***File Name***	D:\Docs\!Notebooks\BM_Reactions\NMR\I7BCD\1\fid			***Frequency (MHz)***	300.13
Nucleus	1H	***Number of Transients***	64	***Origin***	spect	***Original Points Count***	16384	***Owner***	root
Points Count	131072	***Pulse Sequence***	zg	***Receiver Gain***	90.50	***SW(cyclical) (Hz)***	4496.40	***Solvent***	DMSO-d6
Spectrum Offset (Hz)	1091.5022	***Spectrum Type***	STANDARD	***Sweep Width (Hz)***	4496.37	***Temperature (degree C)***	20.060		

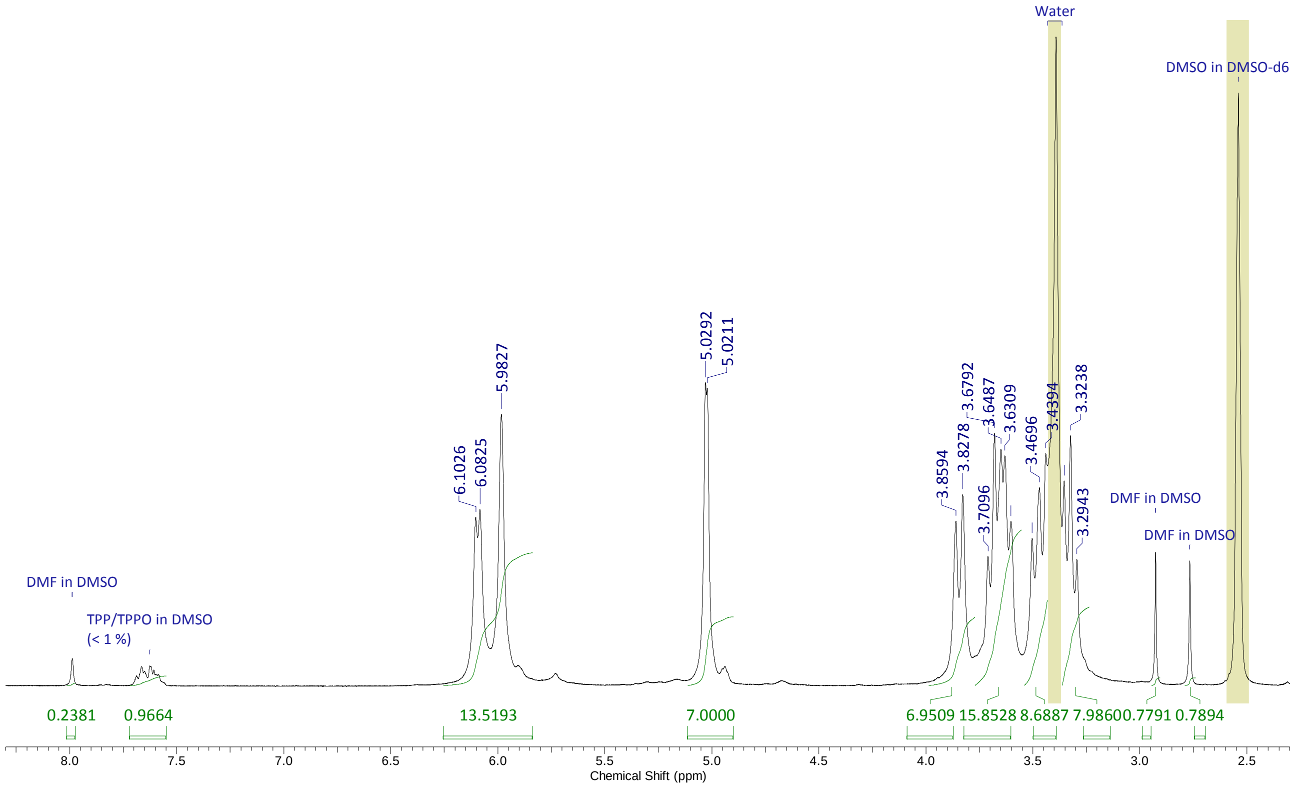

Fig. S 21. ^{1}H-NMR spectrum of heptakis(6-deoxy-6-iodo)-βCD ***2a***

Acquisition Time (sec)	(0.0427, 0.0051)	***Comment***	5 mm BBO 1H-BB Z-GRD Z8284/0059			***Date***	10 Mar 2016 16:53:58
File Name	D:\Docs\!Notebooks\BM_Reactions\NMR\I7BCD\2\ser			***Frequency (MHz)***	(300.13, 75.47)	***Nucleus***	(1H, 13C)
Number of Transients	32	***Origin***	spect	***Original Points Count***	(128, 64)	***Owner***	psm
Points Count	(512, 512)	***Pulse Sequence***	hsqcedetgp	***Solvent***	DMSO	***Spectrum Type***	HSQC-DEPT
Sweep Width (Hz)	(2991.75, 12475.59)	***Temperature (degree C)***	20.160	***Title***	I7BCD_HSQC_DMSO-d6_110316_1747_RG=90		

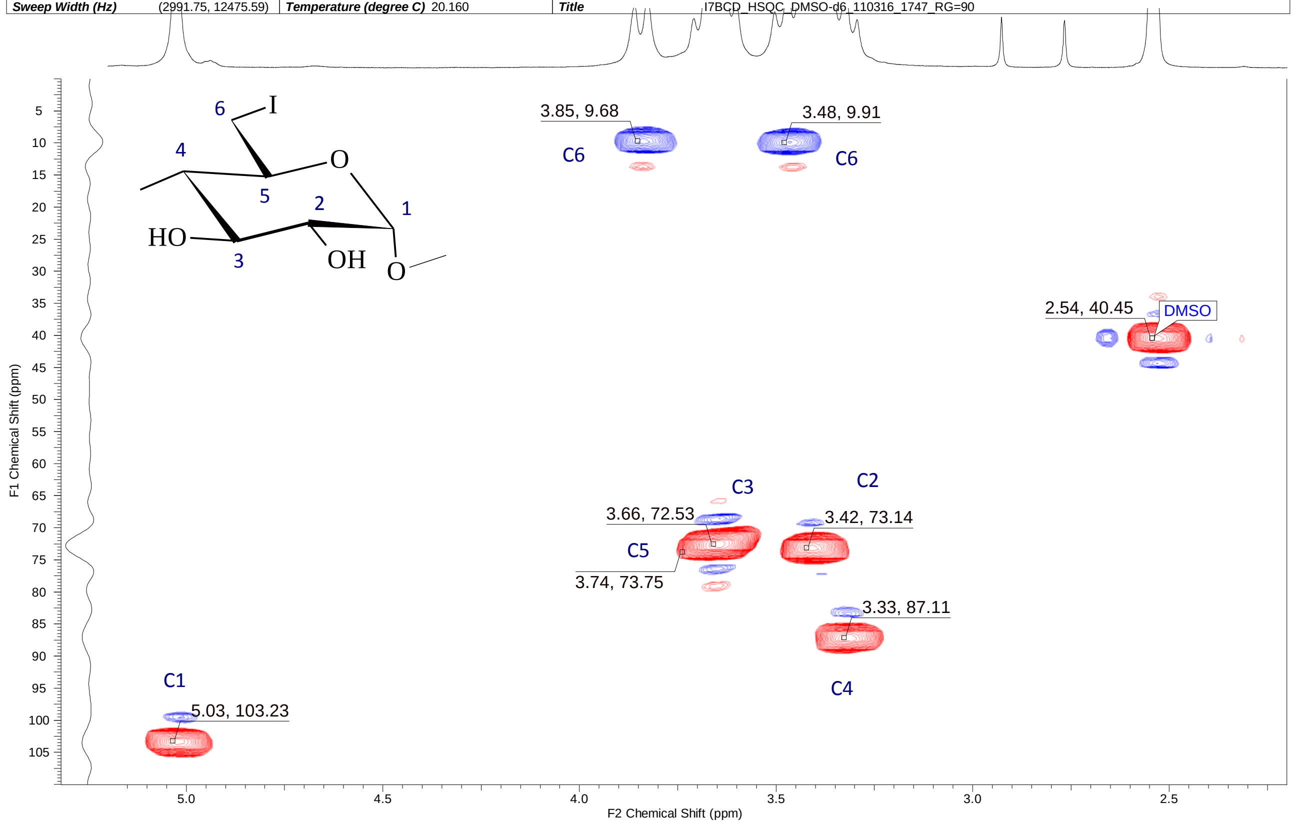

Fig. S 22. HSQC-NMR spectrum of heptakis(6-deoxy-6-iodo)-βCD ***2a***

Acquisition Time (sec)	3.9999	***Comment***	samplename: TK_per6klorBCD			***Date***	Mar 11 2010	***Date Stamp***	Mar 11 2010
File Name	Z:\!!manu2_per\BJOC\rev\Cl7bCD\TK_per6klorBCD_2010-03-12_H1-C13_s2pul_dmso_25C_ax_8839_01.fid\fid					***Frequency (MHz)***	399.91	***Nucleus***	1H
Number of Transients	16	***Original Points Count***	39999	***Points Count***	262144	***Pulse Sequence***	s2pul	***Receiver Gain***	20.00
Solvent	DMSO-d6	***Spectrum Offset (Hz)***	1956.3031	***Spectrum Type***	STANDARD	***Sweep Width (Hz)***	10000.00	***Temperature (degree C)***	25.000

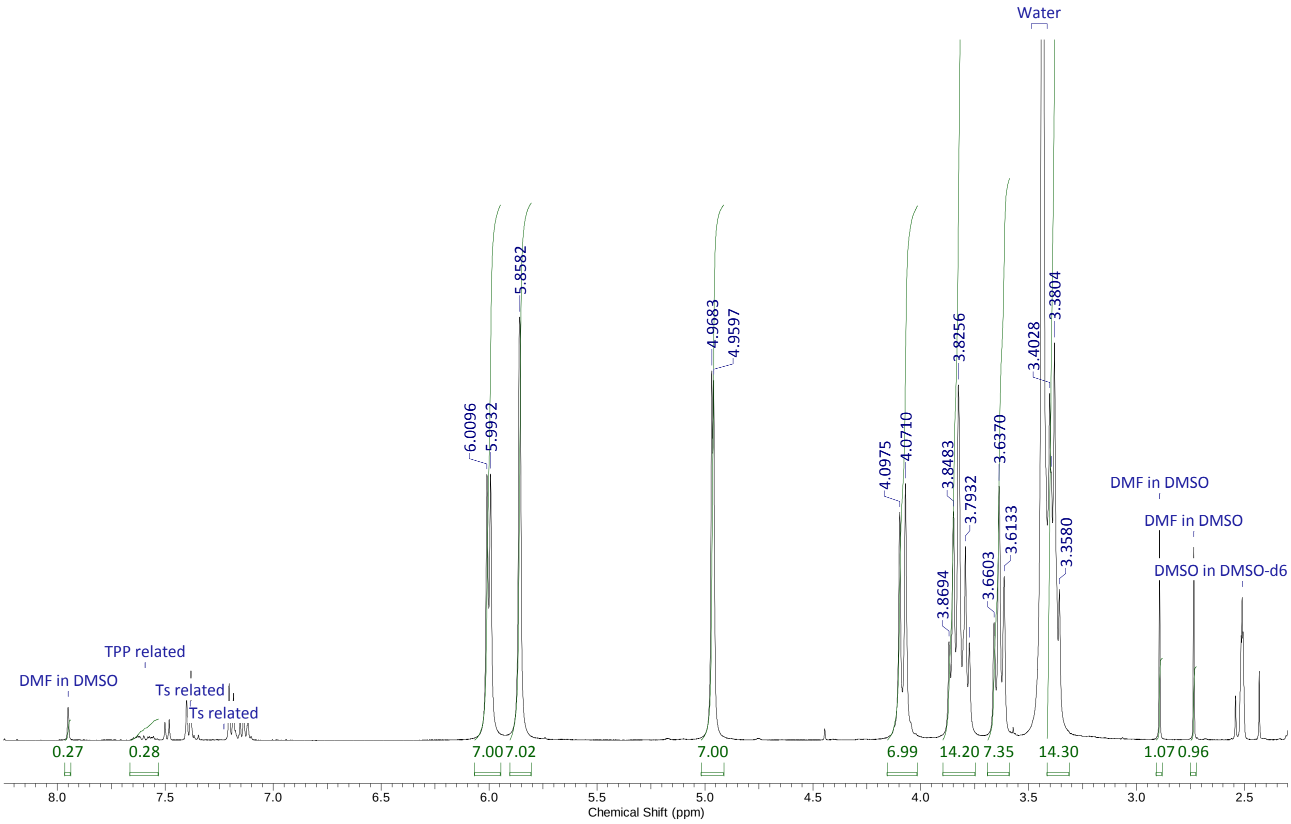

*Fig. S 23. ^{1}H-NMR spectrum of heptakis(6-deoxy-6-chloro)-βCD **2a'***

Acquisition Time (sec)	(0.1500, 0.0090)	***Comment***	samplename: TK_per6klorBCD solvent: dmso temp: 25C probe: ax_8839 2010.03.11. Toke O.				
Date	12 Mar 2010 07:36:24	***Date Stamp***	Mar 11 2010				
File Name	Z:\!!manu2_per\BJOC\rev\Cl7bCD\TK_2010-03-12_H1-C13_gHSQC_dmso_25C_ax_8839_01.fid\fid					***Frequency (MHz)***	(399.91, 100.57)
Nucleus	(1H, 13C)	***Number of Transients***	16	***Original Points Count***	(1500, 256)	***Points Count***	(4096, 1024)
Pulse Sequence	gHSQC	***Solvent***	dmso	***Spectrum Type***	HSQC	***Sweep Width (Hz)***	(10000.00, 28409.09)

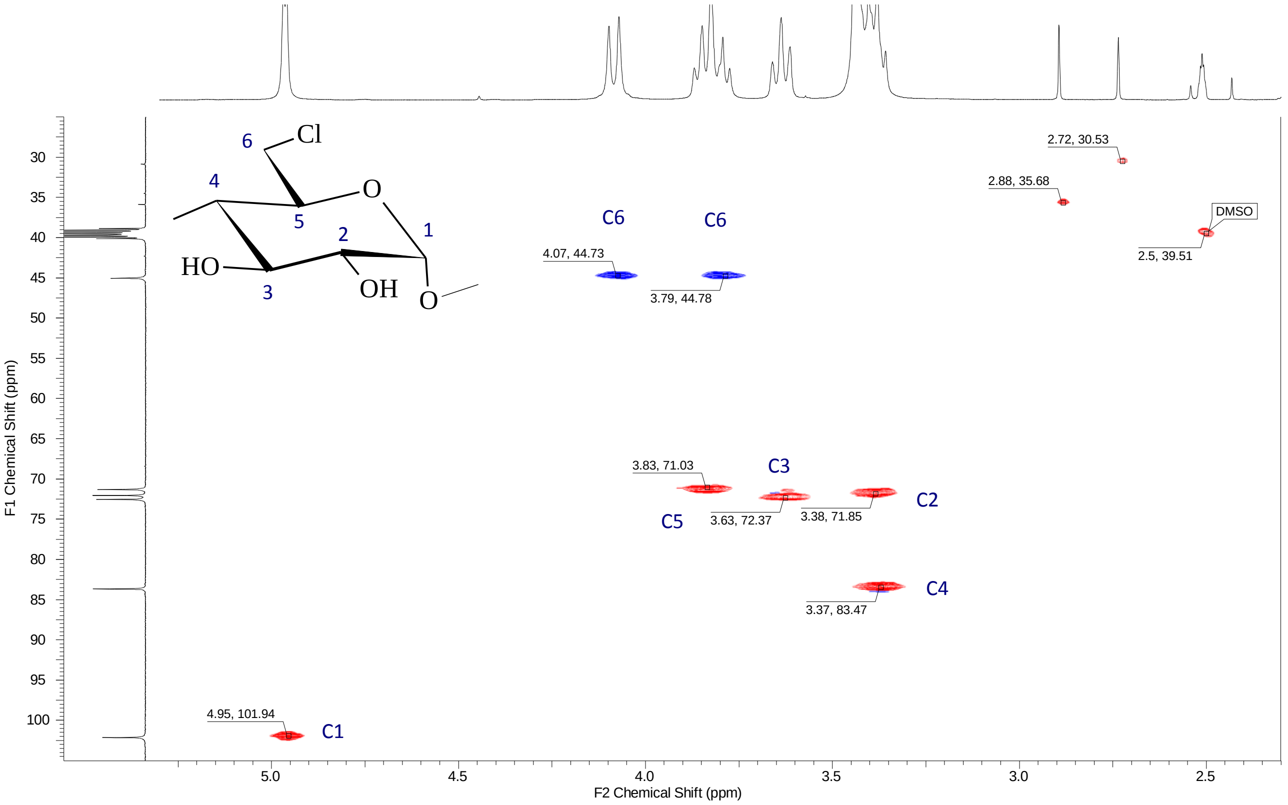

*Fig. S 24. HSQC-NMR spectrum of heptakis(6-deoxy-6-chloro)-βCD **2a'***

Acquisition Time (sec)	3.6438	***Comment***	I8gCD_1H_DMSO-d6_290416_1832_RG=110			***Date***	29 Apr 2016 10:40:32		
Date Stamp	29 Apr 2016 10:40:32			***File Name***	D:\Docs\!Notebooks\BM_Reactions\NMR\I8GCD\1\fid			***Frequency (MHz)***	300.13
Nucleus	1H	***Number of Transients***	64	***Origin***	spect	***Original Points Count***	16384	***Owner***	root
Points Count	131072	***Pulse Sequence***	zg	***Receiver Gain***	114.00	***SW(cyclical) (Hz)***	4496.40	***Solvent***	CDCl3
Spectrum Offset (Hz)	1091.4679	***Spectrum Type***	STANDARD	***Sweep Width (Hz)***	4496.37	***Temperature (degree C)***	19.760		

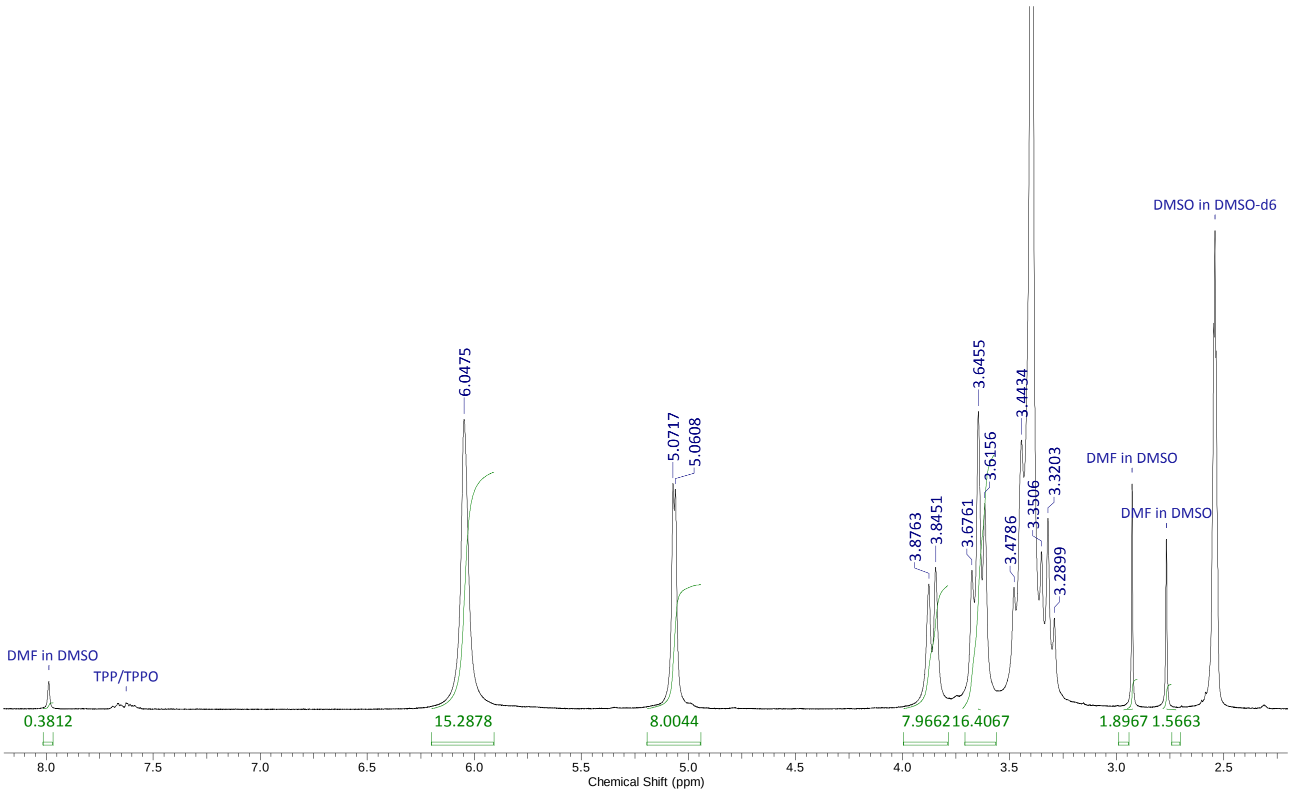

*Fig. S 25. ^{1}H-NMR spectrum of octakis(6-deoxy-6-iodo)-γCD **2b***

Acquisition Time (sec)	(0.0427, 0.0051)	***Comment***	5 mm BBO 1H-BB Z-GRD Z8284/0059			***Date***	29 Apr 2016 11:33:56
File Name	D:\Docs\!Notebooks\BM_Reactions\NMR\I8GCD\2\ser			***Frequency (MHz)***	(300.13, 75.47)	***Nucleus***	(1H, 13C)
Number of Transients	32	***Origin***	spect	***Original Points Count***	(128, 64)	***Owner***	psm
Points Count	(1024, 512)	***Pulse Sequence***	hsqcedetgp	***Solvent***	DMSO	***Spectrum Type***	HSQC-DEPT
Sweep Width (Hz)	(2994.67, 12475.59)	***Temperature (degree C)***	19.760	***Title***	I8gCD_HSQC_DMSO-d6_290416_1832_RG=110		

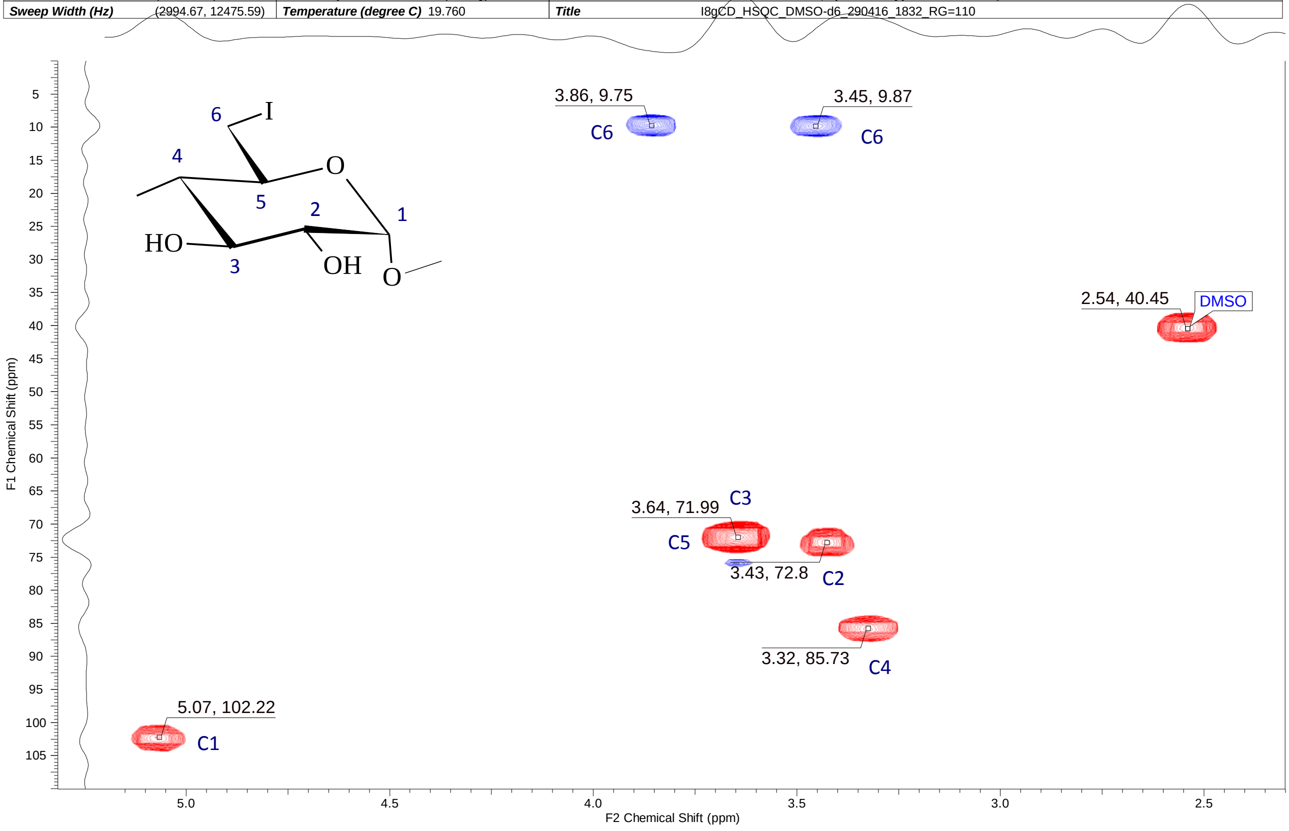

*Fig. S 26. HSQC-NMR spectrum of octakis(6-deoxy-6-iodo)-γCD **2b***

Acquisition Time (sec)	3.6438	***Comment***	Br8GCD_1H_DMSO-d6_100316_1746_RG=70			***Date***	11 Mar 2016 07:56:00
Date Stamp	11 Mar 2016 07:56:00	***File Name***	D:\Docs\!Notebooks\BM_Reactions\NMR\Br8GCD\1\fid			***Frequency (MHz)***	300.13
Nucleus	1H	***Number of Transients***	64	***Origin***	spect	***Original Points Count***	16384
Owner	root	***Points Count***	131072	***Pulse Sequence***	zg	***Receiver Gain***	90.50
SW(cyclical) (Hz)	4496.40	***Solvent***	DMSO-d6	***Spectrum Offset (Hz)***	1093.0117	***Spectrum Type***	STANDARD
Sweep Width (Hz)	4496.37	***Temperature (degree C)***	19.760				

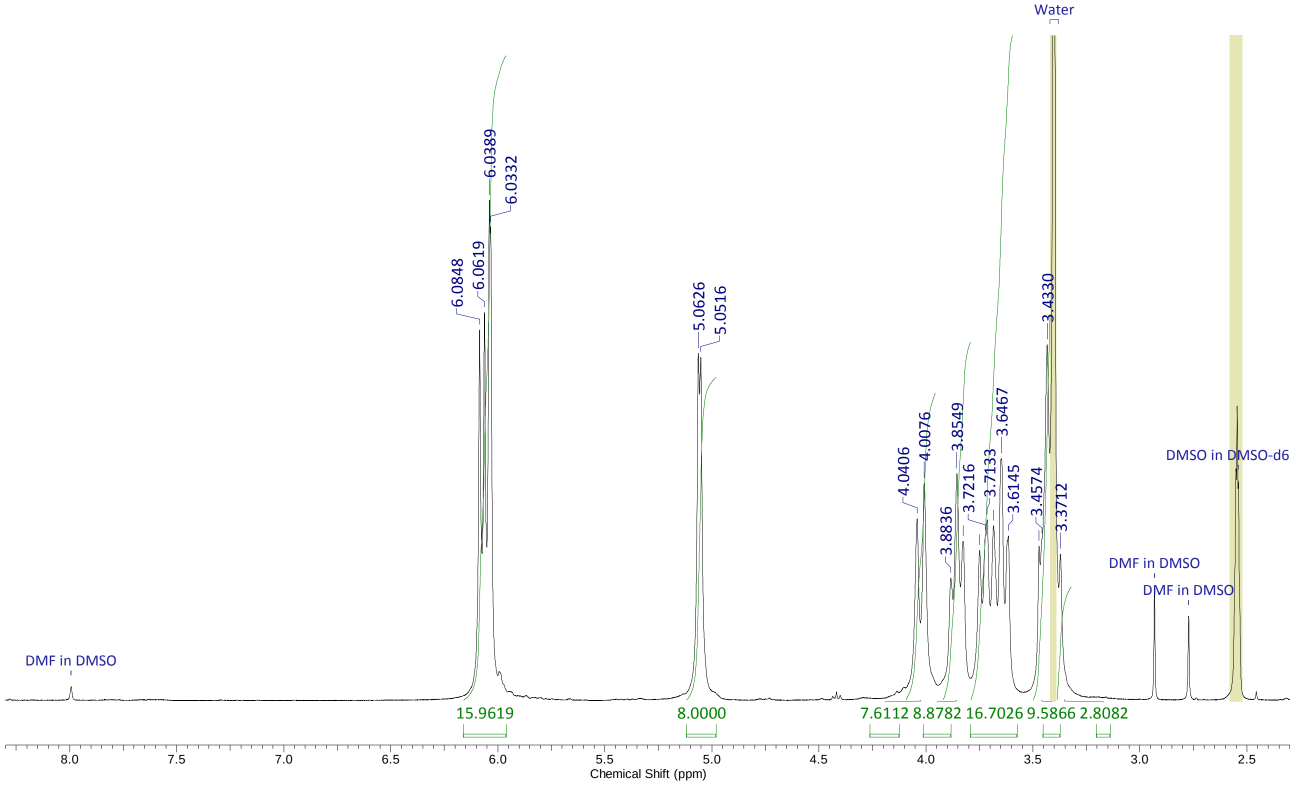

*Fig. S 27. ^{1}H-NMR spectrum of octakis(6-bromo-6-deoxy)-γCD **2b'***

Acquisition Time (sec)	(0.0427, 0.0051)	***Comment***	5 mm BBO 1H-BB Z-GRD Z8284/0059	***Date***	11 Mar 2016 09:51:06
File Name	D:\Docs\!Notebooks\BM_Reactions\NMR\Br8GCD\2\ser	***Frequency (MHz)***	(300.13, 75.47)	***Nucleus***	(1H, 13C)
Number of Transients	32	***Origin***	spect	***Original Points Count***	(128, 64)
Owner	psm	***Points Count***	(1024, 256)	***Pulse Sequence***	hsqcedetgp
Solvent	DMSO	***Spectrum Type***	HSQC-DEPT	***Sweep Width (Hz)***	(2994.67, 12451.17)
Temperature (degree C)	19.760	***Title***	Br8GCD_HSQC_DMSO-d6_100316_1746_RG=70		
AutoPhase	Ambiguous Phase				

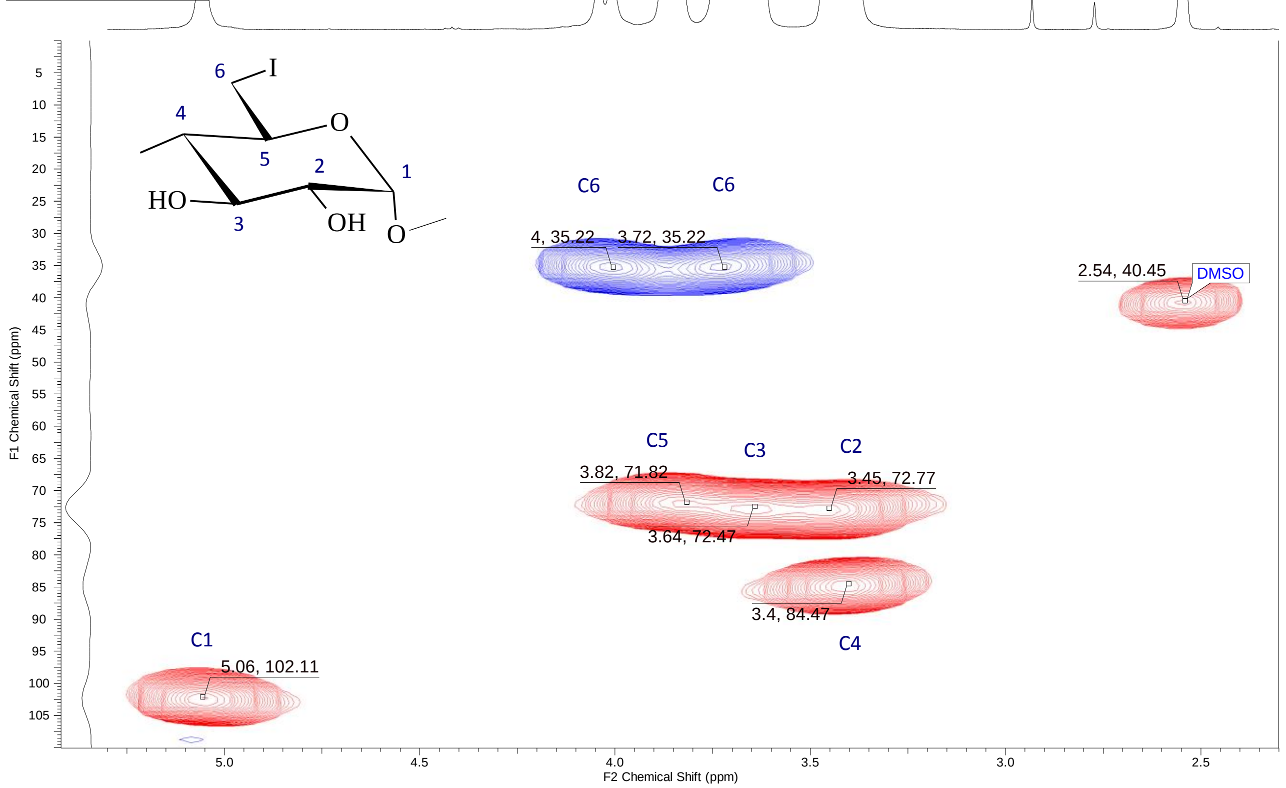

*Fig. S 28. HSQC-NMR spectrum of octakis(6-bromo-6-deoxy)-γCD **2b'***

Acquisition Time (sec)	3.6438	*Comment*	TU7BCDLJ01_1H_D2O_220416_1820_RG=140			*Date*	22 Apr 2016 10:23:28		
Date Stamp	22 Apr 2016 10:23:28			*File Name*	D:\Docs\!Notebooks\BM_Reactions\NMR\TU7BCDLJ01\1\fid				
Frequency (MHz)	300.13	*Nucleus*	1H	*Number of Transients*	64	*Origin*	spect	*Original Points Count*	16384
Owner	root	*Points Count*	65536	*Pulse Sequence*	zg	*Receiver Gain*	143.70	*SW(cyclical) (Hz)*	4496.40
Solvent	HDO in D2O	*Spectrum Offset (Hz)*	1096.3304	*Spectrum Type*	STANDARD	*Sweep Width (Hz)*	4496.33	*Temperature (degree C)*	20.860

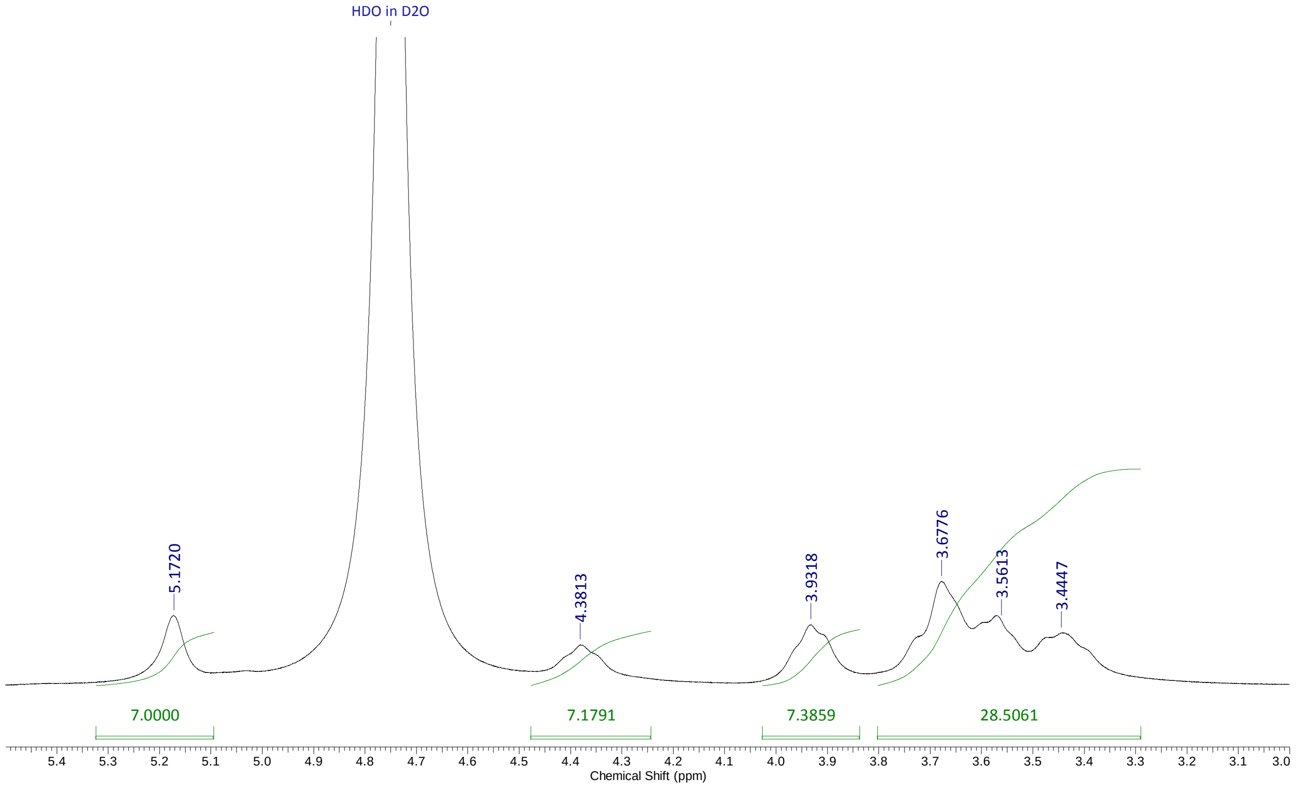

Fig. S 29. [1]*H-NMR spectrum of heptakis(6-deoxy-6-thioureido)-βCD iodide* ***3a****, entry 4, thiourea/I ratio=3.5*

Acquisition Time (sec)	(0.0427, 0.0051)	*Comment*	5 mm BBO 1H-BB Z-GRD Z8284/0059			*Date*	22 Apr 2016 11:16:44
File Name	D:\Docs\!Notebooks\BM_Reactions\NMR\TU7BCDLJ01\2\ser			*Frequency (MHz)*	(300.13, 75.47)	*Nucleus*	(1H, 13C)
Number of Transients	32	*Origin*	spect	*Original Points Count*	(128, 64)	*Owner*	psm
Points Count	(512, 256)	*Pulse Sequence*	hsqcedetgp	*Solvent*	DMSO	*Spectrum Type*	HSQC-DEPT
Sweep Width (Hz)	(2991.75, 12451.17)	*Temperature (degree C)*	20.860	*Title*	TU7BCDLJ01_HSQC_D2O_220416_1820_RG=140		

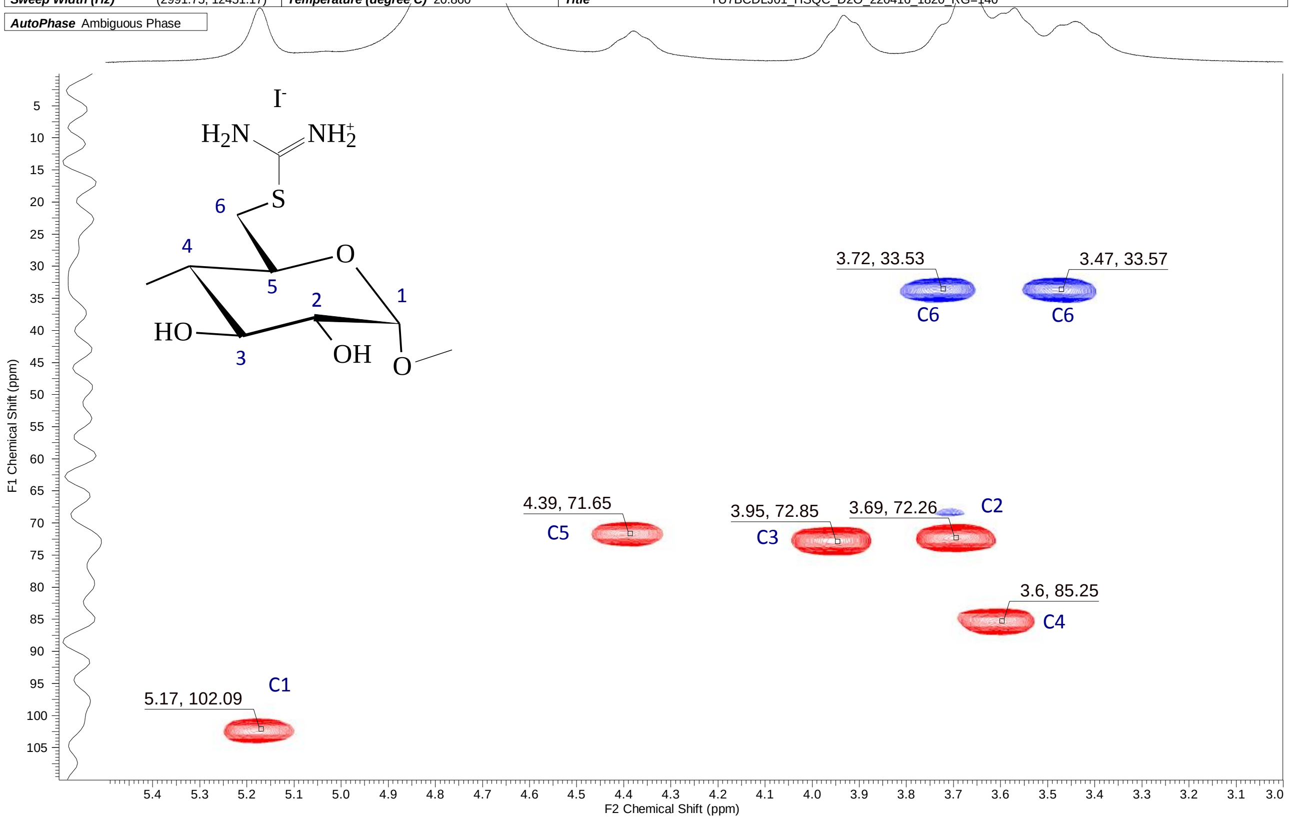

Fig. S 30. HSQC-NMR spectrum of heptakis(6-deoxy-6-thioureido)-βCD iodide ***3a****, entry 4, thiourea/I ratio=3.5*

Acquisition Time (sec)	3.6438	***Comment***	TU8CDLJ01_1H_D2O_250216_1707_RG=130			***Date***	25 Feb 2016 13:30:56		
Date Stamp	25 Feb 2016 13:30:56			***File Name***	D:\Docs\!Notebooks\BM_Reactions\NMR\TU8CDLJ01\1\fid			***Frequency (MHz)***	300.13
Nucleus	1H	***Number of Transients***	64	***Origin***	spect	***Original Points Count***	16384	***Owner***	root
Points Count	131072	***Pulse Sequence***	zg	***Receiver Gain***	128.00	***SW(cyclical) (Hz)***	4496.40	***Solvent***	HDO in D2O
Spectrum Offset (Hz)	1097.3718	***Spectrum Type***	STANDARD	***Sweep Width (Hz)***	4496.37	***Temperature (degree C)***	20.360		

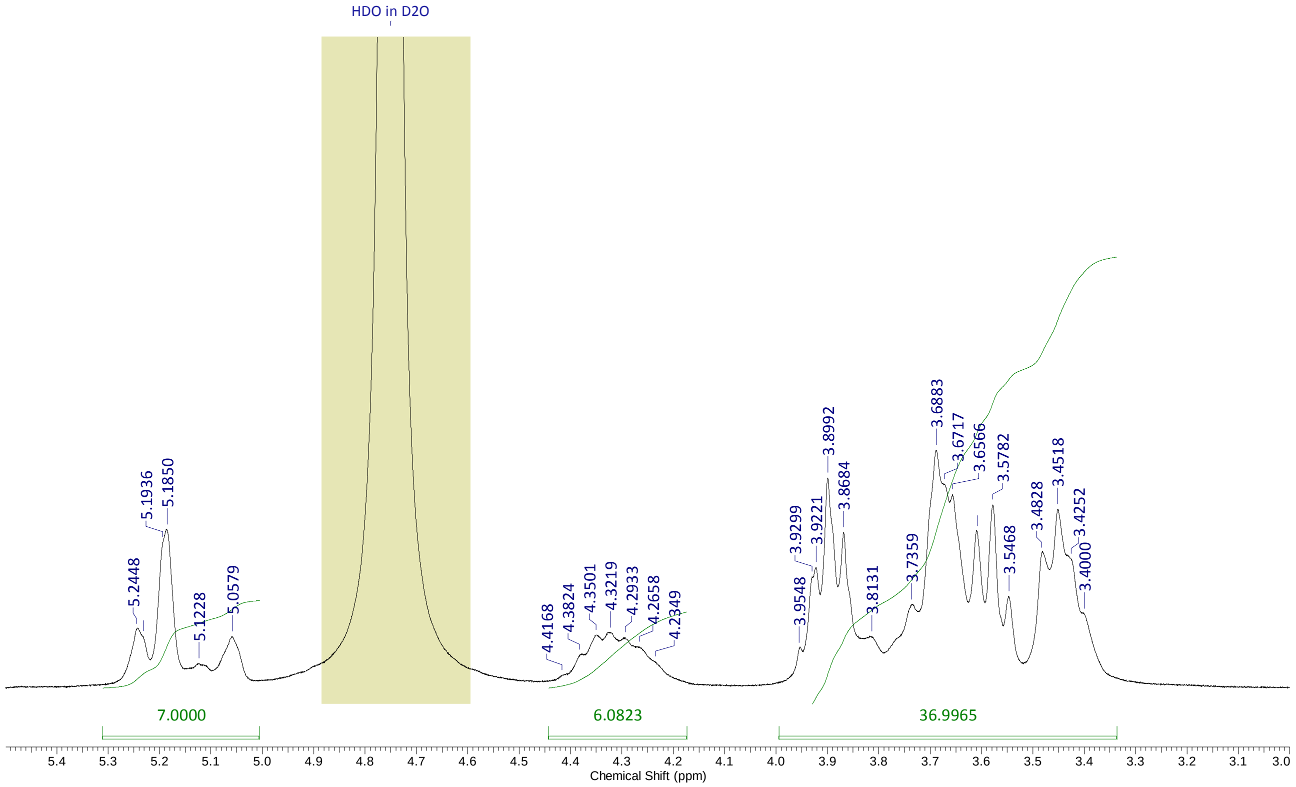

*Fig. S 31. ^{1}H-NMR spectrum of octakis(6-deoxy-6-thioureido)-γCD iodide **3b**, entry 7, thiourea/I ratio=1.5*

Acquisition Time (sec)	(0.0427, 0.0051)	***Comment***	5 mm BBO 1H-BB Z-GRD Z8284/0059			***Date***	25 Feb 2016 15:24:50
File Name	D:\Docs\!Notebooks\BM_Reactions\NMR\TU8CDLJ01\2\ser			***Frequency (MHz)***	(300.13, 75.47)	***Nucleus***	(1H, 13C)
Number of Transients	32	***Origin***	spect	***Original Points Count***	(128, 64)	***Owner***	psm
Points Count	(512, 256)	***Pulse Sequence***	hsqcedetgp	***Solvent***	DMSO	***Spectrum Type***	HSQC-DEPT
Sweep Width (Hz)	(2991.75, 12451.17)	***Temperature (degree C)***	20.460	***Title***	TU8CDLJ01_HSQC_D2O_250216_1707_RG=130		

AutoPhase Ambiguous Phase

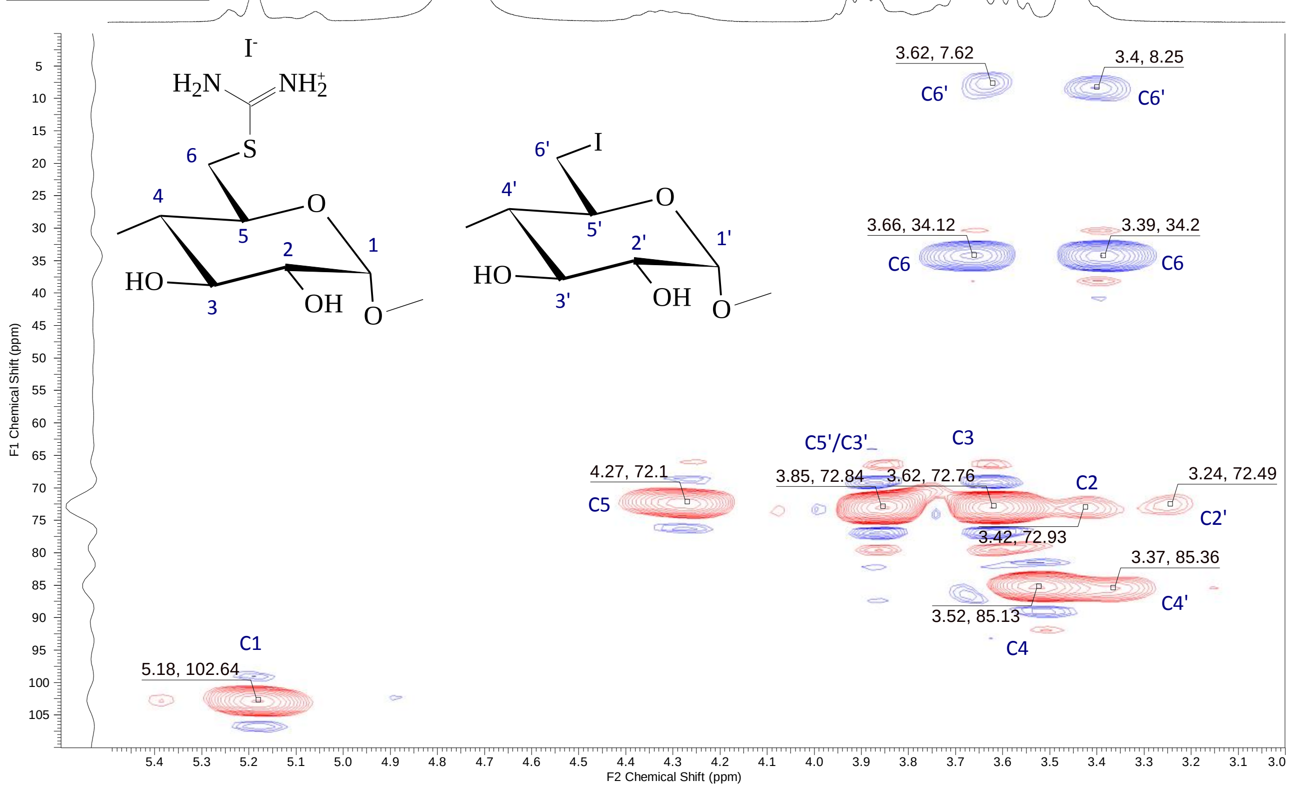

*Fig. S 32. HSQC-NMR spectrum of octakis(6-deoxy-6-thioureido)-γCD iodide **3b**, entry 7, thiourea/I ratio=1.5*

Acquisition Time (sec)	3.6438	*Comment*	TU8CD_LJ02_pur_1H_D2O_010316_1724_RG=140			*Date*	22 Apr 2016 14:26:40		
Date Stamp	22 Apr 2016 14:26:40			*File Name*	D:\Docs\!Notebooks\BM_Reactions\NMR\TU8CD_LJ02_pur\1\fid				
Frequency (MHz)	300.13	*Nucleus*	1H	*Number of Transients*	64	*Origin*	spect	*Original Points Count*	16384
Owner	root	*Points Count*	131072	*Pulse Sequence*	zg	*Receiver Gain*	181.00	*SW(cyclical) (Hz)*	4496.40
Solvent	HDO in D2O	*Spectrum Offset (Hz)*	1097.6119	*Spectrum Type*	STANDARD	*Sweep Width (Hz)*	4496.37	*Temperature (degree C)*	21.560

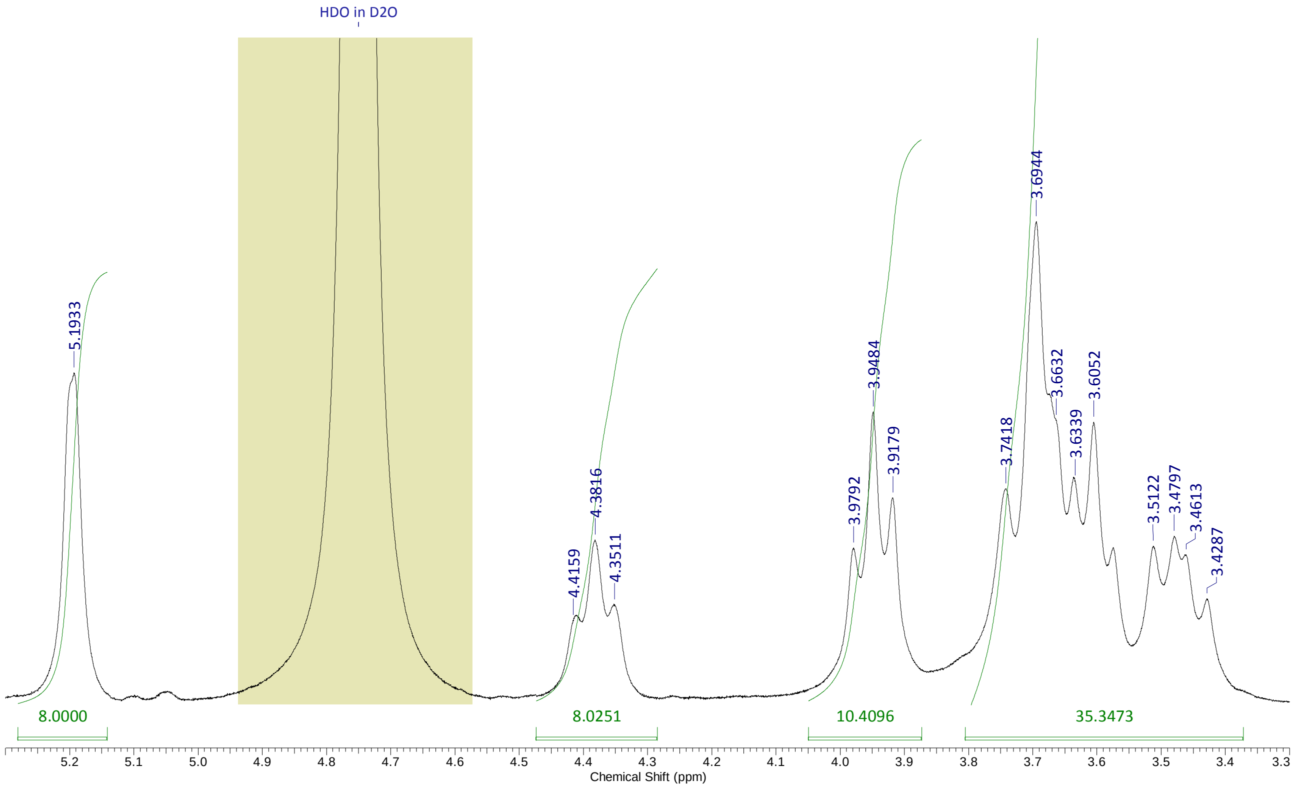

Fig. S 33. ^{1}H-NMR spectrum of octakis(6-deoxy-6-thioureido)-γCD iodide ***3b*** *entry 9, thiourea/I ratio=3.5*

Acquisition Time (sec)	(0.0427, 0.0051)	*Comment*	5 mm BBO 1H-BB Z-GRD Z8284/0059			*Date*	01 Mar 2016 16:28:18
File Name	D:\Docs\!Notebooks\BM_Reactions\NMR\TU8CD_LJ02_pur\2\ser			*Frequency (MHz)*	(300.13, 75.47)	*Nucleus*	(1H, 13C)
Number of Transients	32	*Origin*	spect	*Original Points Count*	(128, 64)	*Owner*	psm
Points Count	(1024, 512)	*Pulse Sequence*	hsqcedetgp	*Solvent*	DMSO	*Spectrum Type*	HSQC-DEPT
Sweep Width (Hz)	(2994.67, 12475.59)	*Temperature (degree C)*	20.160	*Title*	TU8CDpur_HSQC_D2O_010316_1724_RG=130		
AutoPhase Ambiguous Phase							

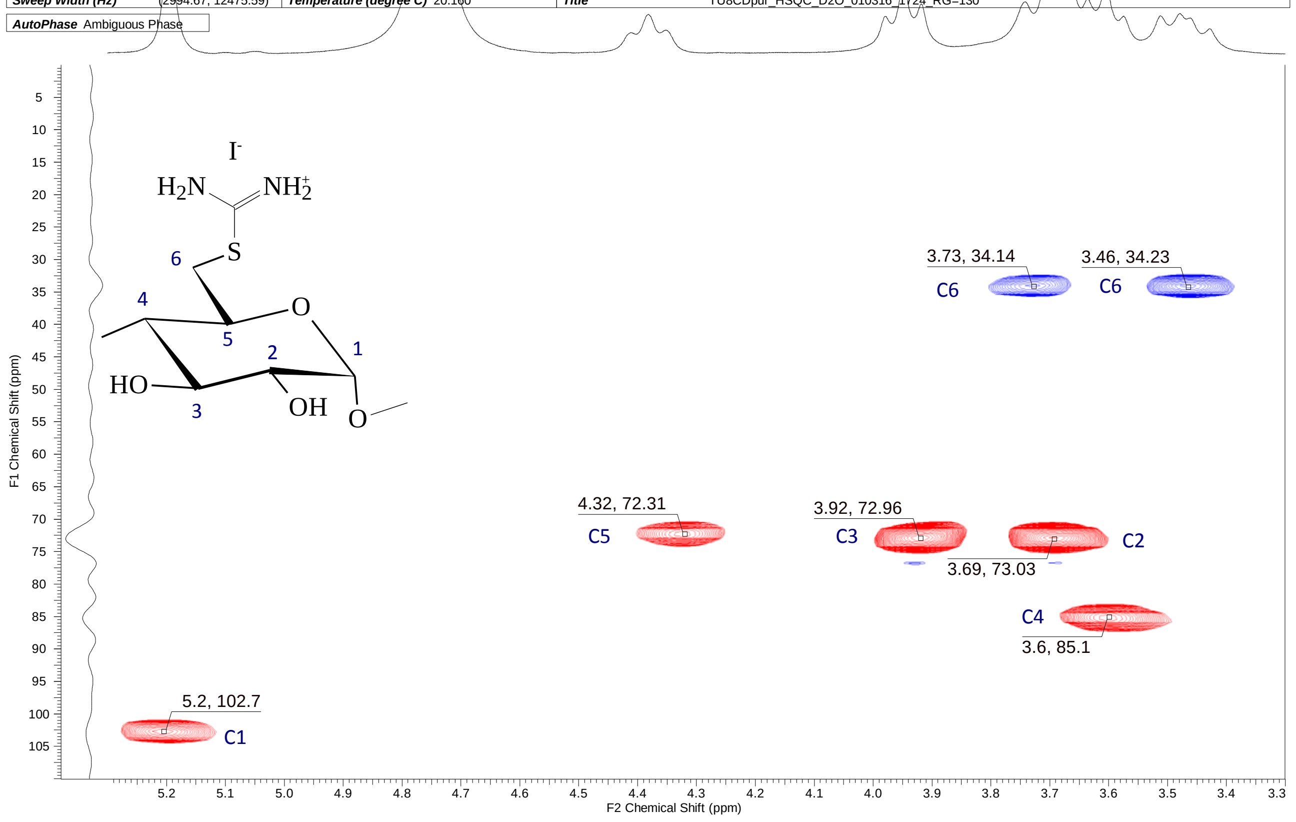

Fig. S 34. HSQC-NMR spectrum of octakis(6-deoxy-6-thioureido)-γCD iodide ***3b****, entry 9, thiourea/I ratio=3.5*

Acquisition Time (sec)	3.6438	***Comment***	TU8GCDLJ03_1H_D2O_090316_1733_RG=90			***Date***	09 Mar 2016 08:02:24
Date Stamp	09 Mar 2016 08:02:24			***File Name***	D:\Docs\!Notebooks\BM_Reactions\NMR\TU8GCDLJ03\TU8GCDLJ03.001.jdx		
Frequency (MHz)	300.13	***Nucleus***	1H	***Origin***	spect	***Original Points Count***	16384
Owner	root	***Points Count***	65536	***SW(cyclical) (Hz)***	4496.40	***Solvent***	D2O
Spectrum Offset (Hz)	1098.1777	***Sweep Width (Hz)***	4496.33				

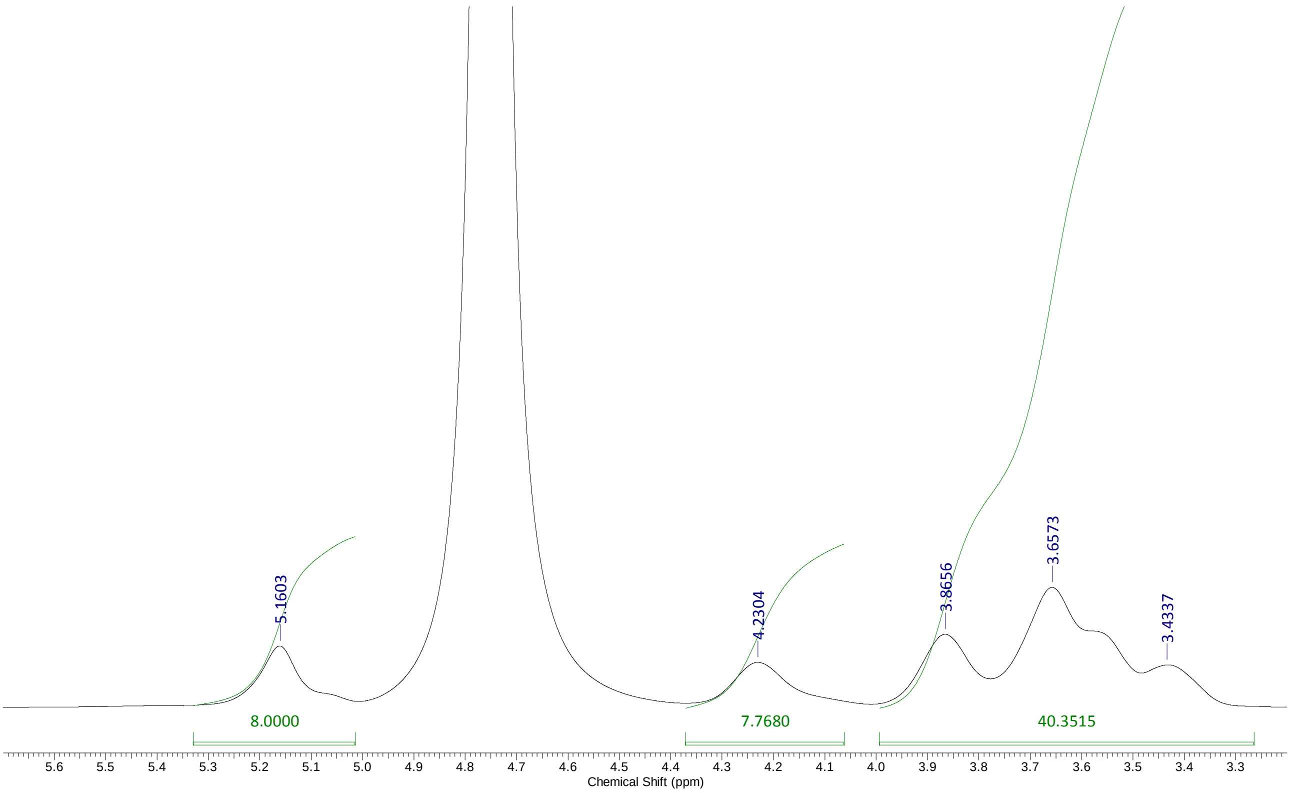

*Fig. S 35. ^{1}H-NMR spectrum of octakis(6-deoxy-6-thioureido)-γCD bromide **3b'**, entry 11, thiourea/I ratio=3.5*

Acquisition Time (sec)	(0.0427, 0.0051)	***Comment***	5 mm BBO 1H-BB Z-GRD Z8284/0059			***Date***	09 Mar 2016 09:55:58
File Name	D:\Docs\!Notebooks\BM_Reactions\NMR\TU8GCDLJ03\2\ser			***Frequency (MHz)***	(300.13, 75.47)	***Nucleus***	(1H, 13C)
Number of Transients	32	***Origin***	spect	***Original Points Count***	(128, 64)	***Owner***	psm
Points Count	(512, 512)	***Pulse Sequence***	hsqcedetgp	***Solvent***	DMSO	***Spectrum Type***	HSQC-DEPT
Sweep Width (Hz)	(2991.75, 12475.59)			***Temperature (degree C)***	19.460		

AutoPhase	Ambiguous Phase

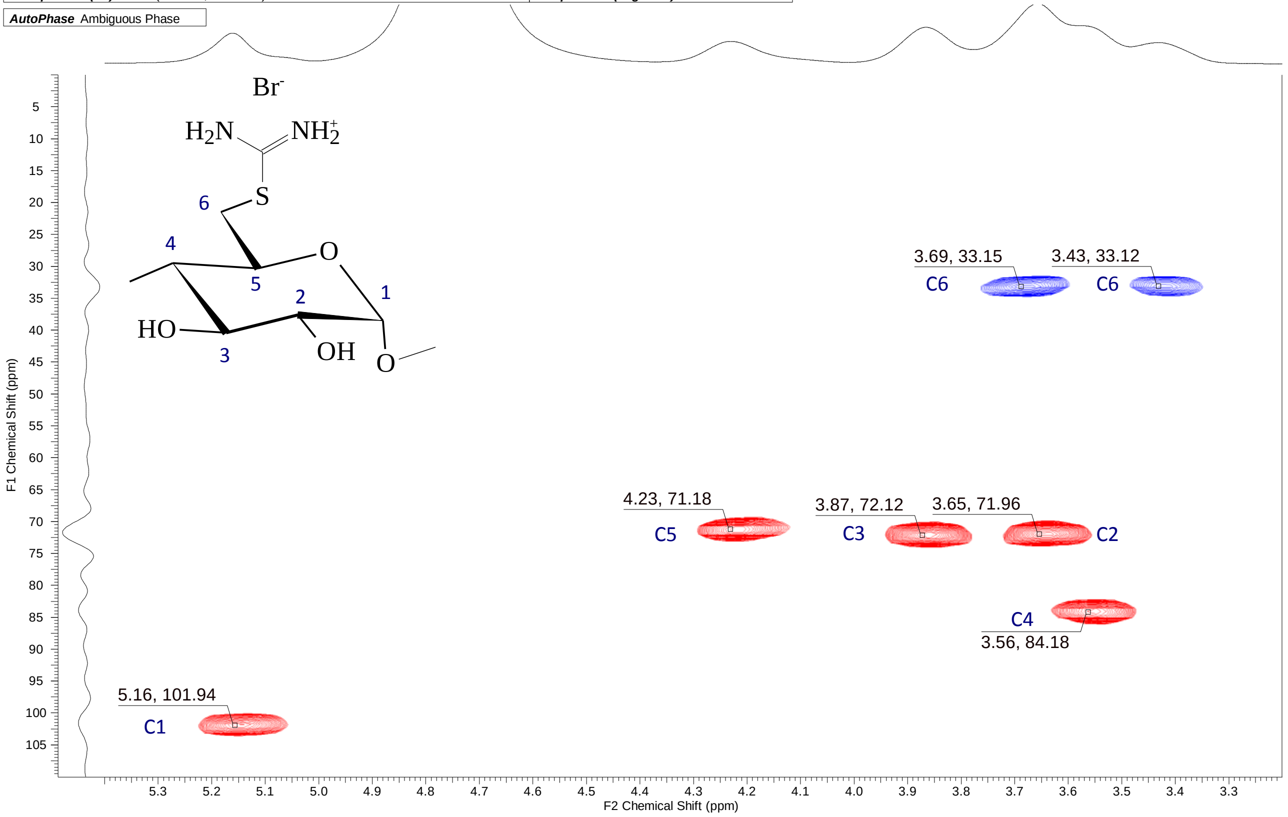

*Fig. S 36. HSQC-NMR spectrum of octakis(6-deoxy-6-thioureido)-γCD bromide **3b'**, entry 11, thiourea/I ratio=3.5*

Acquisition Time (sec)	3.6438	***Comment***	AZ7BCDLJ03_1H_DMSO-d6_080316_1733_RG=140			***Date***	08 Mar 2016 13:37:20		
Date Stamp	08 Mar 2016 13:37:20			***File Name***	D:\Docs\!Notebooks\BM_Reactions\NMR\AZ7BCDLJ03\1\fid			***Frequency (MHz)***	300.13
Nucleus	1H	***Number of Transients***	64	***Origin***	spect	***Original Points Count***	16384	***Owner***	root
Points Count	131072	***Pulse Sequence***	zg	***Receiver Gain***	143.00	***SW(cyclical) (Hz)***	4496.40	***Solvent***	DMSO
Spectrum Offset (Hz)	1063.3256	***Spectrum Type***	STANDARD	***Sweep Width (Hz)***	4496.37	***Temperature (degree C)***	19.760		

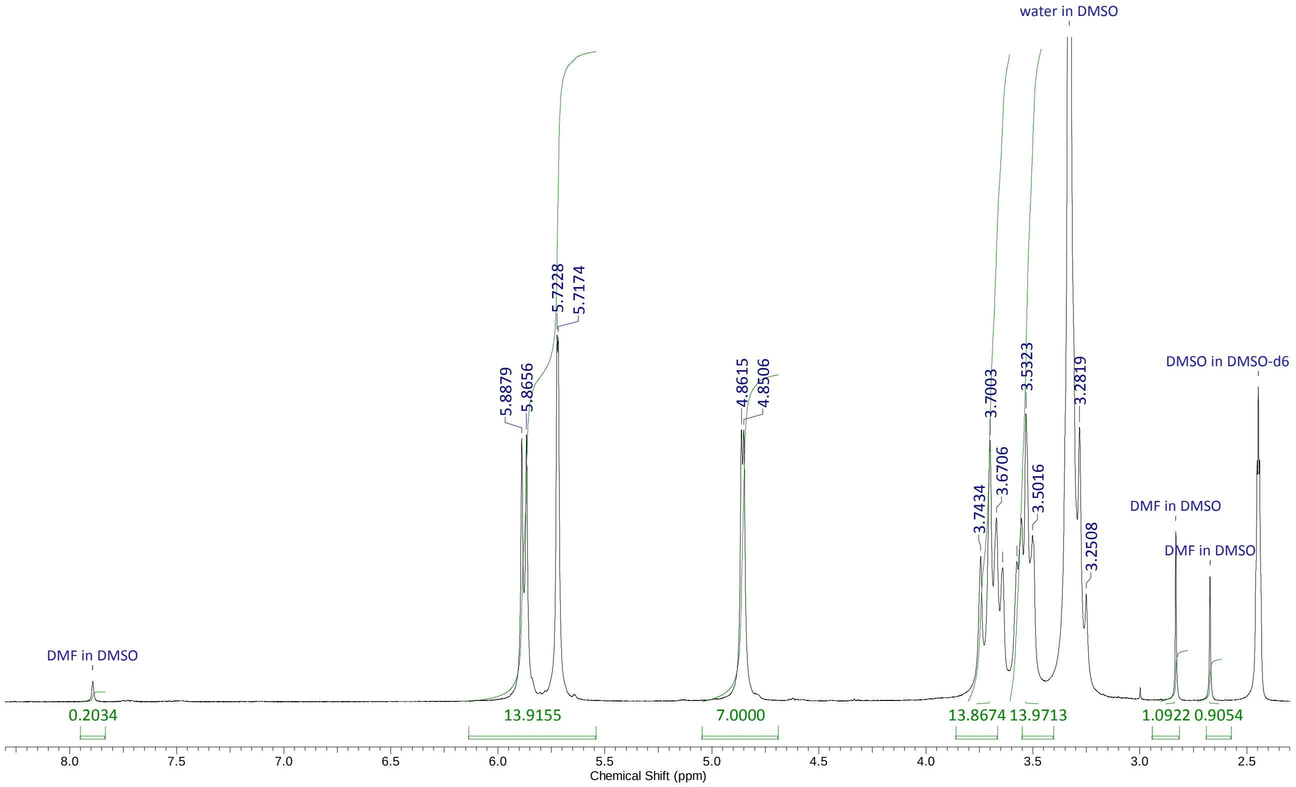

*Fig. S 37. ^{1}H-NMR spectrum of heptakis(6-azido-6-deoxy)-βCD **4a** entry 17*

Acquisition Time (sec)	(0.0427, 0.0051)	***Comment***	5 mm BBO 1H-BB Z-GRD Z8284/0059			***Date***	08 Mar 2016 15:30:58
File Name	D:\Docs\!Notebooks\BM_Reactions\NMR\AZ7BCDLJ03\2\ser			***Frequency (MHz)***	(300.13, 75.47)	***Nucleus***	(1H, 13C)
Number of Transients	32	***Origin***	spect	***Original Points Count***	(128, 64)	***Owner***	psm
Points Count	(512, 256)	***Pulse Sequence***	hsqcedetgp	***Solvent***	DMSO	***Spectrum Type***	HSQC-DEPT
Sweep Width (Hz)	(2991.75, 12451.17)	***Temperature (degree C)***	19.860	***Title***	AZ7BCDLJ03_HSQC_DMSO-d6_080316_1733_RG=140		
AutoPhase Ambiguous Phase							

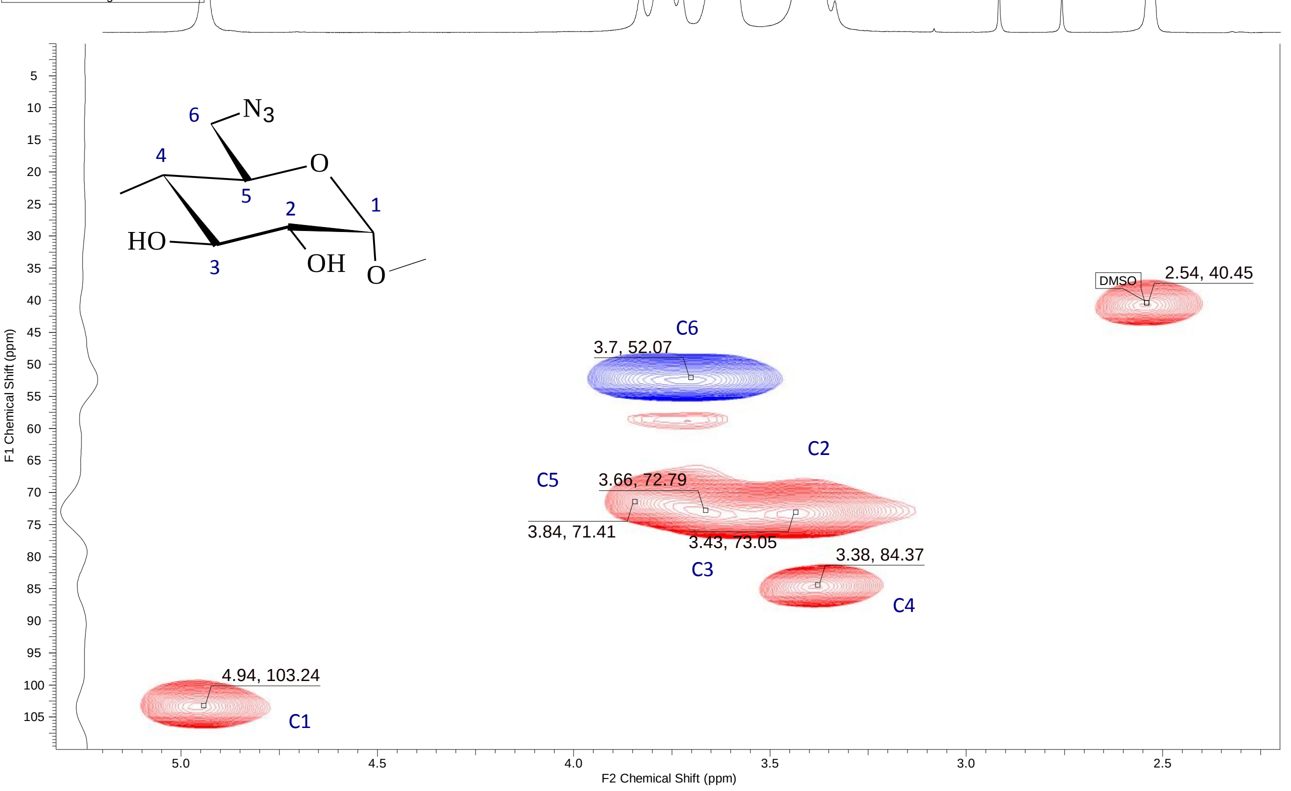

*Fig. S 38. HSQC-NMR spectrum of heptakis(6-azido-6-deoxy)-βCD **4a** entry 17*

Acquisition Time (sec)	3.6438	***Comment***	AZ8GCD LJ04_1H_DMSO-d6_100316_1745_RG=70			***Date***	10 Mar 2016 14:56:16		
Date Stamp	10 Mar 2016 14:56:16			***File Name***	D:\Docs\!Notebooks\BM_Reactions\NMR\AZ8GCD LJ04\1\fid				
Frequency (MHz)	300.13	***Nucleus***	1H	***Number of Transients***	64	***Origin***	spect	***Original Points Count***	16384
Owner	root	***Points Count***	131072	***Pulse Sequence***	zg	***Receiver Gain***	71.80	***SW(cyclical) (Hz)***	4496.40
Solvent	DMSO	***Spectrum Offset (Hz)***	1091.5365	***Spectrum Type***	STANDARD	***Sweep Width (Hz)***	4496.37	***Temperature (degree C)***	19.960

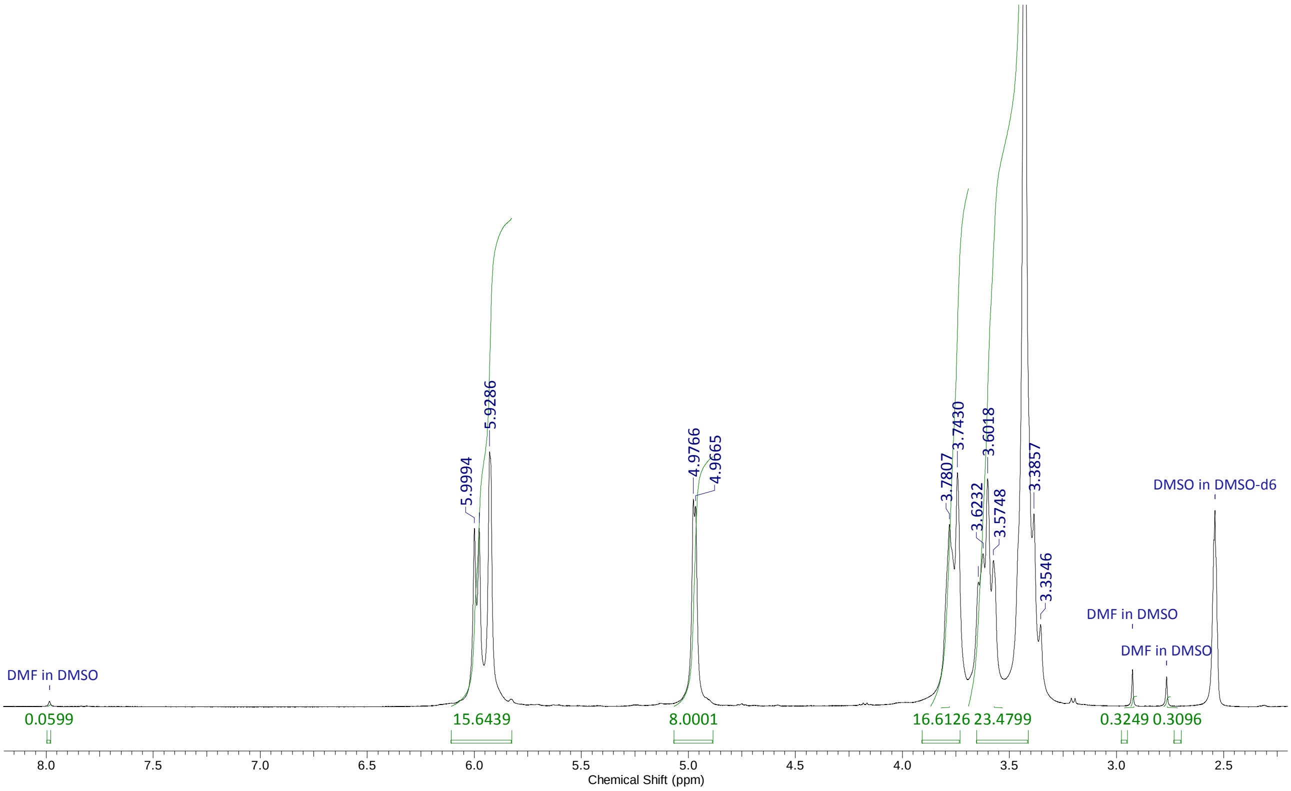

*Fig. S 39. ^{1}H-NMR spectrum of octakis(6-azido-6-deoxy)-γCD **4b** entry 20*

Acquisition Time (sec)	(0.0427, 0.0051)	***Comment***	5 mm BBO 1H-BB Z-GRD Z8284/0059			***Date***	10 Mar 2016 16:49:26
File Name	D:\Docs\!Notebooks\BM_Reactions\NMR\AZ8GCD LJ04\2\ser			***Frequency (MHz)***	(300.13, 75.47)	***Nucleus***	(1H, 13C)
Number of Transients	32	***Origin***	spect	***Original Points Count***	(128, 64)	***Owner***	psm
Points Count	(1024, 512)	***Pulse Sequence***	hsqcedetgp	***Solvent***	DMSO	***Spectrum Type***	HSQC-DEPT
Sweep Width (Hz)	(2994.67, 12475.59)	***Temperature (degree C)***	19.960	***Title***	AZ8GCD LJ04_HSQC_DMSO-d6_100316_1745_RG=70		

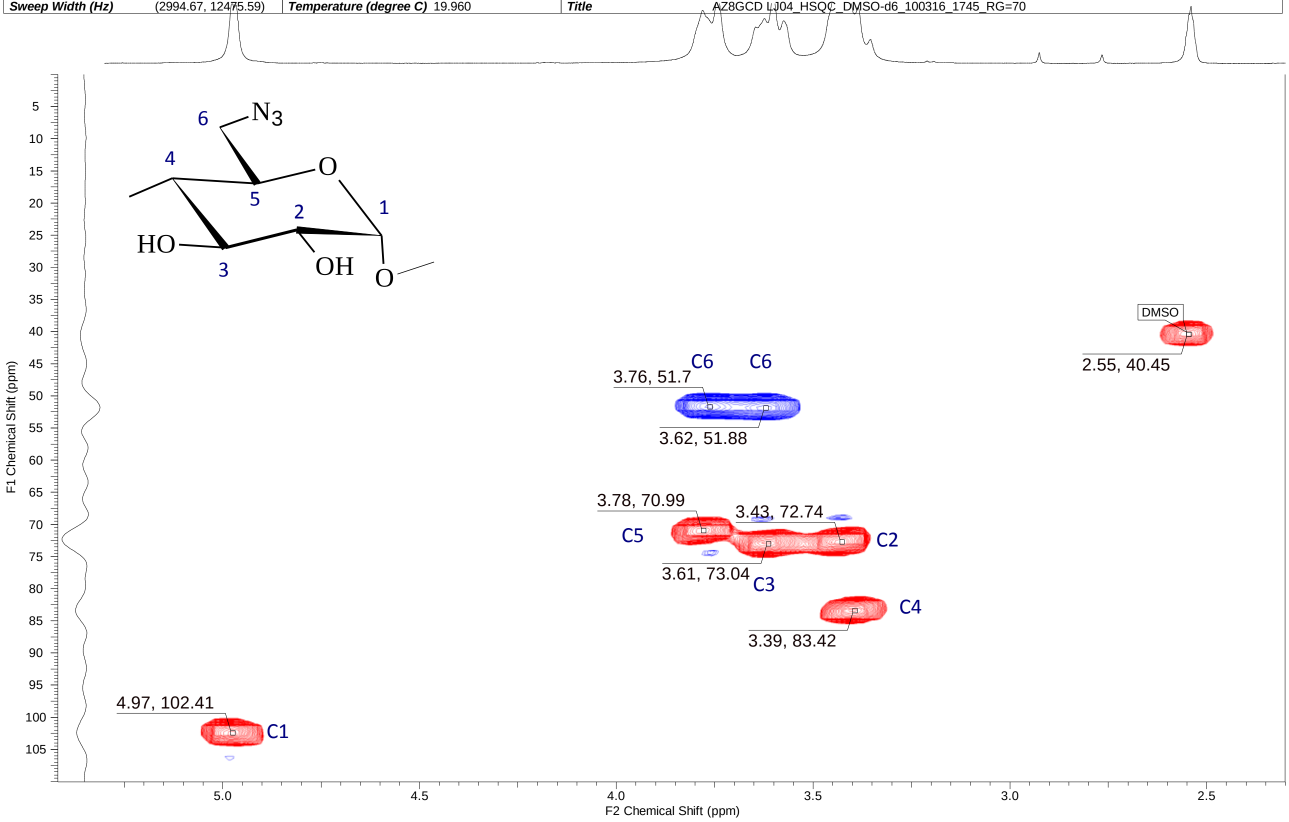

*Fig. S 40. HSQC-NMR spectrum of octakis(6-azido-6-deoxy)-γCD **4b** entry 20*

Acquisition Time (sec)	3.6438	***Comment***	CG34purSUBE_1H_D2O_18022016_1687			***Date***	18 Feb 2016 12:54:40		
Date Stamp	18 Feb 2016 12:54:40			***File Name***	D:\Docs\!Notebooks\BM_Reactions\NMR\CG34purSUBE\1\fid				
Frequency (MHz)	300.13	***Nucleus***	1H	***Number of Transients***	64	***Origin***	spect	***Original Points Count***	16384
Owner	root	***Points Count***	131072	***Pulse Sequence***	zg	***Receiver Gain***	161.30	***SW(cyclical) (Hz)***	4496.40
Solvent	HDO in D2O	***Spectrum Offset (Hz)***	1097.9205	***Spectrum Type***	STANDARD	***Sweep Width (Hz)***	4496.37	***Temperature (degree C)***	18.960

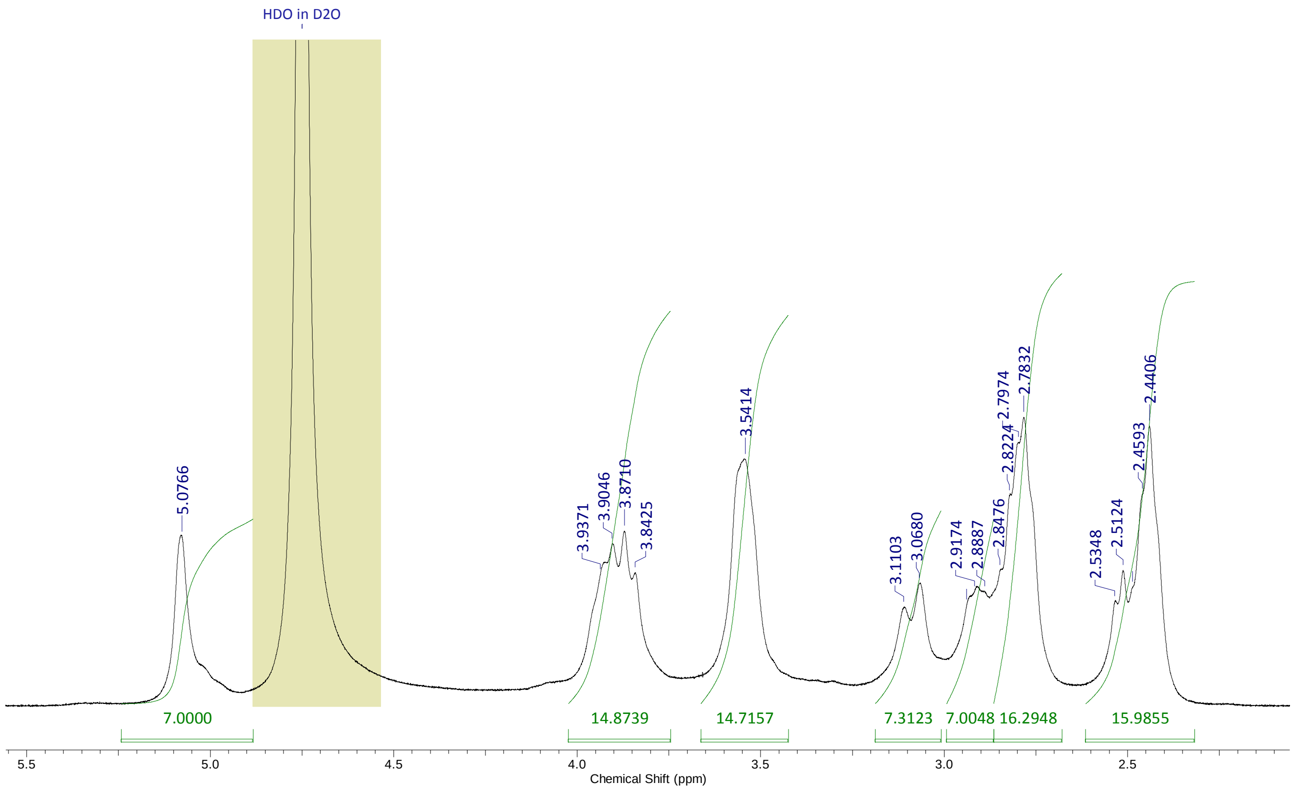

*Fig. S 41. ^{1}H-NMR spectrum of heptakis(6-deoxy-6-S-(3-mercapto)propionyl)-βCD NH_4^+ **5a** entry 23*

Acquisition Time (sec)	(0.0427, 0.0051)	***Comment***	5 mm BBO 1H-BB Z-GRD Z8284/0059			***Date***	19 Feb 2016 16:02:04
File Name	D:\Docs\!Notebooks\BM_Reactions\NMR\CG34purSUBE\4\ser			***Frequency (MHz)***	(300.13, 75.47)	***Nucleus***	(1H, 13C)
Number of Transients	32	***Origin***	spect	***Original Points Count***	(128, 64)	***Owner***	psm
Points Count	(512, 512)	***Pulse Sequence***	hsqcedetgp	***Solvent***	D2O	***Spectrum Type***	HSQC-DEPT
Sweep Width (Hz)	(2991.75, 12475.59)	***Temperature (degree C)***	20.060	***Title***	CG34purSUBEML_HSQC_D2O_190216_1694_RG=230		

AutoPhase Ambiguous Phase

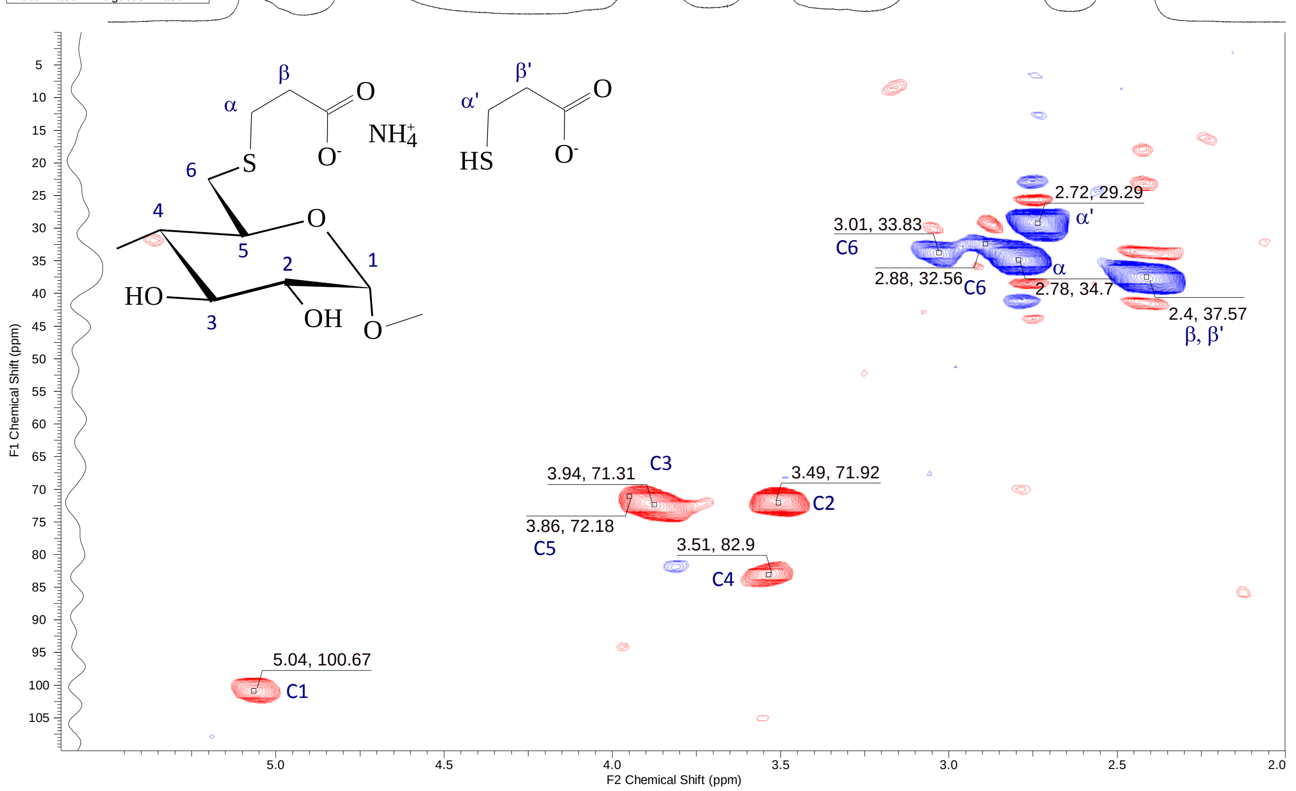

*Fig. S 42. HSQC-NMR spectrum of heptakis(6-deoxy-6-S-(3-mercapto)propionyl)-βCD NH_4^+ **5a** entry 23*

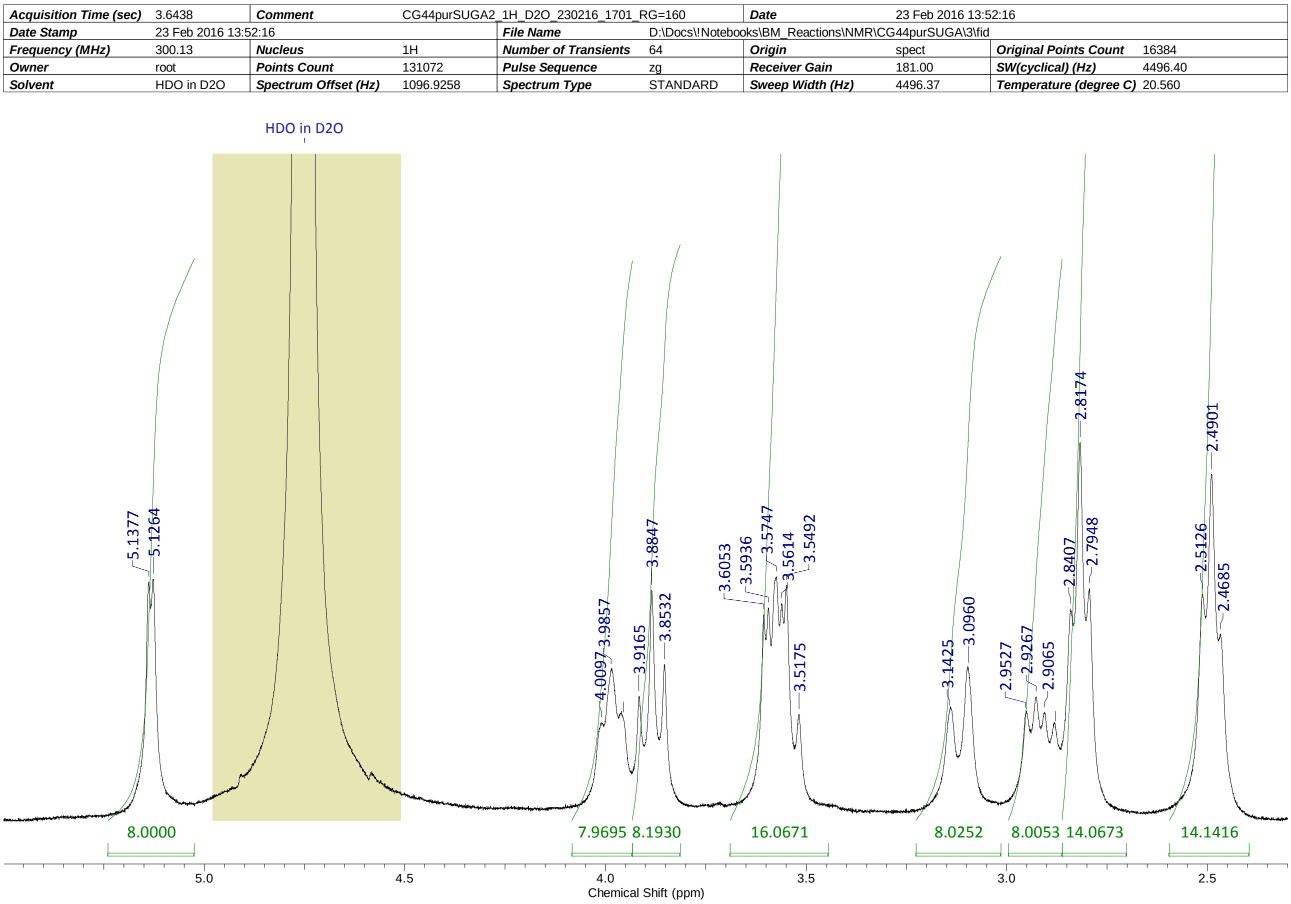

*Fig. S 43. ^{1}H-NMR spectrum of octakis(6-deoxy-6-S-(3-mercapto)propionyl)-γCD NH_4^+ **5b** entry 28*

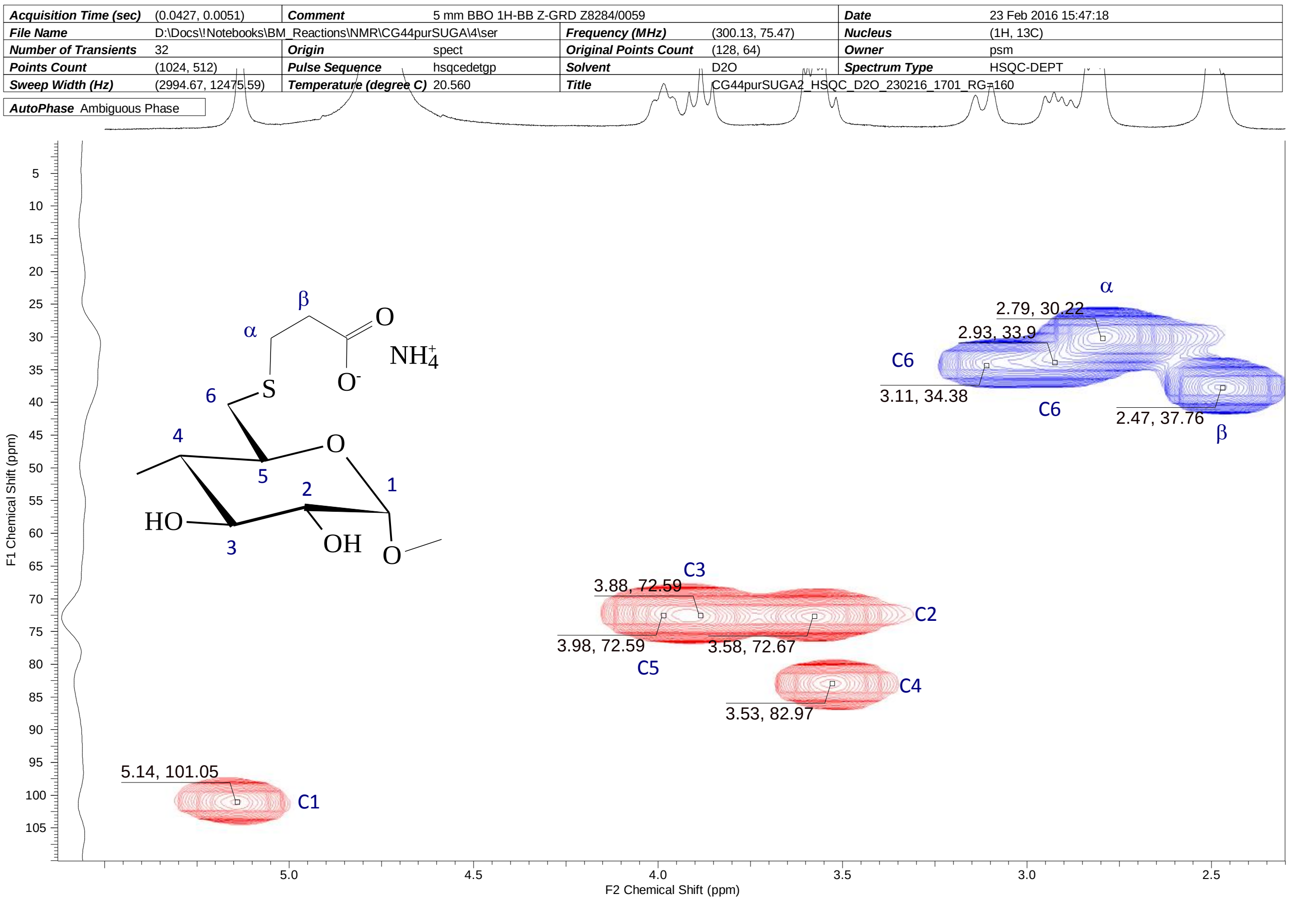

*Fig. S 44. HSQC-NMR spectrum of octakis(6-deoxy-6-S-(3-mercapto)propionyl)-γCD NH_4^+ **5b** entry 28*

Acquisition Time (sec)	3.6438	Comment	DDS7BCD-CGhqb_1H_DMSO-d6+CDCl3_050516_1846_RG=230	Date	05 May 2016 15:30:40
Date Stamp	05 May 2016 15:30:40	File Name	D:\Docs\!Notebooks\BM_Reactions\NMR\DDS7BCD-CG49\3\fid		

Frequency (MHz)	300.13	Nucleus	1H	Number of Transients	64	Origin	spect	Original Points Count	16384
Owner	root	Points Count	131072	Pulse Sequence	zg	Receiver Gain	228.10	SW(cyclical) (Hz)	4496.40
Solvent	DMSO-CDCl3	Spectrum Offset (Hz)	1091.2964	Spectrum Type	STANDARD	Sweep Width (Hz)	4496.37	Temperature (degree C)	20.860

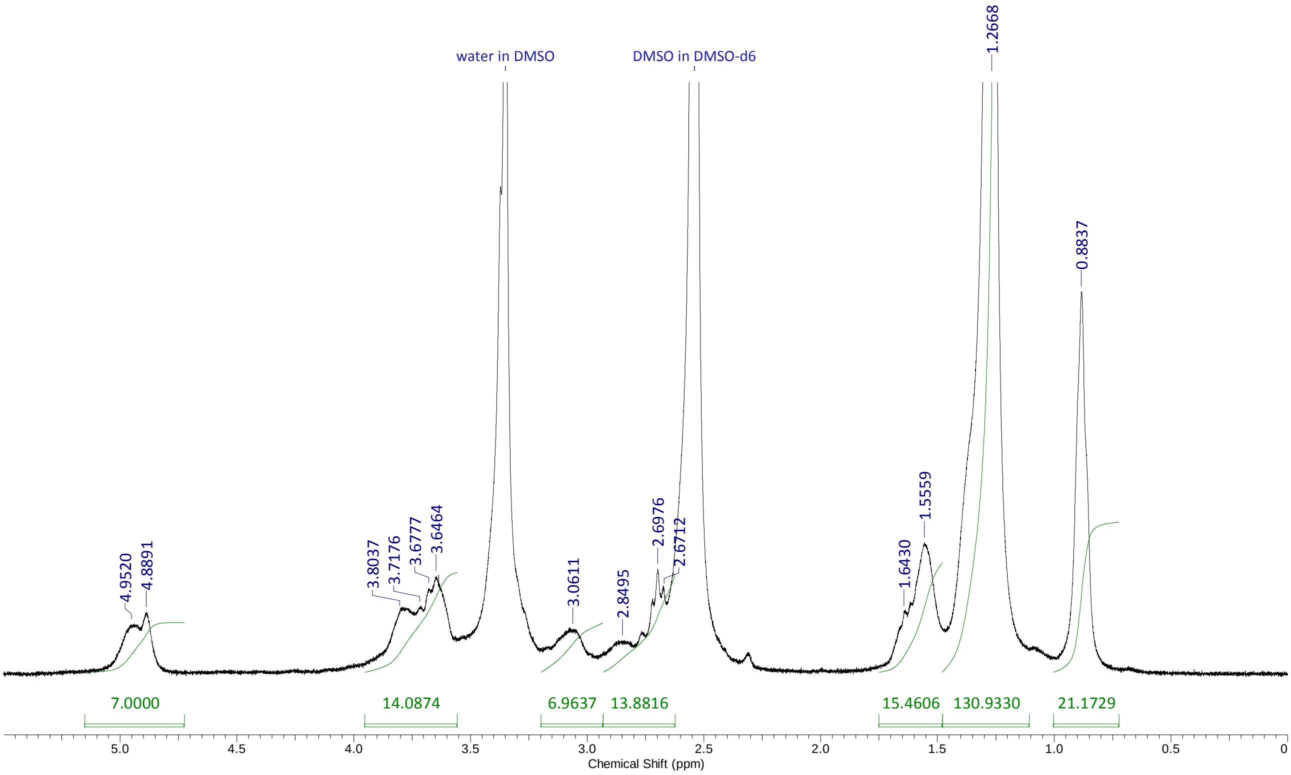

*Fig. S 45. ^{1}H-NMR spectrum of heptakis(6-deoxy-6-S-(1-dodecylthio)-βCD **6** entry 31*

Acquisition Time (sec)	(0.0427, 0.0051)	Comment	5 mm BBO 1H-BB Z-GRD Z8284/0059			Date	05 May 2016 16:25:18
File Name	D:\Docs\!Notebooks\BM_Reactions\NMR\DDS7BCD-CG49\4\ser			Frequency (MHz)	(300.13, 75.47)	Nucleus	(1H, 13C)
Number of Transients	32	Origin	spect	Original Points Count	(128, 64)	Owner	psm
Points Count	(2048, 512)	Pulse Sequence	hsqcedetgp	Solvent	DMSO-CDCl3	Spectrum Type	HSQC-DEPT
Sweep Width (Hz)	(2996.14, 12475.59)	Temperature (degree C)	20.860	Title	DDS7BCD-CGhqb_HSQC_DMSO-d6+CDCl3_050516_1846_RG=230		

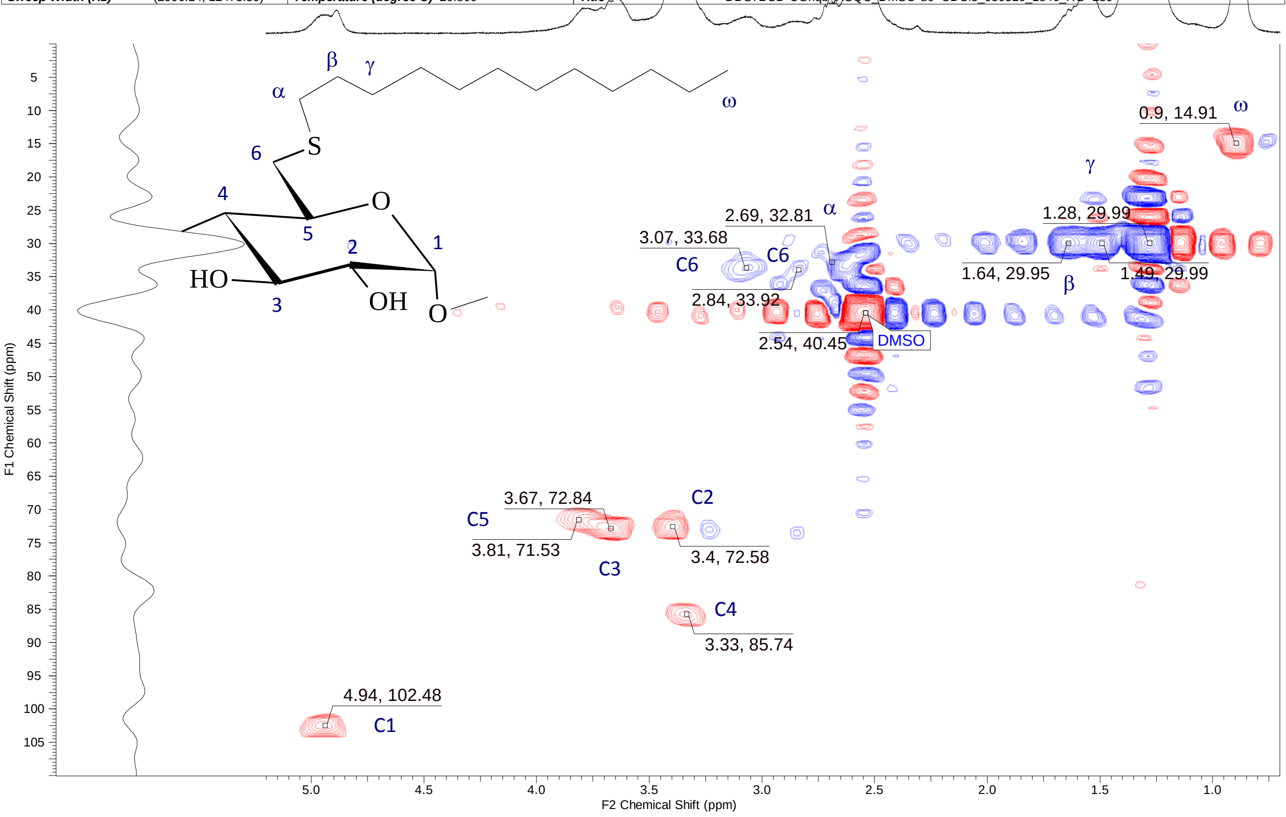

*Fig. S 46. HSQC-NMR spectrum of heptakis(6-deoxy-6-S-(1-dodecylthio)-βCD **6** entry 31*

References

1. Gadelle, A.; Defaye, J. *Angew. Chem. Int. Ed. Eng.*, **1991**, *30*, 78-80.
2. Tuza, K. Synthesis of Cyclodextrin based C. Perfringens Antidotes. (Hung), Master's Thesis Univ. L. Eötvös, Budapest, Hungary, 2011
3. Uccello-Barretta, G.; Evangelisti, C.; Balzano, F.; Vanni, L.; Aiello, F.; Jicsinszky, L. *Carbohydr. Res.*, **2011**, *346*, 753-758.
4. Szurmai, Z.; Lipták, A.; Debrecen; Szejtli, J. *Starch - Stärke*, **1990**, *42*, 447-449.
5. Adam, J. M.; Bennett, D. J.; Bom, A.; Clark, J. K.; Feilden, H.; Hutchinson, E. J.; Palin, R.; Prosser, A.; Rees, D. C.; Rosair, G. M.; Stevenson, D.; Tarver, G. J.; Zhang, M.-Q. *J. Med. Chem.*, **2002**, *45*, 1806-1816.
6. Bom, A.; Bradley, M.; Cameron, K.; Clark, J. K.; Egmond, J. van; Feilden, H.; MacLean, E. J.; Muir, A. W.; Palin, R.; Rees, D. C.; Zhang, M.-Q. *Angew. Chem.*, **2002**, *114*, 275-280.
7. Mazzaglia, A.; Donohue, R.; Ravoo, B. J.; Darcy, R. *Eur. J. Org. Chem.*, **2001**, *2001*, 1715-1721.
8. Ling, C.-C.; Darcy, R.; Risse, W. *J. Chem. Soc., Chem. Commun.*, **1993**, 438-440.
9. Darcy, R.; Penkler, L., US20060148756, July 6, 2006
